# Ancient Human Migration after Out-of-Africa

**DOI:** 10.1038/srep26565

**Published:** 2016-05-23

**Authors:** Daniel Shriner, Fasil Tekola-Ayele, Adebowale Adeyemo, Charles N. Rotimi

**Affiliations:** 1 Center for Research on Genomics and Global Health National Human Genome Research Institute, Building 12A, Room 4047 12 South Drive, Bethesda, Maryland 20892, USA

## Abstract

The serial founder model of modern human origins predicts that the phylogeny of ancestries exhibits bifurcating, tree-like behavior. Here, we tested this prediction using three methods designed to investigate gene flow in autosome-wide genotype data from 3,528 unrelated individuals from 163 global samples. Specifically, we investigated whether Cushitic ancestry has an East African or Middle Eastern origin. We found evidence for non-tree-like behavior in the form of four migration events. First, we found that Cushitic ancestry is a mixture of ancestries closely related to Arabian ancestry and Nilo-Saharan or Omotic ancestry. We found evidence for additional migration events in the histories of: 1) Indian and Arabian ancestries, 2) Kalash ancestry, and 3) Native American and Northern European ancestries. These findings, based on analysis of ancestry of present-day humans, reveal migration in the distant past and provide new insights into human history.

Genetic data provide insight into the migratory history and geographic structuring of modern human populations. The recent origin of modern humans reflects migration from sub-Saharan Africa, with the oldest divergence event at approximately 140,000 years ago evident from analysis of Y DNA haplogroups^1^,^2^ and autosomal markers^3^. Subsequently, at least 19 ancestries arose as humans migrated across the continents^3^,^4^. These ancestries reflect shared history at a level higher than populations, tribes, or ethnic groups. The divergence of ancestries is mainly due to random genetic drift subsequent to a barrier to random mating, with two common barriers being geographic and linguistic, following serial founder effects as modern humans peopled the world^5^.

Although most ancestries showed topological consistency in phylogenetic relationships assuming a strictly bifurcating or tree-like process, Cushitic ancestry showed unstable placement between East African and Middle Eastern ancestries^3^,^4^,^6^ and Melanesian ancestry showed an unexpectedly long branch^3^. These findings suggested the presence of non-tree-like behavior, *i.e*., some ancestries may have formed due to a mixture process rather than a splitting process. Here, we investigate this possibility using three different approaches: analysis of the distance matrix using split decomposition analysis^7^, analysis of ancestry-specific allele frequencies using *f*_3_ and *f*_4_ statistics^8^, and analysis of ancestry-specific allele frequencies using a graph-based model of gene flow^9^. We find evidence for non-tree-like behavior in the form of four migration events.

## Results

**Figure 1:**
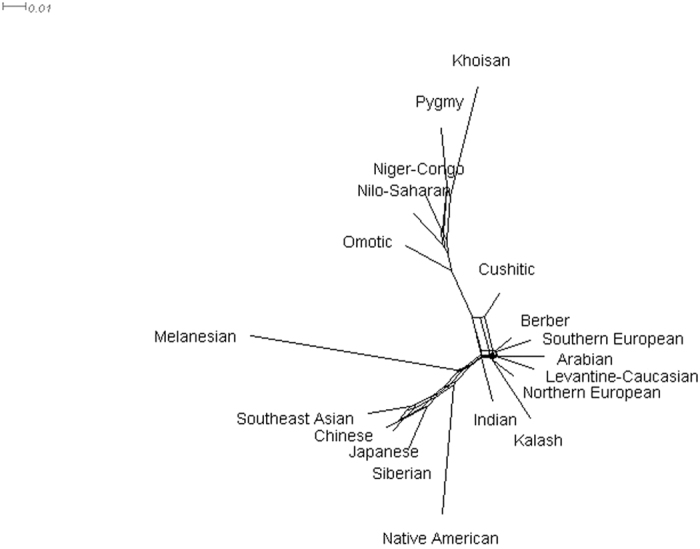
Split decomposition network of ancestries. The underlying distance matrix is based on pairwise *F*_*ST*_.

We previously analyzed autosomal genotype from 3,528 individuals (Supplementary Table S1) and identified 19 ancestries^3^. Phylogenetic trees based on these ancestries assume a strictly bifurcating process. To investigate this assumption, we first employed split decomposition analysis of the distance matrix based on pairwise *F*_*ST*_ estimates between ancestries. This analysis revealed network-like behavior, with Cushitic ancestry appearing the most non-tree-like (Fig. 1). As measured by the Q-residual score, Cushitic ancestry indeed contributed the most to network-like behavior (Table 1).

Networks can represent splits that are incompatible due to several factors, including misspecification in the evolutionary model (*e.g*., substitution probabilities) and misspecification of the topology (*e.g*., due to gene flow). To further investigate network-like behavior in our data set, we used two approaches based on ancestry-specific allele frequencies. First, we estimated all 2,907 possible combinations of the *f*_3_ statistic^8^. A negative *f*_3_ statistic is consistent with admixture, as well as isolation by distance. Of the 2,907 combinations, 273 were significantly negative after Bonferroni correction (Supplementary Table S2). These results further support the existence of network-like behavior. Of the 273 significant *f*_3_ statistics, 41 were consistent with an admixed origin of Cushitic ancestry, with one parent being one of five sub-Saharan ancestries and the other parent being one of the other 13 ancestries (Table 2).

**Figure 2:**
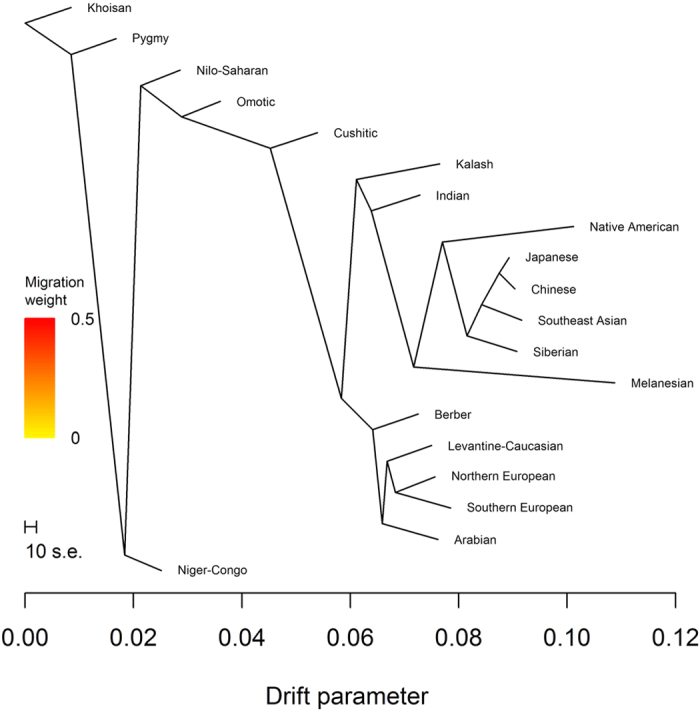
TreeMix analysis with no migration events. The most frequently found tree with no migration events.

**Figure 3:**
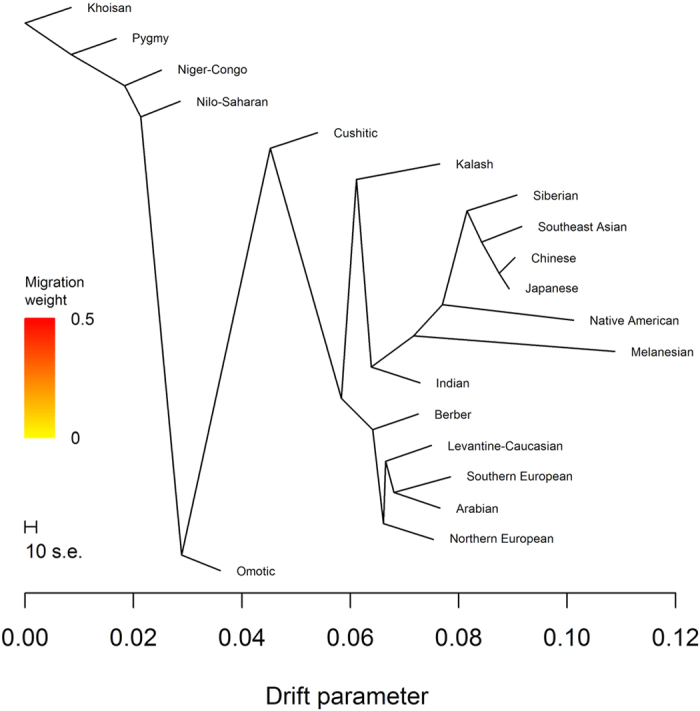
TreeMix analysis with no migration events. The second most frequently found tree with no migration events.

Second, we used the graph-based model implemented in TreeMix^9^ to estimate migration events from the ancestry-specific allele frequencies. The likelihood function in TreeMix is a composite likelihood, not a maximum likelihood, and cannot be used for formal significance testing. In particular, as we increased the number of migration events fit by the model, log-likelihoods became positive, raising a concern of model misspecification or over-fitting. Also, the directions of migration events are not identifiable by the model; directions are assigned by assuming gene flow from the edge with the largest weight, with weights related to mixture proportions. Conditional on no migration events, two trees accounted for 98% of the search space. The tree with the best log-likelihood was found 75% of the time and contained the subtree (Arabian, (Levantine-Caucasian, (Northern European, Southern European))) (Fig. 2). The tree with the second best log-likelihood was found 23% of the time and contained the subtree (Northern European, (Levantine-Caucasian, (Southern European, Arabian))) (Fig. 3).

**Figure 4:**
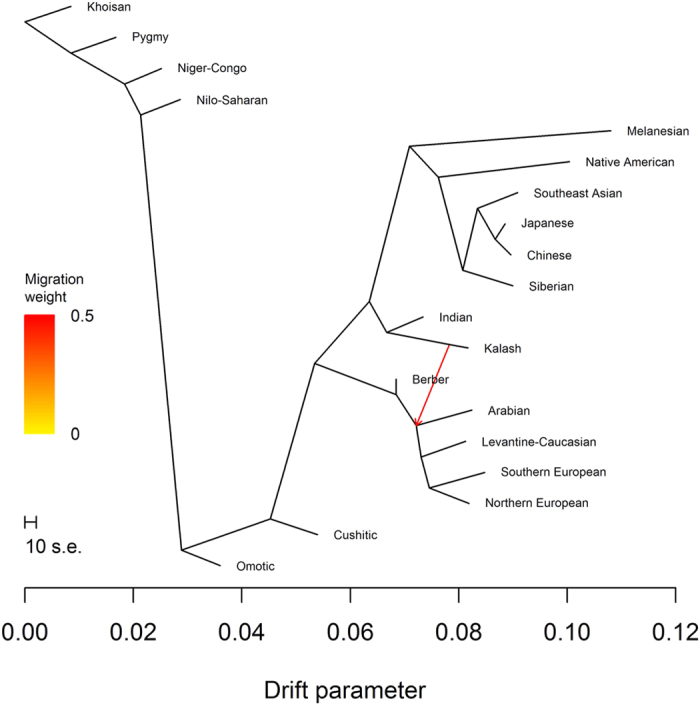
TreeMix analysis with one migration event. The most frequently found tree with one migration event.

**Figure 5:**
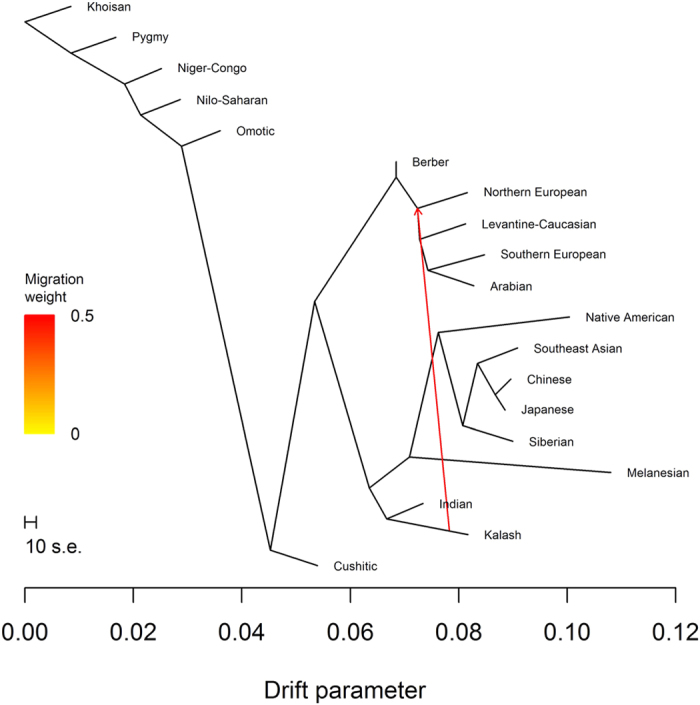
TreeMix analysis with one migration event. The second most frequently found tree with one migration event.

Conditional on one migration event, we observed the tree containing the (Northern European, Southern European) subtree 71% of the time (Fig. 4) and the tree containing the (Southern European, Arabian) subtree 27% of the time (Fig. 5). The same migration event was inferred for both trees: gene flow between Kalash ancestry, after it diverged from Indian ancestry, and the internal node representing the common ancestor of Arabian, Levantine-Caucasian, Northern European, and Southern European ancestries. Four *f*_3_ statistics supported Kalash ancestry as significantly admixed (Supplementary Table S2). Using the *f*_4_ statistics (*Southern European*, *Khoisan*; *Kalash*, *Indian*) and (*Southern European*, *Khoisan*; *Northern European*, *Indian*), we estimated a median mixture proportion of 0.490 (IQR 1.604), indicating that Kalash ancestry is a mixture of 49.0% Northern European and 51.0% Indian ancestries. Similarly, we estimated mixtures of 37.1% Arabian and 62.9% Indian ancestries or 49.4% Levantine-Caucasian and 50.6% Indian ancestries. It is possible that Arabian, Levantine-Caucasian, Northern European, and Southern European ancestries are proxies for one ancestry. However, the distribution of Y DNA haplogroups in the Kalash people consists of 20.5% H and 25.0% L, common in India and South Asia, respectively, mixed with 18.2% G, 9.1% J, and 18.2% R1a, common in the Levant and the Caucasus, the Middle East, and Northern Europe, respectively^10^. Thus, our results based on autosomal data are consistent with Y DNA haplogroup data indicating multi-way admixture in the history of Kalash ancestry.

**Figure 6:**
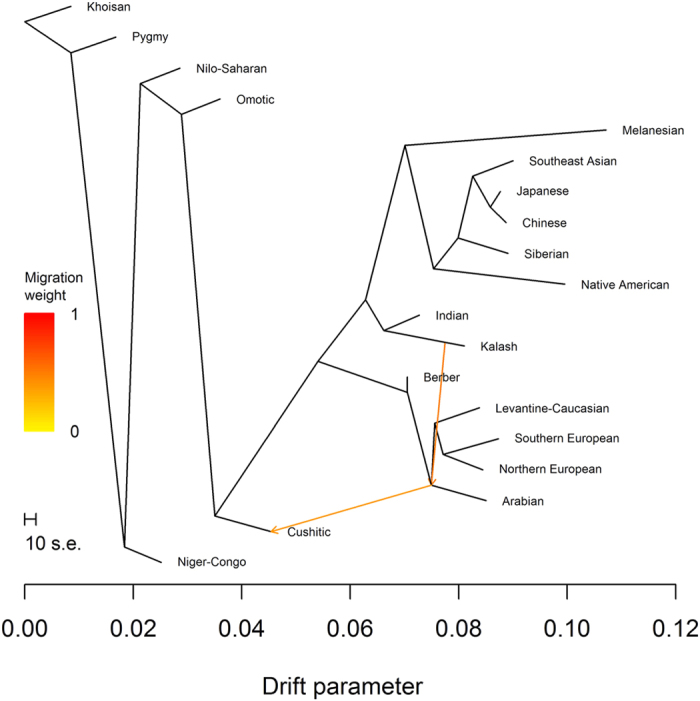
TreeMix analysis with two migration events. The most frequently found tree with two migration events.

**Figure 7:**
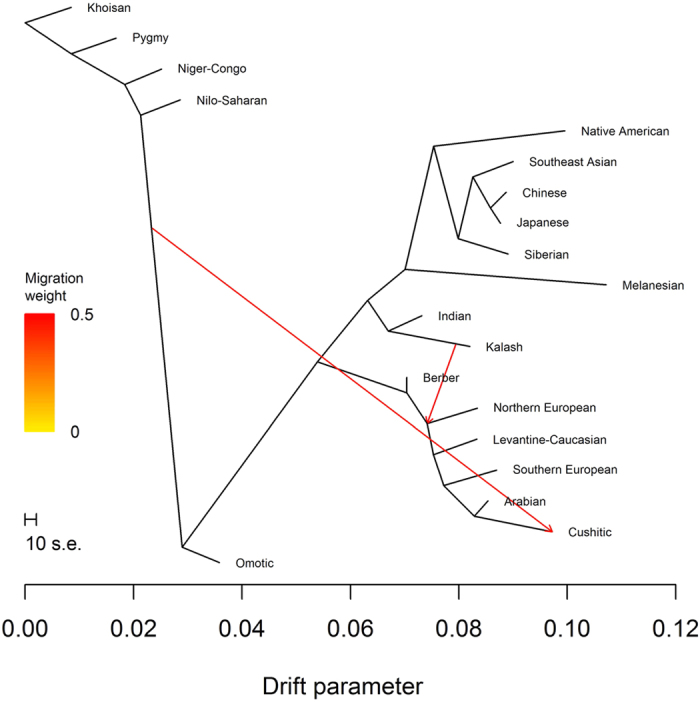
TreeMix analysis with two migration events. The second most frequently found tree with two migration events.

Conditional on two migration events, we observed the tree containing the (Northern European, Southern European) subtree 69% of the time (Fig. 6). The second migration event indicated gene flow between the common ancestor of Arabian, Levantine-Caucasian, Northern European, and Southern European ancestries and Cushitic ancestry, which grouped with Nilo-Saharan and Omotic ancestries. The weights suggested 39.9% ancestry closely related to Arabian ancestry and 60.1% ancestry closely related to Nilo-Saharan and/or Omotic ancestry. We observed the tree containing the (Southern European, Arabian) subtree 30% of the time (Fig. 7). For this tree, Cushitic ancestry no longer grouped with the other sub-Saharan African ancestries but was most closely related to Arabian ancestry, with gene flow from ancestry closely related to Nilo-Saharan and/or Omotic ancestry. The weights suggested 46.0% ancestry closely related to Nilo-Saharan and/or Omotic ancestry and 54.0% ancestry closely related to Arabian ancestry. Weighting these weights by the frequencies of the trees yielded estimates of 55.8% ancestry closely related to Nilo-Saharan and/or Omotic ancestry and 44.2% ancestry closely related to Arabian ancestry. Using the *f*_4_ statistics (*Niger*-*Congo*, *Khoisan*; *Cushitic*, *Arabian*) and (*Niger*-*Congo*, *Khoisan*; *Nilo*-*Saharan*, *Arabian*), we estimated a median mixture proportion of 0.412 (IQR 1.024), indicating 41.2% Nilo-Saharan and 58.8% Arabian ancestry. The *f*_4_ statistics (*Niger*-*Congo*, *Khoisan*; *Cushitic*, *Arabian*) and (*Niger*-*Congo*, *Khoisan*; *Omotic*, *Arabian*) yielded 41.7% Omotic ancestry and 58.3% Arabian ancestry, suggesting that Nilo-Saharan and Omotic ancestries are nearly equally good proxies for one parent of Cushitic ancestry.

**Figure 8:**
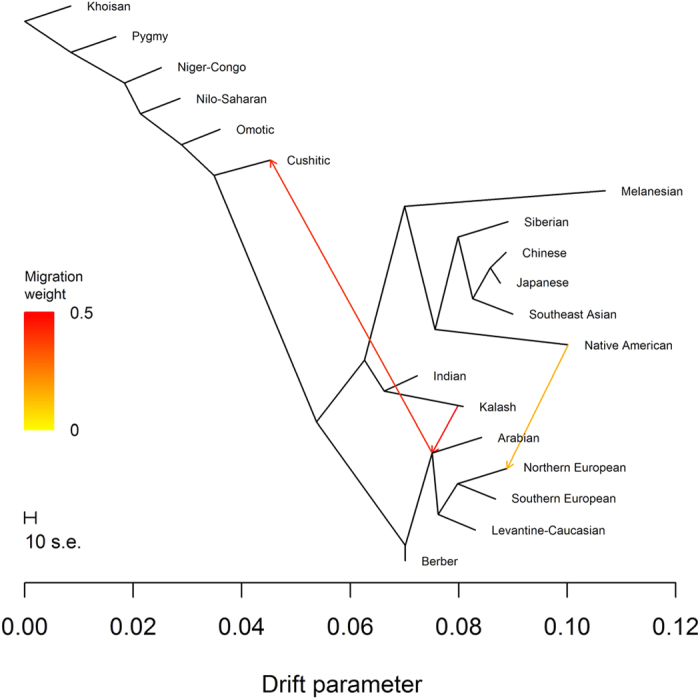
TreeMix analysis with three migration events. The most frequently found tree with three migration events.

Conditional on three migration events, we observed the tree containing the (Northern European, Southern European) subtree 71% of the time (Fig. 8). The third migration event indicated gene flow involving 15.5% Native American ancestry and 84.5% Northern European ancestry. The migration edge connected the tips of the Native American and Northern European branches, implying an event more recent than the divergence of Y DNA haplogroups Q and R. Using (*Levantine*-*Caucasian*, *Khoisan*; *Northern European*, *Native American*) and (*Levantine*-*Caucasian*, *Khoisan*; *Southern European*, *Native American*), we estimated a median mixture proportion of 0.776 (IQR 0.916), indicating 22.4% Native American ancestry. Four *f*_3_ statistics supported Northern European ancestry as significantly admixed (Supplementary Table S2). The remaining trees showed no well-supported topologies and were inconsistent with the first two migration events so we do not discuss them further.

**Figure 9:**
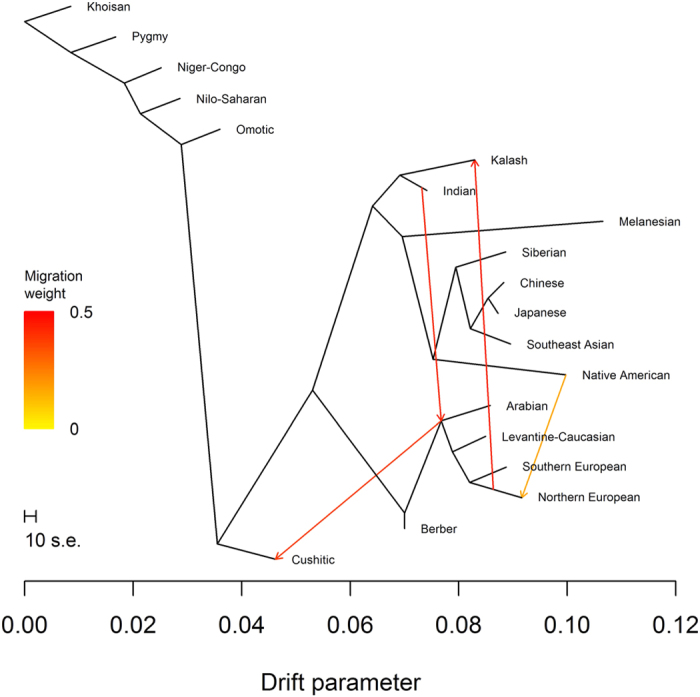
TreeMix analysis with four migration events. The most frequently found tree with four migration events.

Conditional on four migration events, we observed the tree containing the (Northern European, Southern European) subtree 79% of the time (Fig. 9). The fourth migration event indicated a mixture involving 42.1% Indian ancestry and 57.9% of the common ancestor of Arabian, Levantine-Caucasian, Northern European, and Southern European ancestries. Using (*Southern European*, *Khoisan*; *Arabian*, *Indian*) and (*Southern European*, *Khoisan*; *Levantine*-*Caucasian*, *Indian*), we estimated a median mixture proportion of 0.728 (IQR 1.582), indicating 27.2% Indian ancestry. It is possible that the second and fourth migration events are part of the same global-scale event, connecting India and East Africa. The migration event involving Kalash ancestry was also redefined to indicate specifically a mixture involving Northern European ancestry.

Starting with five migration events, we observed positive log-likelihoods and the range of residuals stopped decreasing (Supplementary Figures S1 and S2). Despite low confidence, we report three additional migration events that may be real: the fifth migration event connects sub-Saharan Africa to an internal node ancestral to Chinese, Japanese, Melanesian, Native American, Siberian, and Southeast Asian ancestries; the sixth migration event connects Siberian and Northern European ancestries; and the seventh migration event connects the Kalash and Levantine-Caucasian ancestries (Supplementary Figure S2).

## Discussion

Analyses of ancestries of modern humans typically assume a divergence under isolation model. Here, we performed analyses of human ancestry explicitly allowing for gene flow. We found evidence for four migration events, including one that reconciles the origin of Cushitic ancestry that has been the subject of debate in the literature.

Topologically, the distributions of graphs found by TreeMix were not unimodal. In the primary mode, Northern and Southern European ancestries were siblings, consistent with the distribution and genealogical relationship of Y DNA haplogroups R1a and R1b, respectively^11^. In the secondary mode, Southern European and Arabian ancestries were siblings, consistent with the distribution and genealogical relationship of Y DNA haplogroups I2 and J, respectively^11^.

The first migration event we detected is consistent with the distribution of Y DNA haplogroups in the Kalash people: H and L, common in India and South Asia, respectively, mixed with G, J, and R1a, common in the Levant and the Caucasus, the Middle East, and Northern Europe, respectively^10^. The mixture of Indian/South Asian, Levantine-Caucasian, and Northern European ancestries in the Kalash people has also been observed in several neighboring peoples, including the Baloch, Brahui, Burusho, Makrani, Pashtun, and Sindhi peoples in Pakistan^3^. Ayub *et al*.^12^ reported evidence of admixture approximately 100 generations ago between people with ancestry similar to the Chamars, the Kol, or Gujarati and people with ancestry similar to Armenians or Adygei. The presence of five Y DNA haplogroups in the Kalash people indicates at least four mixing events. The fourth migration event also redefined the first event to involve Kalash and Northern European ancestries, whereas the seventh event involved Levantine-Caucasian ancestry, thus accounting for two of the mixing events.

The second migration event indicates that Cushitic ancestry is a mixture of ancestries closely related to Nilo-Saharan/Omotic and Arabian ancestries. This migration event resolves the observed unstable placement between East African and Middle Eastern ancestries^3^,^4^,^6^: Cushitic ancestry has both East African and Middle Eastern origins. The fourth migration event indicated gene flow between Indian ancestry and the common ancestor of Arabian, Levantine-Caucasian, Northern European, and Southern European ancestries. In conjunction with the second migration event, this event connects India to East Africa. Taken together, these two events are consistent with the presence among East Africans of the mitochondrial DNA haplogroup M1, possibly reflecting migration from India to East Africa following the Last Glacial Maximum^13^,^14^,^15^. In the Nilotic Maasai, the presence of 50% Y DNA haplogroup E1b1b and the absence of Y DNA haplogroups J and T^16^ suggest a depletion of male Arabian ancestry, raising the possibility that Cushitic ancestry formed as a mixture of male-biased Nilo-Saharan/Omotic ancestry and female-biased Arabian ancestry.

To interpret the third migration event, we note that the main Y DNA haplogroups consistent with Native American ancestry (detected as the major ancestry in Karitiana, Surui, Pima, Colombian, Maya, and MXL samples^3^) and Northern European ancestry (detected as the major ancestry in FIN, Lithuania, Russian, and Belorussia samples^3^) are Q^17^ and R1a^11^, respectively, which are siblings descended from haplogroup P. Given this relationship, the observation that Native American and Northern European ancestries do not group together suggests that more shared autosomal ancestry is female-derived rather than male-derived and that the migration event therefore represents male-biased gene flow. This event is unlikely to reflect migration of Yamnaya steppe herders during the Bronze Age because Yamnaya males are associated predominantly with Y DNA haplogroup R1b and, to a lesser extent, I2a^18^,^19^,^20^. Also, this event does not reflect migration of Siberians into Northeast Europe^21^, which is captured by the sixth migration event. Siberian ancestry, detected as the major ancestry in a sample of Yakut people, is distinct from both Native American and Northern European ancestries^3^ and is associated with Y DNA haplogroup N^22^. In contrast, this event is consistent with a low frequency of Y DNA haplogroup Q observed in Romania and Hungary^23^ and Bulgaria^24^. Given that the migration edge connected the external nodes of both ancestries, we hypothesize that the event was relatively recent and therefore involved Central or North Asians rather than Native Americans.

TreeMix potentially inferred migration involving sub-Saharan and Melanesian ancestries as the fifth migration event. One edge of this event was placed between Khoisan/Pygmy ancestries and Niger-Congo/Nilo-Saharan ancestries. Based on distributions of Y DNA haplogroups, this event falls between haplogroups A/B and E^25^. Y DNA haplogroup C is presently found in populations in Japan, Oceania, Mongolia and Siberia, Australia, and India^26^. Y DNA haplogroup D is presently found in Japan, Tibet, and the Andaman Islands. Thus, this event might be capturing elements of Y DNA haplogroups C and/or D remaining from the Out of Africa migrations. It is therefore possible that the long branch we previously observed for Melanesian ancestry resulted from gene flow of K haplogroups (and/or its descendants M, O, and S) into a pre-existing population with predominantly haplogroup C^27^.

In light of these findings, we re-evaluated ancestral divergence times^3^, excluding Cushitic, Kalash, and Melanesian ancestries. In one subtree, a divergence event occurred approximately 72,800 years ago between the ancestor of Khoisan and Pygmy ancestries and the ancestor of Omotic, Niger-Congo, and Nilo-Saharan ancestries. A second divergence event occurred approximately 71,500 years ago between the ancestor of Native American, Siberian, Southeast Asian, Japanese, and Chinese ancestries and the ancestor of Indian, Kalash, Arabian, Berber, Levantine-Caucasian, Northern European, and Southern European ancestries. These times are consistent with migration out of Africa involving Y DNA haplogroup DE followed by a second migration involving haplogroup CF^28^. Temporally, both of these events coincide with the beginning of marine isotope stage 4, a 1,000-year stadial or cold glacial period^29^.

Our current findings provide genetic evidence for four migration events in the distant past without requiring ancient DNA, although the absence of genotype data for ancestries limits our ability to make inferences, particularly about dates. Additionally, comparison of autosomal data with distributions of Y and mitochondrial DNA haplogroups suggests sex-biased gene flow. Taken together, analyses based on ancestries rather than present-day samples provide new insights into the migratory history of modern humans in the distant past.

## Methods

We performed collection and quality control of genome-wide genotype data from 3,528 unrelated individuals from 163 global samples (Supplementary Table S1), as previously described^3^. These data are freely available at http://crggh.nih.gov/resources.cfm under Ancestry. Unsupervised ancestry analysis was performed using ADMIXTURE^30^, with the optimal value of the number of ancestral components *K* determined to be 19 by five-fold cross-validation, averaged over three runs with different starting seeds.

We used SplitsTree version 4.13.1^7^ to perform split decomposition analysis. This analysis utilized the distance matrix based on pairwise *F*_*ST*_ between ancestries as reported by ADMIXTURE. We used the Q-residual score to assess deviation from tree-like behavior^31^.

We calculated the *f*_3_ statistic per marker as described in the Supplementary Text. We also derived the theoretical variance of the *f*_3_ statistic (Supplementary Text). Using unbiased means and variances, we calculated marker-specific *z*-scores, which we then combined using equal weighting into a single *z*-score (Supplementary Text). We tested all 2,907 possible combinations of the 19 ancestries, using the allele frequencies reported by ADMIXTURE. For each ancestry, the sum of the estimated proportions for each individual is an estimate of the effective sample size; hence, twice this sum is an estimate of the total allele count.

Using *f*_4_ statistics^8^,^32^, the mixture proportion can be estimated using the ratio 
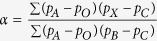
, with allele frequencies *p*_*X*_, *p*_*C*_, *p*_*B*_, *p*_*A*_, and *p*_*O*_ in the putatively admixed ancestry *X*, proxies *C* and *B* of the putative parental ancestries, an ancestry *A* that is a sibling to ancestry *B*, and outgroup ancestry *O*, respectively. Both summations are over all markers. The ratio *α* is the mixture proportion of *B* and 1 − *α* is the mixture proportion of *C*. The *f*_4_ statistic follows the normal product distribution. Since the *f*_4_ statistic has finite mean and variance, both the numerator and denominator sums are normally distributed, by the Central Limit Theorem. Hence, *α* is a ratio of dependent normal variables. The distribution of the ratio of dependent normal variables follows a Cauchy-like distribution, which does not have finite moments^33^. Consequently, the sample estimator described by Reich *et al*.^8^ and Patterson *et al*.^32^ is inconsistent, as the sample mean is undefined and the sample variance is infinite. Instead, we calculated the ratio per marker and reported sample medians and interquartile ranges across markers. For a Cauchy distribution, the sample median is an estimate of the location parameter and the interquartile range is twice the scale parameter. Note that 
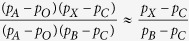
, with equality if *p*_*A*_ − *p*_*O*_ ≠ 0, in which the right-hand side is the estimator of the mixing proportion assuming *αp*_*B*_ + (1 − *α*)*p*_*C*_ = *p*_*X*_ (*i.e*., *A* and *O* are unnecessary).

We converted ADMIXTURE output for TreeMix^9^ by rounding the allele counts to the nearest integer, using the allele frequencies and total allele counts as described above. We assigned Khoisan ancestry as the root. We set the number of migration events from 0 to 7. For each number of migration events, we ran 100 random input orders.

### Ethics

This project was determined to be excluded from IRB Review by the National Institutes of Health Office of Human Subjects Research Protections, Protocol #12183.

## Additional Information

**How to cite this article**: Shriner, D. *et al*. Ancient Human Migration after Out-of-Africa. *Sci. Rep*. **6**, 26565; doi: 10.1038/srep26565 (2016).

## Supplementary Material

Supplementary Information

## Figures and Tables

**Table 1:** Non-tree-like behavior among ancestries.

Excluded Ancestry	Q-residual score
Cushitic	0.0073
Berber	0.0084
Northern European	0.0095
Arabian	0.0096
Southern European	0.0098
Levantine-Caucasian	0.0100
Indian	0.0103
Kalash	0.0103
Southeast Asian	0.0104
Niger-Congo	0.0105
Nilo-Saharan	0.0106
Japanese	0.0106
Chinese	0.0106
Siberian	0.0107
Native American	0.0108
Omotic	0.0108
Melanesian	0.0110
Pygmy	0.0110
Khoisan	0.0115

The Q-residual score measures deviation from tree-like behavior. The graph including all 19 ancestries had a Q-residual score of 0.0102. We excluded each ancestry one at a time and recalculated the Q-residual score. A lower score indicates that the excluded ancestry contributed more to non-tree-like behavior, as the remaining ancestries are more tree-like. Thus, Cushitic ancestry contributed the most to non-tree-like behavior.

**Table 2:** *f*_3_ statistics testing for admixture in Cushitic ancestry.

Parent Ancestry *A*	Parent Ancestry *B*	*Z*-score	*P*-value
Niger-Congo	Southern European	−124.39	0
Khoisan	Southern European	−102.48	0
Niger-Congo	Arabian	−97.76	0
Nilo-Saharan	Southern European	−97.58	0
Niger-Congo	Levantine-Caucasian	−95.69	0
Khoisan	Levantine-Caucasian	−83.61	0
Pygmy	Southern European	−80.98	0
Khoisan	Arabian	−77.93	0
Niger-Congo	Northern European	−77.75	0
Nilo-Saharan	Arabian	−74.33	0
Khoisan	Northern European	−71.98	0
Nilo-Saharan	Levantine-Caucasian	−69.53	0
Pygmy	Arabian	−61.39	0
Pygmy	Levantine-Caucasian	−60.49	0
Nilo-Saharan	Northern European	−57.05	0
Pygmy	Northern European	−49.18	0
Niger-Congo	Native American	−33.52	1.04 × 10^−246^
Niger-Congo	Kalash	−33.11	1.23 × 10^−240^
Khoisan	Native American	−28.86	1.89 × 10^−183^
Khoisan	Chinese	−28.02	5.18 × 10^−173^
Khoisan	Berber	−25.09	3.45 × 10^−139^
Niger-Congo	Melanesian	**−**24.60	6.96 × 10^−134^
Khoisan	Kalash	**−**22.81	1.80 × 10^−115^
Niger-Congo	Chinese	**−**21.20	4.72 × 10^−100^
Omotic	Southern European	**−**19.20	1.89 × 10^−82^
Nilo-Saharan	Kalash	**−**17.24	6.38 × 10^−67^
Nilo-Saharan	Native American	**−**15.08	1.11 × 10^−51^
Niger-Congo	Siberian	−13.19	4.86 × 10^−40^
Pygmy	Kalash	**−**9.72	1.27 × 10^−22^
Khoisan	Japanese	**−**9.67	2.02 × 10^−22^
Pygmy	Berber	**−**9.46	1.53 × 10^−21^
Niger-Congo	Berber	**−**8.91	2.57 × 10^−19^
Khoisan	Southeast Asian	**−**8.63	3.05 × 10^−18^
Niger-Congo	Japanese	**−**8.28	5.92 × 10^−17^
Omotic	Arabian	**−**8.17	1.52 × 10^−16^
Khoisan	Indian	**−**7.85	2.08 × 10^−15^
Khoisan	Siberian	**−**7.72	5.97 × 10^−15^
Nilo-Saharan	Berber	**−**6.65	1.47 × 10^−11^
Niger-Congo	Southeast Asian	**−**6.62	1.82 × 10^−11^
Pygmy	Native American	**−**6.21	2.73 × 10^−10^
Nilo-Saharan	Melanesian	**−**4.44	4.46 × 10^−6^

## References

[b1] Cruciani F. . A revised root for the human Y chromosomal phylogenetic tree: the origin of patrilineal diversity in Africa. Am. J. Hum. Genet. 88, 814–818 (2011). 21601174 10.1016/j.ajhg.2011.05.002 PMC3113241

[b2] Poznik G. D. . Sequencing Y chromosomes resolves discrepancy in time to common ancestor of males versus females. Science 341, 562–565 (2013). 23908239 10.1126/science.1237619 PMC4032117

[b3] Shriner D., Tekola-Ayele F., Adeyemo A. & Rotimi C. N. Genome-wide genotype and sequence-based reconstruction of the 140,000 year history of modern human ancestry. Sci. Rep. 4, 6055 (2014). 25116736 10.1038/srep06055 PMC4131216

[b4] Tishkoff S. A. . The genetic structure and history of Africans and African Americans. Science 324, 1035–1044 (2009). 19407144 10.1126/science.1172257 PMC2947357

[b5] Li J. Z. . Worldwide human relationships inferred from genome-wide patterns of variation. Science 319, 1100–1104 (2008). 18292342 10.1126/science.1153717

[b6] Hodgson J. A., Mulligan C. J., Al-Meeri A. & Raaum R. L. Early back-to-Africa migration into the Horn of Africa. PLoS Genet. 10, e1004393 (2014). 24921250 10.1371/journal.pgen.1004393 PMC4055572

[b7] Huson D. H. & Bryant D. Application of phylogenetic networks in evolutionary studies. Mol. Biol. Evol. 23, 254–267 (2006). 16221896 10.1093/molbev/msj030

[b8] Reich D., Thangaraj K., Patterson N., Price A. L. & Singh L. Reconstructing Indian population history. Nature 461, 489–494 (2009). 19779445 10.1038/nature08365 PMC2842210

[b9] Pickrell J. K. & Pritchard J. K. Inference of population splits and mixtures from genome-wide allele frequency data. PLoS Genet. 8, e1002967 (2012). 23166502 10.1371/journal.pgen.1002967 PMC3499260

[b10] Firasat S. . Y-chromosomal evidence for a limited Greek contribution to the Pathan population of Pakistan. Eur. J. Hum. Genet. 15, 121–126 (2007). 17047675 10.1038/sj.ejhg.5201726 PMC2588664

[b11] Wikipedia. Y-*DNA haplogroups in European populations*. (2015) Available at: https://en.wikipedia.org/wiki/Y-DNA_haplogroups_in_European_populations. Accessed 12 August 2015.

[b12] Ayub Q. . The Kalash genetic isolate: ancient divergence, drift, and selection. Am. J. Hum. Genet. 96, 775–783 (2015). 25937445 10.1016/j.ajhg.2015.03.012 PMC4570283

[b13] Maca-Meyer N., González A. M., Larruga J. M., Flores C. & Cabrera V. M. Major genomic mitochondrial lineages delineate early human expansions. BMC Genet. 2, 13 (2001). 11553319 10.1186/1471-2156-2-13 PMC55343

[b14] González A. M. . Mitochondrial lineage M1 traces an early human backflow to Africa. BMC Genomics 8, 223 (2007). 17620140 10.1186/1471-2164-8-223 PMC1945034

[b15] Clark P. U. . The Last Glacial Maximum. Science 325, 710–714 (2009). 19661421 10.1126/science.1172873

[b16] Wood E. T. . Contrasting patterns of Y chromosome and mtDNA variation in Africa: evidence for sex-biased demographic processes. Eur. J. Hum. Genet. 13, 867–876 (2005). 15856073 10.1038/sj.ejhg.5201408

[b17] Wikipedia. Y-*DNA haplogroups in indigenous peoples of the Americas*. (2015) Available at: https://en.wikipedia.org/wiki/Y-DNA_haplogroups_in_indigenous_peoples_of_the_Americas. Accessed 12 August 2015.

[b18] Allentoft M. E. . Population genomics of Bronze Age Eurasia. Nature 522, 167–172 (2015). 26062507 10.1038/nature14507

[b19] Haak W. . Massive migration from the steppe was a source for Indo-European languages in Europe. Nature 522, 207–211 (2015). 25731166 10.1038/nature14317 PMC5048219

[b20] Mathieson I. . Genome-wide patterns of selection in 230 ancient Eurasians. Nature 528, 499–503, (2015). 26595274 10.1038/nature16152 PMC4918750

[b21] Der Sarkissian C. . Ancient DNA reveals prehistoric gene-flow from Siberia in the complex human population history of North East Europe. PLoS Genet. 9, e1003296 (2013). 23459685 10.1371/journal.pgen.1003296 PMC3573127

[b22] Tambets K. . The western and eastern roots of the Saami–the story of genetic “outliers” told by mitochondrial DNA and Y chromosomes. Am. J. Hum. Genet. 74, 661–682 (2004). 15024688 10.1086/383203 PMC1181943

[b23] Martinez-Cruz B. . Y-chromosome analysis in individuals bearing the Basarab name of the first dynasty of Wallachian kings. PLoS ONE 7, e41803 (2012). 22848614 10.1371/journal.pone.0041803 PMC3404992

[b24] Karachanak S. . Y-chromosome diversity in modern Bulgarians: new clues about their ancestry. PLoS ONE 8, e56779 (2013). 23483890 10.1371/journal.pone.0056779 PMC3590186

[b25] Wikipedia. Y-*DNA haplogroups by populations of Sub-Saharan Africa*. (2015) Available at: https://en.wikipedia.org/wiki/Y-DNA_haplogroups_by_populations_of_Sub-Saharan_Africa. Accessed 12 August 2015.

[b26] Wikipedia. *Haplogroup C-M130*. (2015) Available at: https://en.wikipedia.org/wiki/Haplogroup_C-M130. Accessed 12 August 2015.

[b27] Wikipedia. Y-*DNA haplogroups in Oceanian populations*. (2015) Available at: https://en.wikipedia.org/wiki/Y-DNA_haplogroups_in_Oceanian_populations. Accessed 12 August 2015.

[b28] Underhill P. A. & Kivisild T. Use of Y chromosome and mitochondrial DNA population structure in tracing human migrations. Annu. Rev. Genet. 41, 539–564 (2007). 18076332 10.1146/annurev.genet.41.110306.130407

[b29] Lisiecki L. E. & Raymo M. E. A Pliocene-Pleistocene stack of 57 globally distributed benthic d^18^O records. Paleoceanography 20, PA1003 (2005).

[b30] Alexander D. H., Novembre J. & Lange K. Fast model-based estimation of ancestry in unrelated individuals. Genome Res. 19, 1655–1664 (2009). 19648217 10.1101/gr.094052.109 PMC2752134

[b31] Gray R. D., Bryant D. & Greenhill S. J. On the shape and fabric of human history. Philos. Trans. R. Soc. Lond. B Biol. Sci. 365, 3923–3933 (2010). 21041216 10.1098/rstb.2010.0162 PMC2981918

[b32] Patterson N. . Ancient admixture in human history. Genetics 192, 1065–1093 (2012). 22960212 10.1534/genetics.112.145037 PMC3522152

[b33] Cedilnik A., Košmelj K. & Blejec A. The Distribution of the Ratio of Jointly Normal Variables. Metodološki zvezki 1, 99–108 (2004).

