# Supplementary material for: Ancient Human Migration after Out-of-Africa
Source: Sci Rep. 2016 May 23;6:26565. doi: 10.1038/srep26565 (PMC4876373; doi:10.1038/srep26565)
Supplement: Supplementary Information [file srep26565-s1.pdf]

Ancient Human Migration after Out-of-Africa

Daniel Shriner, Fasil Tekola-Ayele, Adebowale Adeyemo, Charles N. Rotimi

Supplementary Figures S1–S4

Supplementary Text

Supplementary Tables S1 and S2

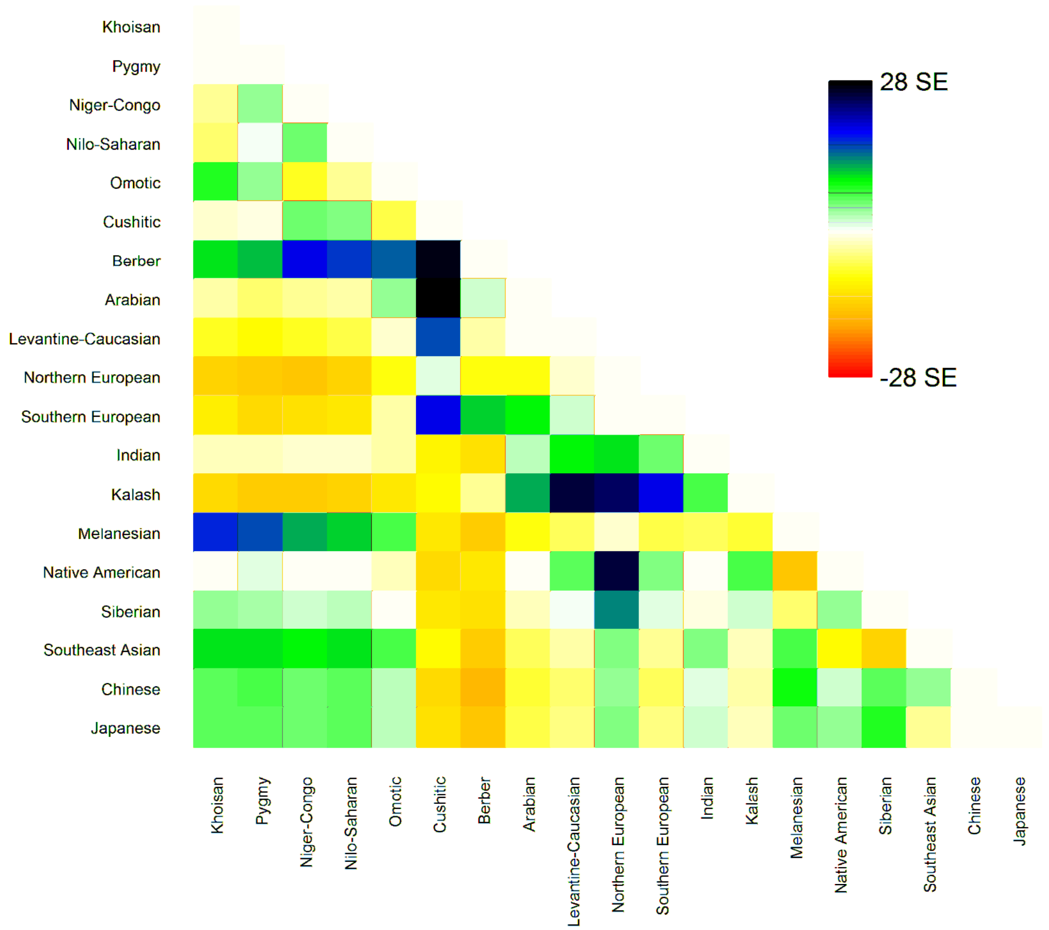

Supplementary Figure S1A. Residual plot corresponding to Figure 2.

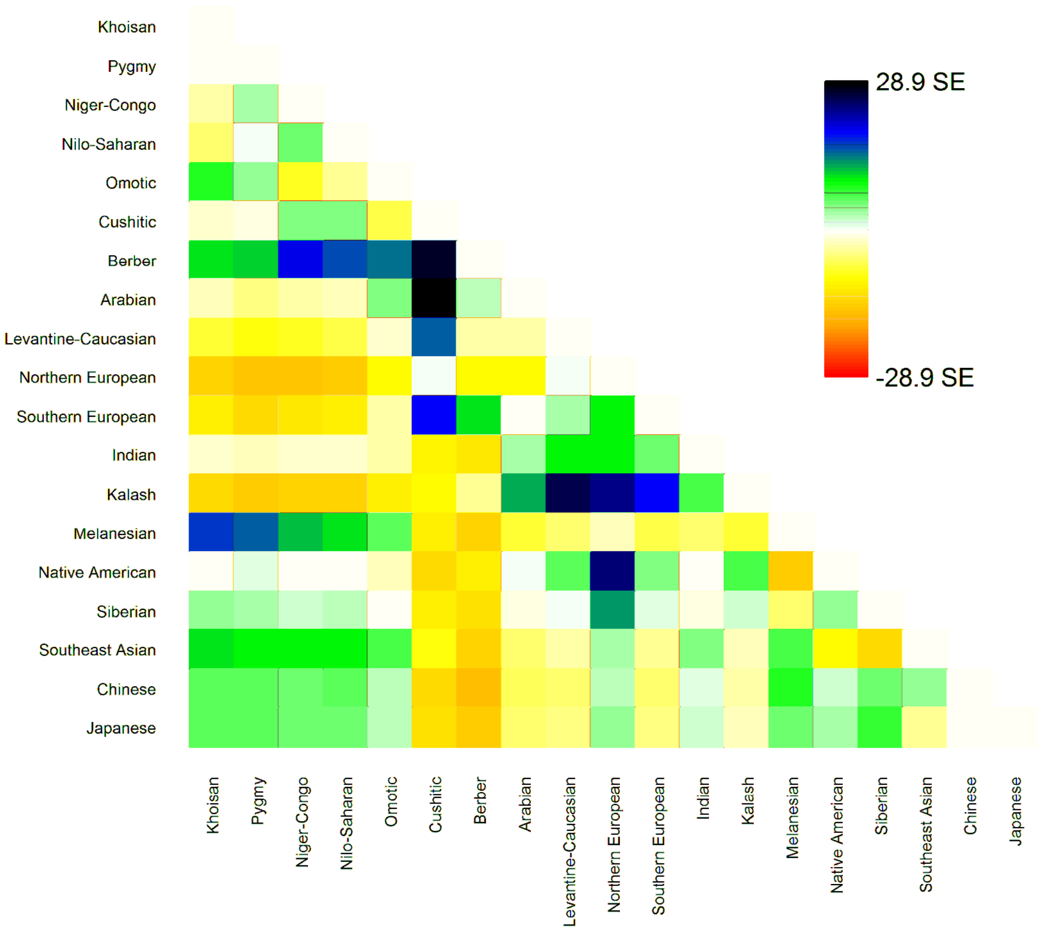

Supplementary Figure S1B. Residual plot corresponding to Figure 3.

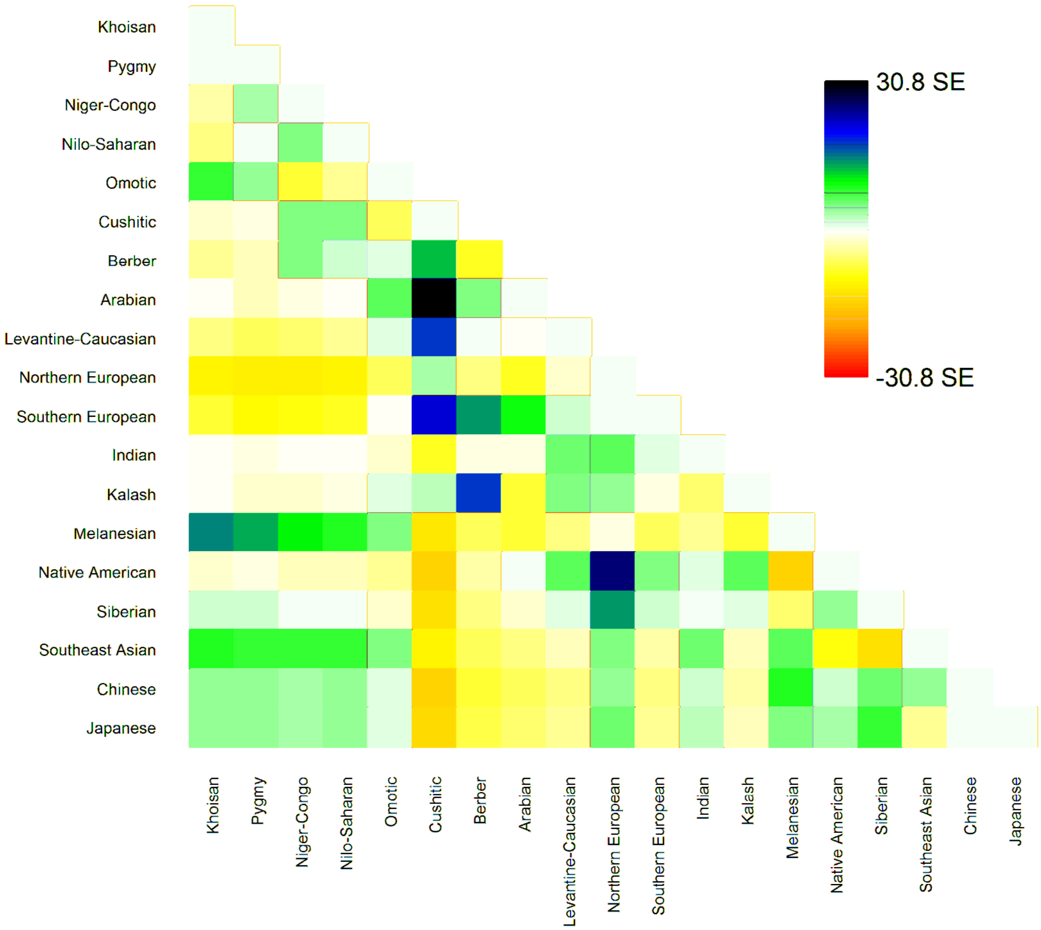

Supplementary Figure S1C. Residual plot corresponding to Figure 4.

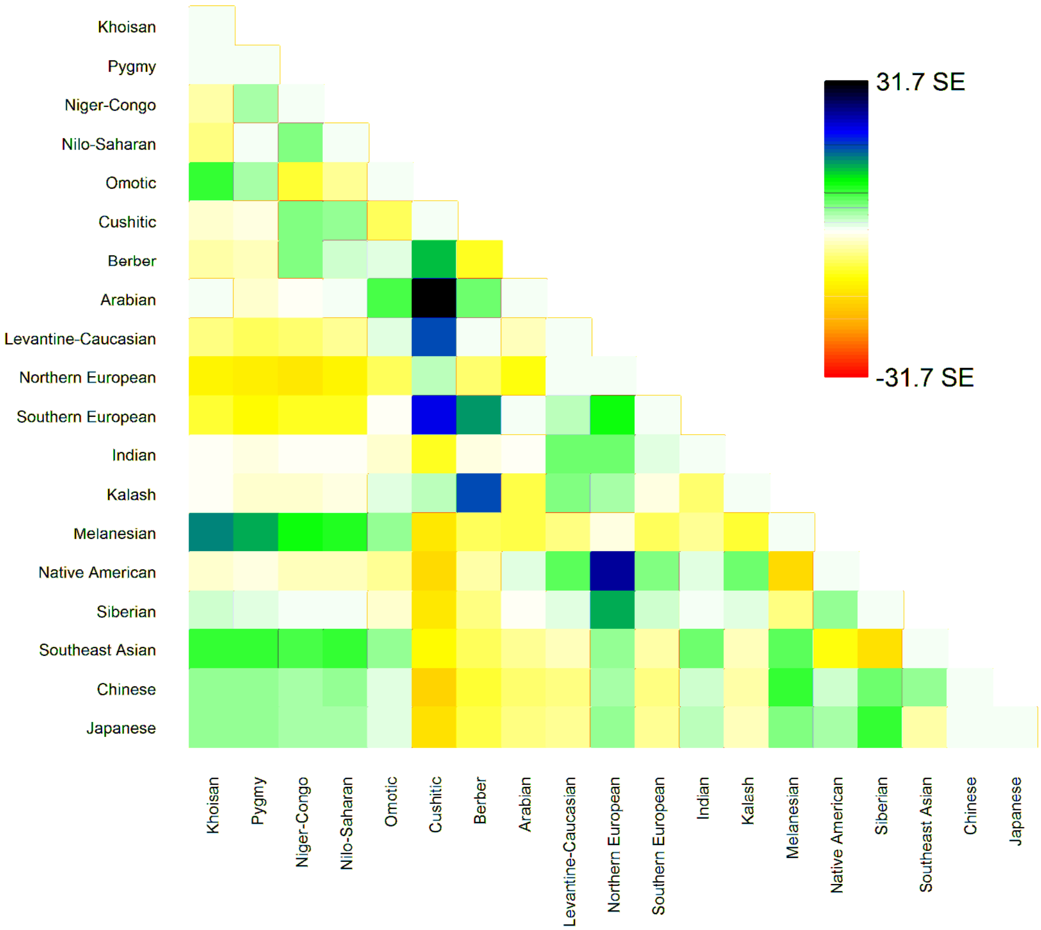

Supplementary Figure S1D. Residual plot corresponding to Figure 5.

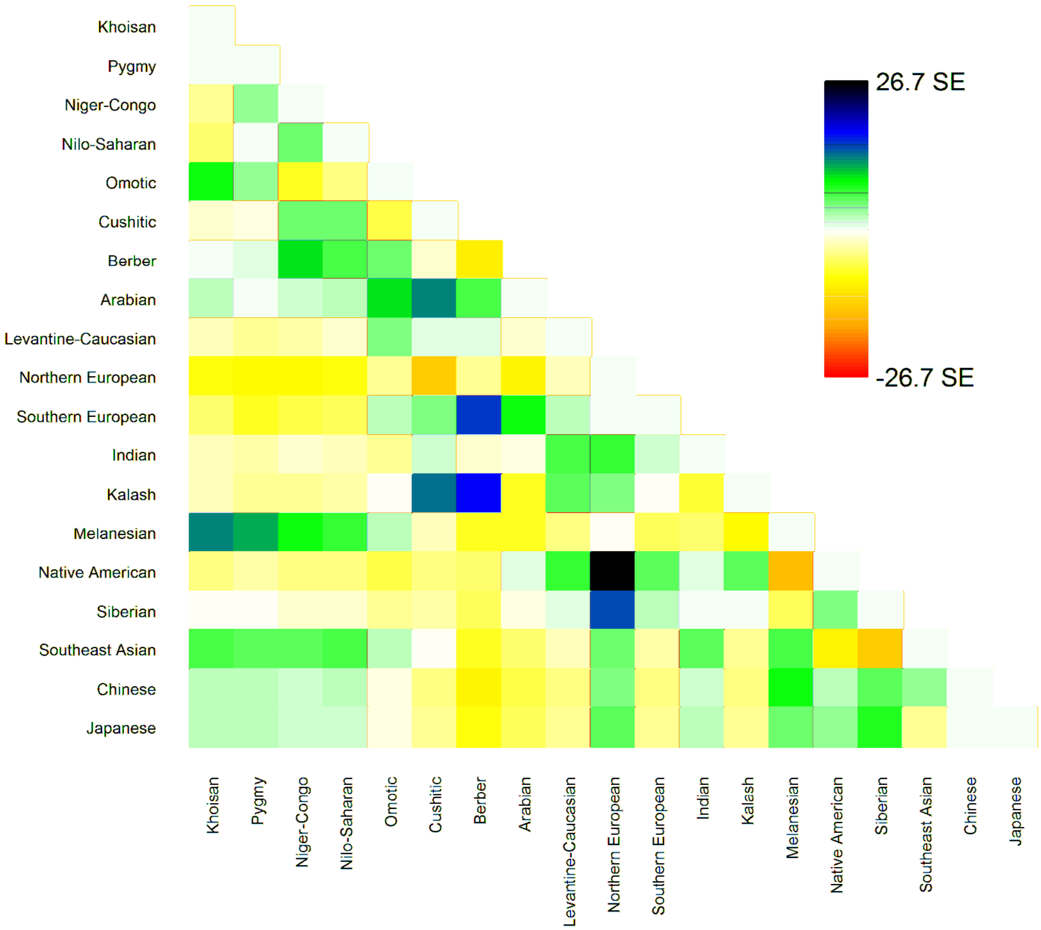

Supplementary Figure S1E. Residual plot corresponding to Figure 6.

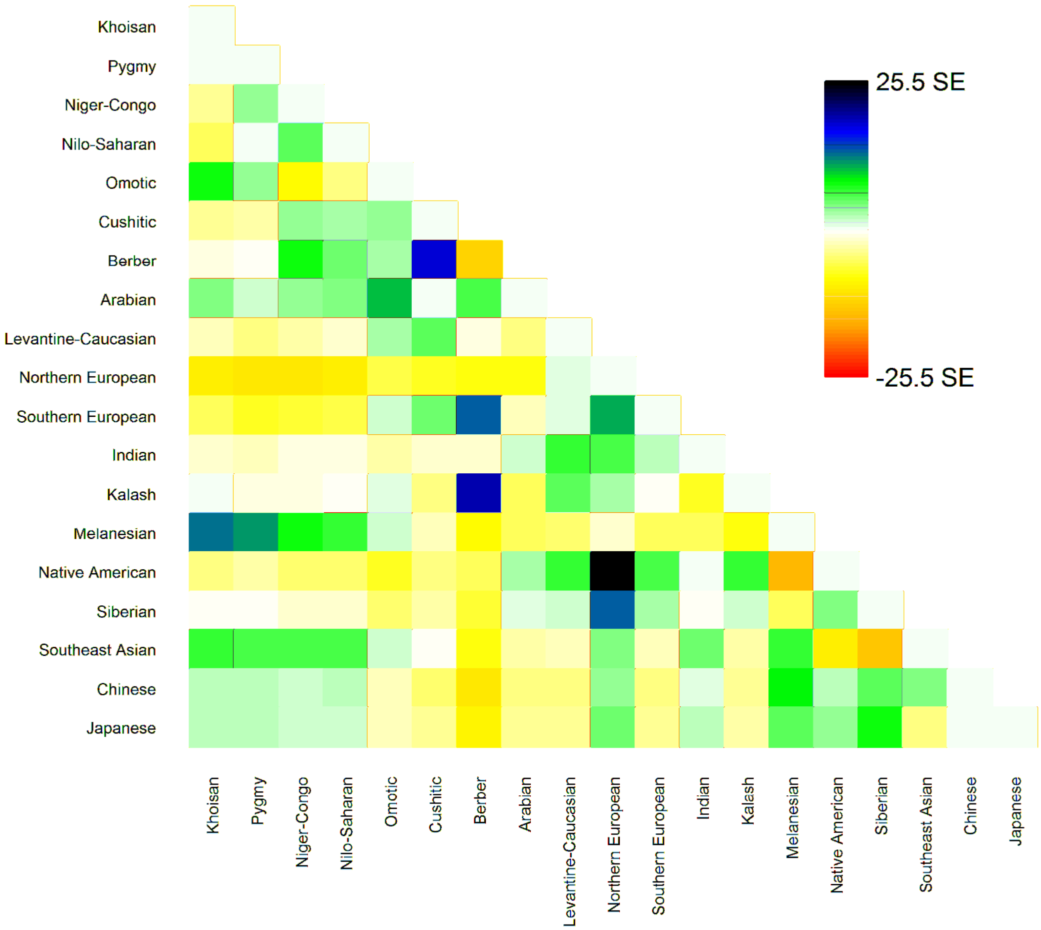

Supplementary Figure S1F. Residual plot corresponding to Figure 7.

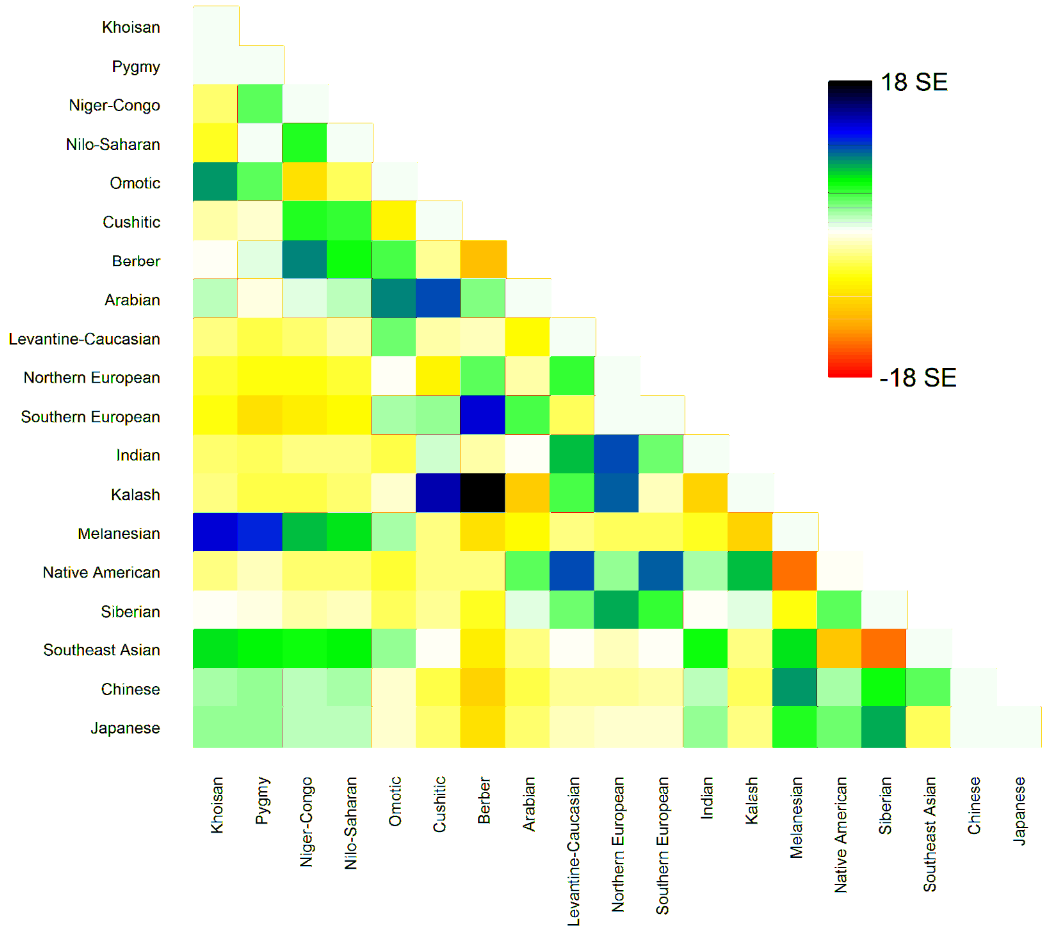

Supplementary Figure S1G. Residual plot corresponding to Figure 8.

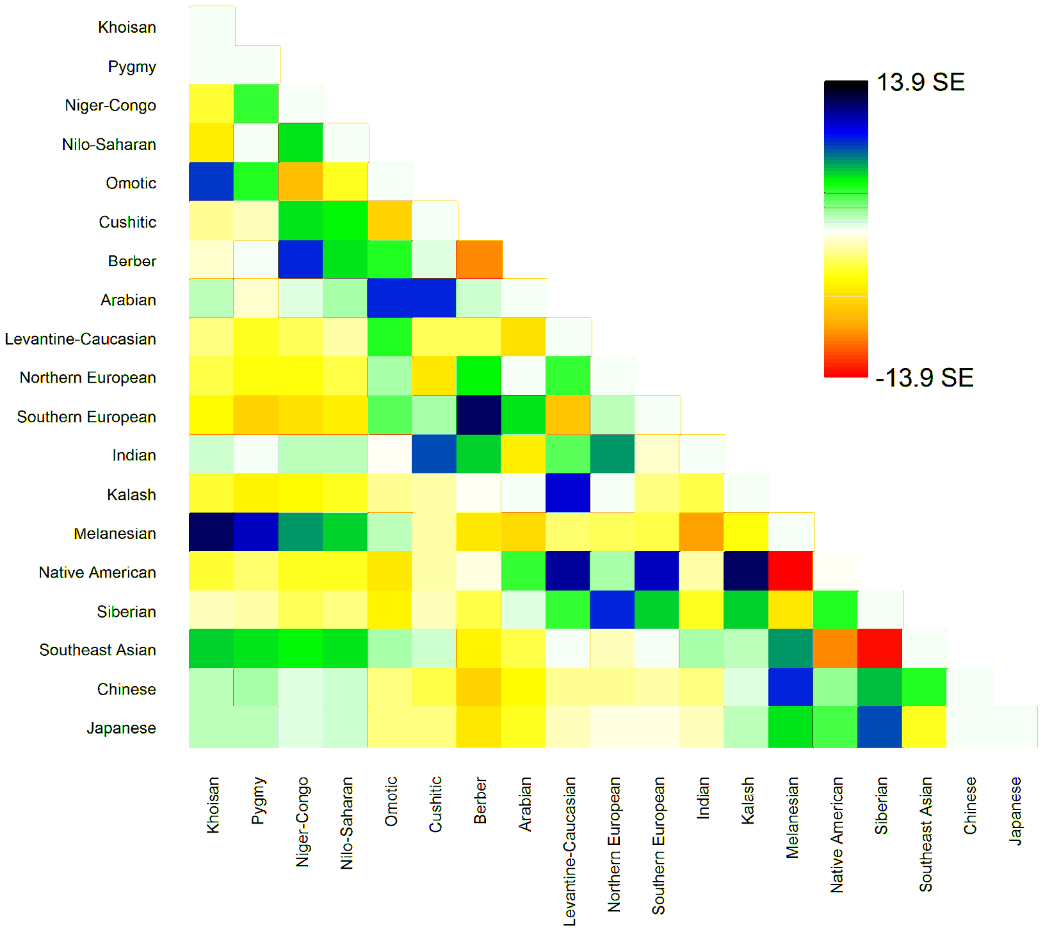

Supplementary Figure S1H. Residual plot corresponding to Figure 9.

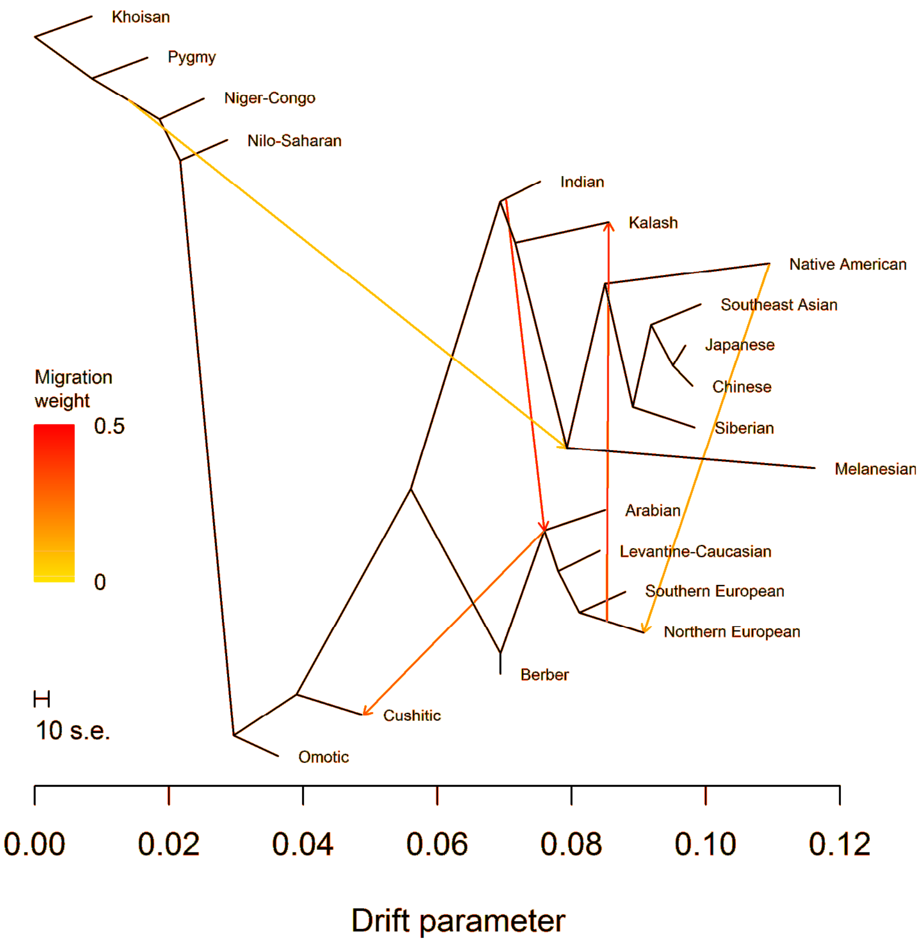

Supplementary Figure S2A. Five migration events. The fifth event suggests gene flow from a point between Khoisan/Pygmy and Niger-Congo/Nilo-Saharan ancestries to East Asia, possibly reflecting Y DNA haplogroups C and/or D.

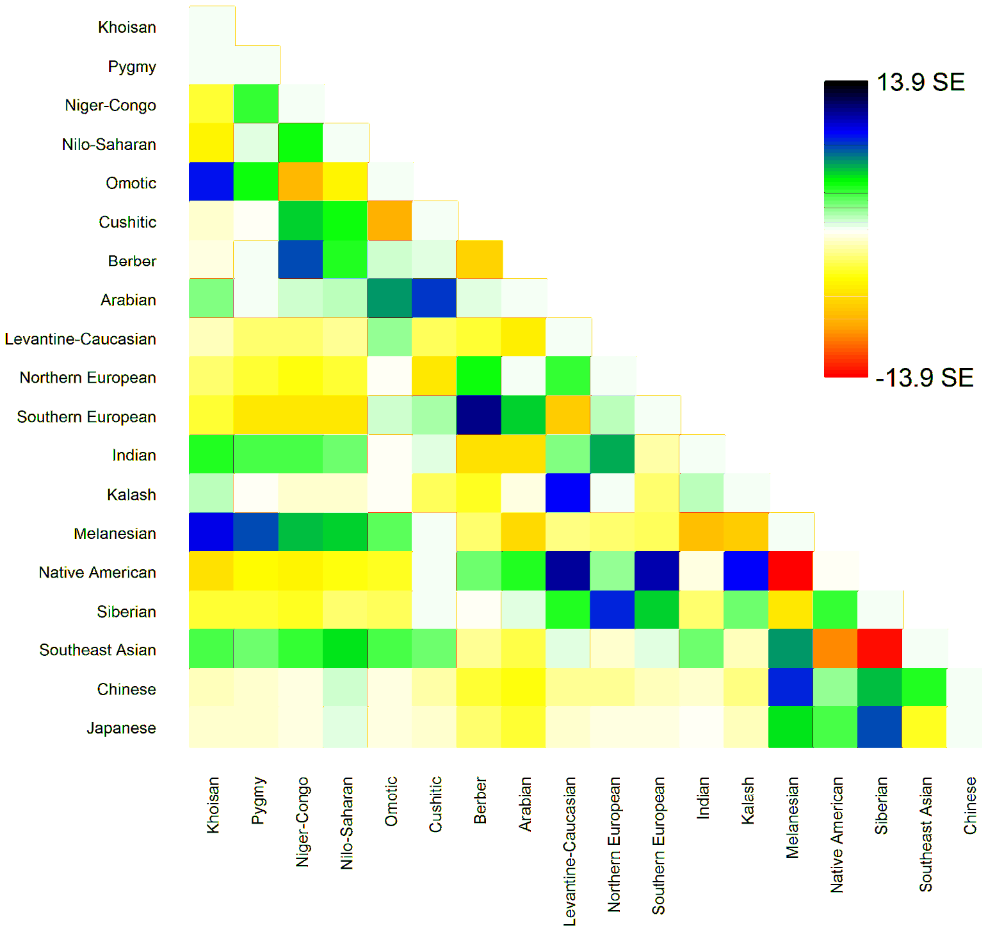

Supplementary Figure S2B. Residual plot corresponding to Supplementary Figure S2A.

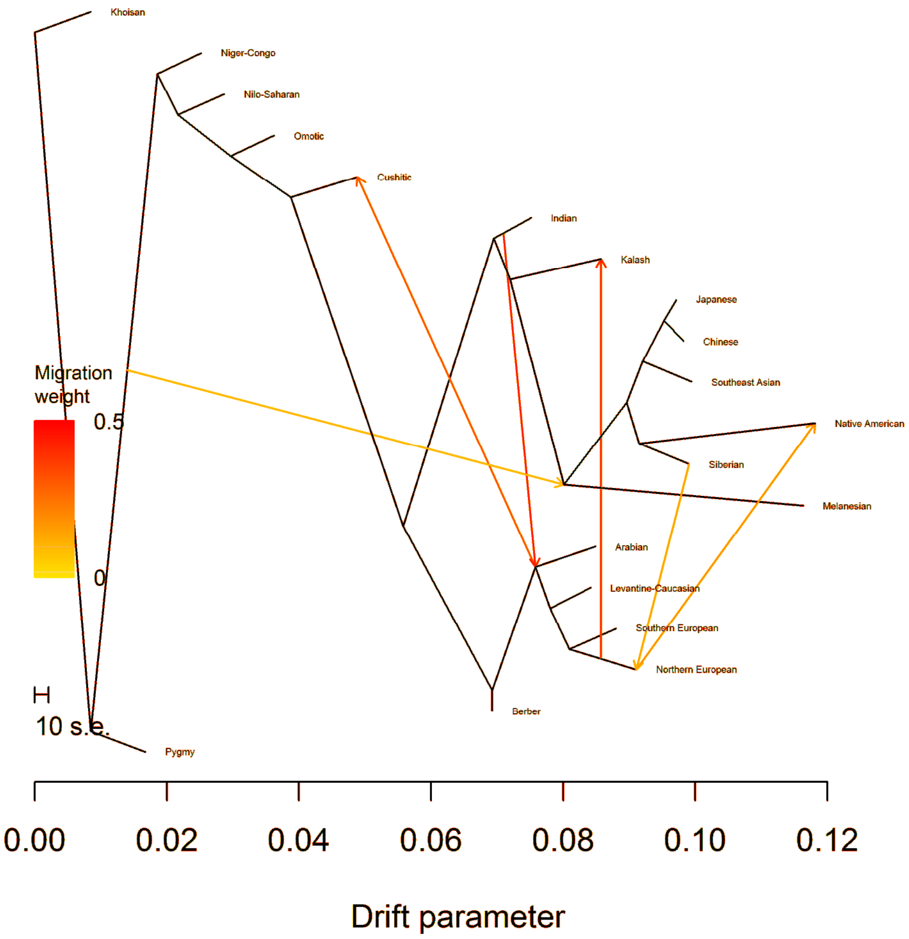

Supplementary Figure S2C. Six migration events. The sixth event suggests gene flow from Siberian to Northern European ancestry, likely reflecting Y DNA haplogroup N.

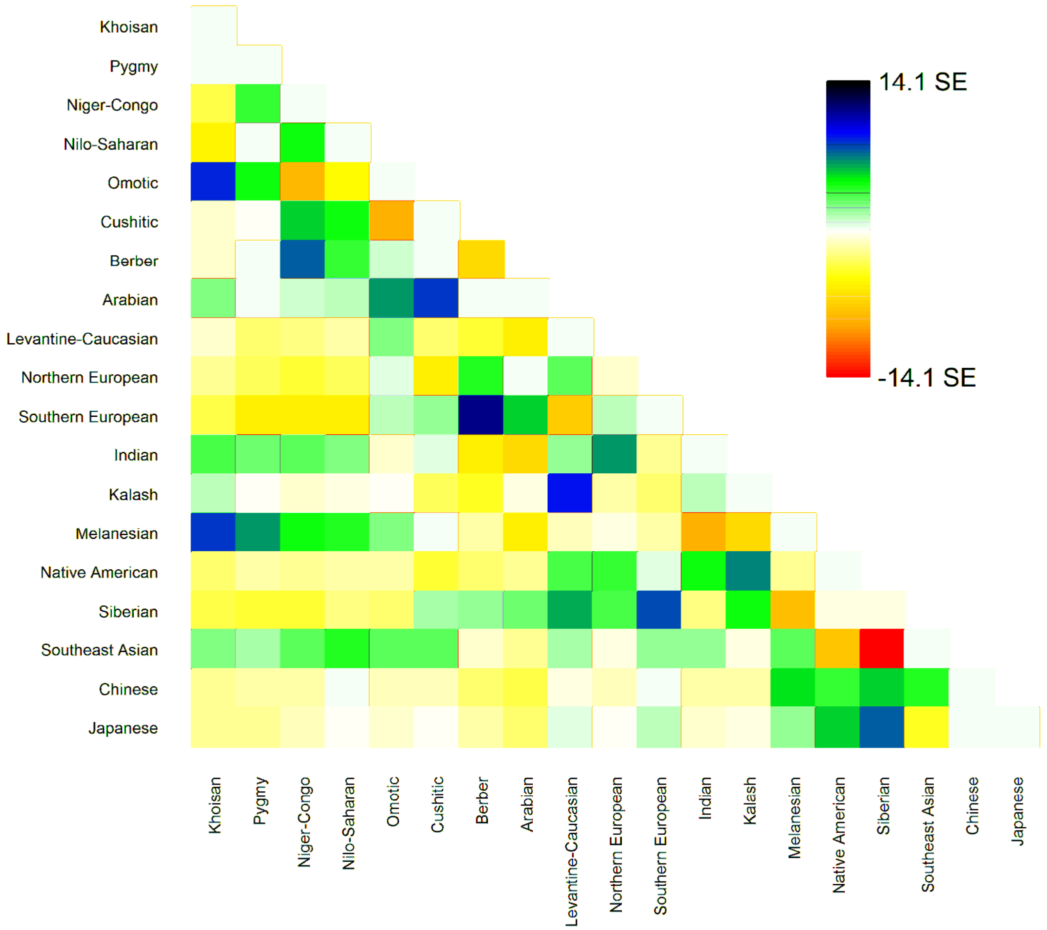

Supplementary Figure S2D. Residual plot corresponding to Supplementary Figure S2C.

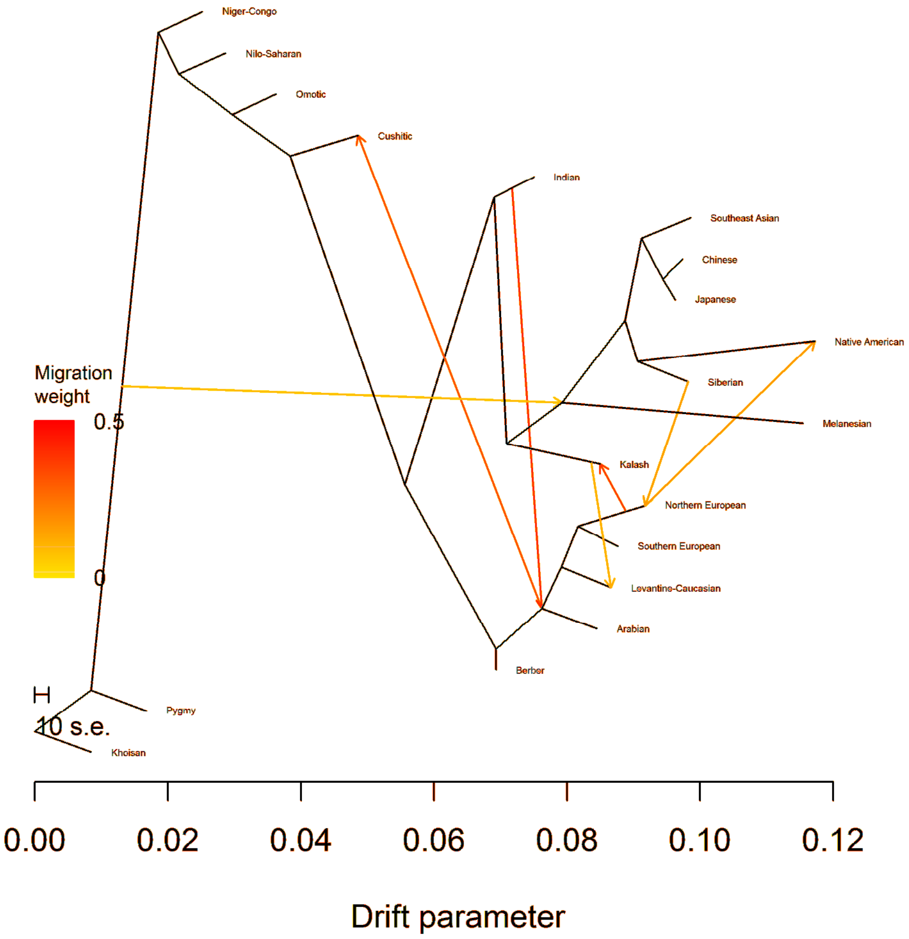

Supplementary Figure S2E. Seven migration events. The seventh event suggests gene flow between Levantine-Caucasian and Kalash ancestries, possibly reflecting Y DNA haplogroup G.

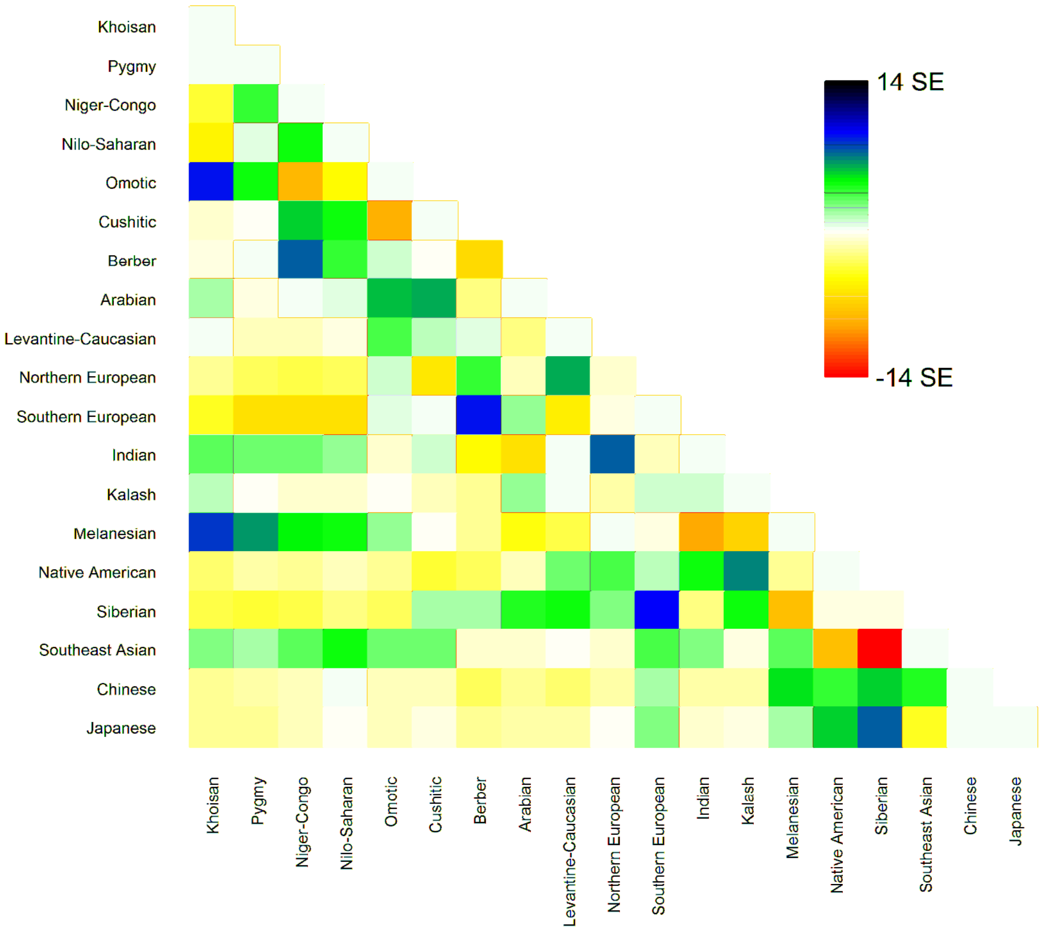

Supplementary Figure S2F. Residual plot corresponding to Supplementary Figure S2E.

Supplementary Text: Derivation of f_3 and f_4 statistics

Consider two putative parental populations A and B and a putative admixed population X . Let the sample sizes in units of alleles be n_A , n_B , and n_X , respectively. At a marker, let the true population allele frequencies be p_A , p_B , and p_X , respectively. Let the corresponding sample estimates be $\hat{p}_A$, $\hat{p}_B$, and $\hat{p}_X$, respectively. The test statistic is defined as $f_3 = \left(p_X - p_A\right)\left(p_X - p_B\right)$ [1, 2]. Under the null hypothesis, the phylogenetic topology is an unrooted star topology (Supplementary Figure S3) and the three allele frequencies are mutually uncorrelated.

Supplementary Figure S3.

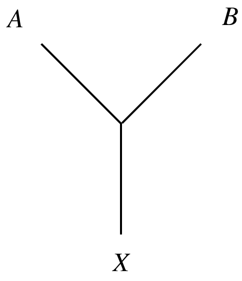

An Unbiased Estimator of f_3

By definition, $f_3 = \left(p_X - p_A\right)\left(p_X - p_B\right) = p_X^2 - p_X p_A - p_{XB} + p_A p_B$. Substituting the three sample estimates yields $\hat{f}_3 = \hat{p}_X^2 - \hat{p}_X \hat{p}_A - \hat{p}_X \hat{p}_B + \hat{p}_A \hat{p}_B$. By the linearity property of expected values, $\mathrm{E}\left(\hat{f}_3\right) = \mathrm{E}\left(\hat{p}_X^2\right) - \mathrm{E}\left(\hat{p}_X \hat{p}_A\right) - \mathrm{E}\left(\hat{p}_X \hat{p}_B\right) + \mathrm{E}\left(\hat{p}_A \hat{p}_B\right)$. Taking each of these four terms one at a time,

$\mathrm{E}\left(\hat{p}_X^2\right) = \mathrm{E}\left(\hat{p}_X\right)\mathrm{E}\left(\hat{p}_X\right) + \mathrm{Var}\left(\hat{p}_X\right)$,

$\mathrm{E}(\hat{p}_X \hat{p}_A) = \mathrm{E}(\hat{p}_X)\mathrm{E}(\hat{p}_A) + \mathrm{Cov}(\hat{p}_X, \hat{p}_A)$,

$\mathrm{E}(\hat{p}_X \hat{p}_B) = \mathrm{E}(\hat{p}_X)\mathrm{E}(\hat{p}_B) + \mathrm{Cov}(\hat{p}_X, \hat{p}_B)$, and

$\mathrm{E}(\hat{p}_A \hat{p}_B) = \mathrm{E}(\hat{p}_A)\mathrm{E}(\hat{p}_B) + \mathrm{Cov}(\hat{p}_A, \hat{p}_B)$.

Under the null hypothesis, the three covariances are zero, so that

$\mathrm{E}(\hat{f}_3) = p_X^2 + \mathrm{Var}(\hat{p}_X) - p_X p_A - p_X p_B + p_A p_B = f_3 + \mathrm{Var}(\hat{p}_X)$.

An unbiased estimator of f_3 is therefore $\hat{f}_3 - \mathrm{Var}(\hat{p}_X)$.

The Variance of f_3

For a binomially distributed random variable $Y \sim Bin(n, p)$ with sample size n and probability p, the first four cumulants are

$\kappa_1 = np$,

$\kappa_2 = np(1-p)$,

$\kappa_3 = np(1-p)(1-2p)$, and

$\kappa_4 = np(1-p)(1-6p(1-p))$.

In terms of the cumulants, the raw moments $\mu'_r = \mathrm{E}(Y^r)$ are

$\mu'_1 = \kappa_1$,

$\mu'_2 = \kappa_2 + \kappa_1^2$,

$\mu'_3 = \kappa_3 + 3\kappa_2\kappa_1 + \kappa_1^3$, and

$\mu'_4 = \kappa_4 + 4\kappa_3\kappa_1 + 3\kappa_2^2 + 6\kappa_2\kappa_1^2 + \kappa_1^4$.

Since $\mu'_r = \mathrm{E}\left(Y^r\right) = \mathrm{E}\left((np)^r\right) = n^r\mathrm{E}\left(p^r\right)$, $\mathrm{E}\left(p^r\right) = \dfrac{\mu'_r}{n^r}$.

By the properties of variance,

$$\begin{aligned}\mathrm{Var}(f_3) &= \mathrm{Var}\left(p_x^2\right) + \mathrm{Var}(p_X p_A) + \mathrm{Var}(p_X p_B) + \mathrm{Var}(p_A p_B) \\ &\quad - 2\mathrm{Cov}\left(p_X^2, p_X p_A\right) - 2\mathrm{Cov}\left(p_X^2, p_X p_B\right) + 2\mathrm{Cov}\left(p_X^2, p_A p_B\right) \\ &\quad + 2\mathrm{Cov}(p_X p_A, p_X p_B) - 2\mathrm{Cov}(p_X p_A, p_A p_B) - 2\mathrm{Cov}(p_X p_B, p_A p_B)\end{aligned}.$$

We now define each of these ten (co)variances in terms of the raw moments:

$$\mathrm{Var}\left(p_X^2\right) = \mathrm{E}\left[\left(p_X^2\right)^2\right] - \left[\mathrm{E}\left(p_X^2\right)\right]^2 = \frac{\mu'_4[X]}{n_X^4} - \left(\frac{\mu'_2[X]}{n_X^2}\right)^2,$$

$$\mathrm{Var}(p_X p_A) = \mathrm{E}\left(p_X^2\right)\mathrm{E}\left(p_A^2\right) - \left[\mathrm{E}(p_X)\right]^2\left[\mathrm{E}(p_A)\right]^2 = \frac{\mu'_2[X]}{n_X^2}\frac{\mu'_2[A]}{n_A^2} - \left(\frac{\mu'_1[X]}{n_X}\right)^2\left(\frac{\mu'_1[A]}{n_A}\right)^2,$$

$$\mathrm{Var}(p_X p_B) = \mathrm{E}\left(p_X^2\right)\mathrm{E}\left(p_B^2\right) - \left[\mathrm{E}(p_X)\right]^2\left[\mathrm{E}(p_B)\right]^2 = \frac{\mu'_2[X]}{n_X^2}\frac{\mu'_2[B]}{n_B^2} - \left(\frac{\mu'_1[X]}{n_X}\right)^2\left(\frac{\mu'_1[B]}{n_B}\right)^2,$$

$$\mathrm{Var}(p_A p_B) = \mathrm{E}\left(p_A^2\right)\mathrm{E}\left(p_B^2\right) - \left[\mathrm{E}(p_A)\right]^2\left[\mathrm{E}(p_B)\right]^2 = \frac{\mu'_2[A]}{n_A^2}\frac{\mu'_2[B]}{n_B^2} - \left(\frac{\mu'_1[A]}{n_A}\right)^2\left(\frac{\mu'_1[B]}{n_B}\right)^2,$$

$$\mathrm{Cov}\left(p_X^2, p_X p_A\right) = \mathrm{E}\left(p_X^2 p_X p_A\right) - \mathrm{E}\left(p_X^2\right)\mathrm{E}(p_X p_A) = \frac{\mu'_3[X]}{n_X^3}\frac{\mu'_1[A]}{n_A} - \frac{\mu'_2[X]}{n_X^2}\frac{\mu'_1[X]\mu'_1[A]}{n_X n_A},$$

$$\mathrm{Cov}\left(p_X^2, p_X p_B\right) = \mathrm{E}\left(p_X^2 p_X p_B\right) - \mathrm{E}\left(p_X^2\right)\mathrm{E}(p_X p_B) = \frac{\mu'_3[X]}{n_X^3}\frac{\mu'_1[B]}{n_B} - \frac{\mu'_2[X]}{n_X^2}\frac{\mu'_1[X]\mu'_1[B]}{n_X n_B},$$

$$\mathrm{Cov}\left(p_X^2, p_A p_B\right) = \mathrm{E}\left(p_X^2 p_A p_B\right) - \mathrm{E}\left(p_X^2\right)\mathrm{E}(p_A p_B) = \mathrm{E}\left(p_X^2\right)\mathrm{E}(p_A p_B) - \mathrm{E}\left(p_X^2\right)\mathrm{E}(p_A p_B) = 0,$$

$$\mathrm{Cov}(p_X p_A, p_X p_B) = \mathrm{E}\left(p_X^2 p_A p_B\right) - \mathrm{E}(p_X p_A)\mathrm{E}(p_X p_B) = \frac{\mu'_1[A]}{n_A}\frac{\mu'_1[B]}{n_B}\left(\frac{\mu'_2[X]}{n_X^2} - \left(\frac{\mu'_1[X]}{n_X}\right)^2\right),$$

$$\mathrm{Cov}(p_X p_A, p_A p_B) = \mathrm{E}\left(p_A^2 p_X p_B\right) - \mathrm{E}(p_X p_A)\mathrm{E}(p_A p_B) = \frac{\mu'_1[X]}{n_X}\frac{\mu'_1[B]}{n_B}\left(\frac{\mu'_2[A]}{n_A^2} - \left(\frac{\mu'_1[A]}{n_A}\right)^2\right),$$

and

$$\mathrm{Cov}(p_X p_B, p_A p_B) = \mathrm{E}(p_B^2 p_X p_A) - \mathrm{E}(p_X p_B)\mathrm{E}(p_A p_B) = \frac{\mu_1'[X]}{n_X}\frac{\mu_1'[A]}{n_A}\left(\frac{\mu_2'[B]}{n_B^2} - \left(\frac{\mu_1'[B]}{n_B}\right)^2\right).$$

Finally, raw sample moments are unbiased estimates of the raw population moments. Therefore, an unbiased estimate of the population variance can be obtained by substitution with the sample allele frequencies and sample sizes.

The f_4 Statistic

Supplementary Figure S4.

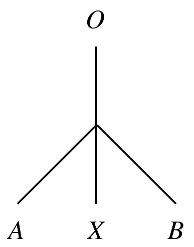

Consider three ingroup populations A, B, X, and an outgroup population O (Supplementary Figure S4). At a marker, let the true population allele frequencies be p_A, p_B, p_X, and p_O, respectively. Let the sample sizes in units of alleles be n_A, n_B, n_X, and n_O, respectively. Let the sample allele frequencies be $\hat{p}_A$, $\hat{p}_B$, $\hat{p}_X$, and $\hat{p}_O$, respectively. The test statistic is defined as $f_4 = (p_O - p_A)(p_X - p_B) = p_O p_X - p_A p_X - p_O p_B + p_A p_B$ [1, 2]. Under the null hypothesis of the unresolved star topology (Supplementary Figure S4),

$$\mathrm{Cov}(p_O, p_X) = \mathrm{Cov}(p_A, p_X) = \mathrm{Cov}(p_O, p_B) = \mathrm{Cov}(p_A, p_B) = 0.$$

Substituting sample estimates yields $\hat{f}_4 = \hat{p}_O\hat{p}_X - \hat{p}_A\hat{p}_X - \hat{p}_O\hat{p}_B + \hat{p}_A\hat{p}_B$. By the linearity property of expected values, $\mathrm{E}\left(\hat{f}_4\right) = \mathrm{E}\left(\hat{p}_O\hat{p}_X\right) - \mathrm{E}\left(\hat{p}_A\hat{p}_X\right) - \mathrm{E}\left(\hat{p}_O\hat{p}_B\right) + \mathrm{E}\left(\hat{p}_A\hat{p}_B\right)$. Taking each term one at a time,

$\mathrm{E}\left(\hat{p}_O\hat{p}_X\right) = \mathrm{E}\left(\hat{p}_O\right)\mathrm{E}\left(\hat{p}_X\right) + \mathrm{Cov}\left(\hat{p}_O, \hat{p}_X\right)$,

$\mathrm{E}\left(\hat{p}_A\hat{p}_X\right) = \mathrm{E}\left(\hat{p}_A\right)\mathrm{E}\left(\hat{p}_X\right) + \mathrm{Cov}\left(\hat{p}_A, \hat{p}_X\right)$,

$\mathrm{E}\left(\hat{p}_O\hat{p}_B\right) = \mathrm{E}\left(\hat{p}_O\right)\mathrm{E}\left(\hat{p}_B\right) + \mathrm{Cov}\left(\hat{p}_O, \hat{p}_B\right)$, and

$\mathrm{E}\left(\hat{p}_A\hat{p}_B\right) = \mathrm{E}\left(\hat{p}_A\right)\mathrm{E}\left(\hat{p}_B\right) + \mathrm{Cov}\left(\hat{p}_A, \hat{p}_B\right)$.

Under the null hypothesis, $\mathrm{E}\left(\hat{f}_4\right) = p_O p_X - p_A p_X - p_O p_B + p_A p_B = f_4$, showing that $\hat{f}_4$ is unbiased.

By the properties of variance,

$$\begin{aligned}\mathrm{Var}\left(f_4\right) = {} & \mathrm{Var}\left(p_O p_X\right) + \mathrm{Var}\left(p_A p_X\right) + \mathrm{Var}\left(p_O p_B\right) + \mathrm{Var}\left(p_A p_B\right) \\ & - 2\mathrm{Cov}\left(p_O p_X, p_A p_X\right) - 2\mathrm{Cov}\left(p_O p_X, p_O p_B\right) + 2\mathrm{Cov}\left(p_O p_X, p_A p_B\right) \\ & + 2\mathrm{Cov}\left(p_A p_X, p_O p_B\right) - 2\mathrm{Cov}\left(p_A p_X, p_A p_B\right) - 2\mathrm{Cov}\left(p_O p_B, p_A p_B\right).\end{aligned}$$

The ten (co)variances are:

$$\mathrm{Var}\left(p_O p_X\right) = \mathrm{E}\left(p_O^2\right)\mathrm{E}\left(p_X^2\right) - \left[\mathrm{E}\left(p_O\right)\right]^2\left[\mathrm{E}\left(p_X\right)\right]^2 = \frac{\mu_2'[O]}{n_O^2}\frac{\mu_2'[X]}{n_X^2} - \left(\frac{\mu_1'[O]}{n_O}\right)^2\left(\frac{\mu_1'[X]}{n_X}\right)^2,$$

$$\mathrm{Var}\left(p_A p_X\right) = \mathrm{E}\left(p_A^2\right)\mathrm{E}\left(p_X^2\right) - \left[\mathrm{E}\left(p_A\right)\right]^2\left[\mathrm{E}\left(p_X\right)\right]^2 = \frac{\mu_2'[A]}{n_A^2}\frac{\mu_2'[X]}{n_X^2} - \left(\frac{\mu_1'[A]}{n_A}\right)^2\left(\frac{\mu_1'[X]}{n_X}\right)^2,$$

$$\mathrm{Var}\left(p_O p_B\right) = \mathrm{E}\left(p_O^2\right)\mathrm{E}\left(p_B^2\right) - \left[\mathrm{E}\left(p_O\right)\right]^2\left[\mathrm{E}\left(p_B\right)\right]^2 = \frac{\mu_2'[O]}{n_O^2}\frac{\mu_2'[B]}{n_B^2} - \left(\frac{\mu_1'[O]}{n_O}\right)^2\left(\frac{\mu_1'[B]}{n_B}\right)^2,$$

$$\mathrm{Var}(p_A p_B) = \mathrm{E}(p_A^2)\mathrm{E}(p_B^2) - [\mathrm{E}(p_A)]^2[\mathrm{E}(p_B)]^2 = \frac{\mu_2'[A]}{n_A^2}\frac{\mu_2'[B]}{n_B^2} - \left(\frac{\mu_1'[A]}{n_A}\right)^2\left(\frac{\mu_1'[B]}{n_B}\right)^2,$$

$$\mathrm{Cov}(p_O p_X, p_A p_X) = \mathrm{E}(p_O)\mathrm{E}(p_A)\mathrm{Var}(p_X) = \frac{\mu_1'[O]}{n_O}\frac{\mu_1'[A]}{n_A}\frac{\kappa_2[X]}{n_X^2},$$

$$\mathrm{Cov}(p_O p_X, p_O p_B) = \mathrm{E}(p_X)\mathrm{E}(p_B)\mathrm{Var}(p_O) = \frac{\mu_1'[X]}{n_X}\frac{\mu_1'[B]}{n_B}\frac{\kappa_2[O]}{n_O^2},$$

$$\mathrm{Cov}(p_O p_X, p_A p_B) = \mathrm{E}(p_O p_X p_A p_B)\text{-}\mathrm{E}(p_O p_X)\mathrm{E}(p_A p_B) = 0,$$

$$\mathrm{Cov}(p_A p_X, p_O p_B) = \mathrm{E}(p_A p_X p_O p_B)\text{-}\mathrm{E}(p_A p_X)\mathrm{E}(p_O p_B) = 0,$$

$$\mathrm{Cov}(p_A p_X, p_A p_B) = \mathrm{E}(p_X)\mathrm{E}(p_B)\mathrm{Var}(p_A) = \frac{\mu_1'[X]}{n_X}\frac{\mu_1'[B]}{n_B}\frac{\kappa_2[A]}{n_A^2},$$

and

$$\mathrm{Cov}(p_O p_B, p_A p_B) = \mathrm{E}(p_O)\mathrm{E}(p_A)\mathrm{Var}(p_B) = \frac{\mu_1'[O]}{n_O}\frac{\mu_1'[A]}{n_A}\frac{\kappa_2[B]}{n_B^2}.$$

As with the f_3 statistic, raw sample moments are unbiased estimates of the raw population moments. Therefore, an unbiased estimate of the population variance can be obtained by substitution with the sample allele frequencies and sample sizes.

Combining Markers

Assume markers are uncorrelated. To account for variance within markers as well as between markers, we calculate a *z*-score per marker and then combine *z*-scores. Specifically, let $z_l = \frac{f_{3_l}}{\sqrt{\mathrm{Var}(f_{3_l})}}$ and $z_c = \sum_{l=1}^{L} z_l w_l$, in which L markers are assigned equal weight such that

$w_l = \frac{1}{\sqrt{L}}$. Then, z_c follows the standard normal distribution. Admixture in the history of X is expected to lead to $z_c < 0$, which implies a one-tailed test.

Similarly, let $z_l = \frac{f_{4_l}}{\sqrt{\mathrm{Var}\left(f_{4_l}\right)}}$ and $z_c = \sum_{l=1}^{L} z_l w_l$, in which L markers are assigned equal weight such that $w_l = \frac{1}{\sqrt{L}}$. Then, z_c follows the standard normal distribution.

Estimation of Mixture Proportions

Consider an admixed population X with a mixture proportion α from parental population B and $\beta = 1 - \alpha$ from parental population C. Further, suppose population A is a sibling to population B and population O is an outgroup. Reich *et al.*[1] proposed an estimator of the mixture proportion $\alpha = \frac{\sum f_4(A,O,X,C)}{\sum f_4(A,O,B,C)}$, in which $f_4(A,O,X,C) = (p_A - p_O)(p_X - p_C)$ and $f_4(A,O,B,C) = (p_A - p_O)(p_B - p_C)$ for each marker and the summations are over all markers.

Supplementary References

1. Reich D, Thangaraj K, Patterson N, Price AL, Singh L. Reconstructing Indian population history. *Nature* **461**, 489-494 (2009).
2. Patterson N*, et al.* Ancient admixture in human history. *Genetics* **192**, 1065-1093 (2012).

Supplementary Table S1. Sample description.

Region	Sample	Longitude[1]	Latitude[2]	Sample Size	Reference (PMID)
Africa	!Xun	17.6667	-14.6667	17	22997136
Africa	!Xun	17.6667	-14.6667	8	23516368
Africa	/Gui and //Gana	24.5019889	-23.3408495	9	22997136
Africa	≠Khomani	20.78333333	-26.98333333	32	22997136
Africa	Agaw	41	12	10	22726845
Africa	Algeria	3.216666667	36.76666667	19	22253600
Africa	amaXhosa	27	-32	15	23516368
Africa	Amhara	39	10	24	22726845
Africa	Amhara	39	10	7	20531471
Africa	Angolan !Xun	19	-17	4	23516368
Africa	Anuak	34	8	22	22726845
Africa	Ari Blacksmith	37	6	11	22726845
Africa	Ari Cultivator	37	6	21	22726845
Africa	Bamoun	5.5	10.8	18	20080753
Africa	Bantu Kenya	37	-3	10	18292342
Africa	Bantu South Africa	25.2	-25.8	6	18292342
Africa	Basters	17.08333333	-23.31666667	30	23516368
Africa	Biaka Pygmy	17	4	21	18292342
Africa	Brong	7.5	-2	8	20080753
Africa	Bulala	13	18	15	20080753
Africa	Coloured Colesberg	25.1	-30.71666667	18	22997136
Africa	Coloured District 6	18.43416667	-33.93277778	8	23516368
Africa	Coloured Eastern Cape	27	-32	7	23516368
Africa	Coloured Northern Cape	22	-30	10	23516368
Africa	Coloured Wellington	18.98333333	-33.63333333	17	22997136
Africa	Egypt	31.21666667	30.03333333	19	22253600
Africa	Egypt	30	26	12	20531471
Africa	Ethiopia Jew	38.74	9.03	12	20531471
Africa	Ethiopian Somali	42	9	8	22726845
Africa	Fang	2.5	13	15	20080753
Africa	Gumuz	35.73305556	10.63888889	16	22726845
Africa	Hausa	9.1	7.5	12	20080753
Africa	Ju/'hoan	20.5022	-19.5931	3	23516368
Africa	Ju/'hoansi	20.5022	-19.5931	2	22997136
Africa	Kaba	8	16.8	16	20080753
Africa	Karretjie	25.1	-30.71666667	16	22997136
Africa	Khwe	24.275	-17.50388889	16	22997136
Africa	Kongo	-5.5	15	9	20080753
Africa	Libya	20.06666667	32.11666667	17	22253600
Africa	LWK	34.58333333	0.5	87	23128226
Africa	Mada	10.8	14.1	12	20080753

Africa	Mandenka	-12	12	21	18292342
Africa	Mbuti Pygmy	29	1	10	18292342
Africa	MKK	35.28333333	0.516666667	93	20811451
Africa	Morocco	-6	32	10	20531471
Africa	Morocco Jew	-6	32	15	20531471
Africa	Mozabite	3	32	28	18292342
Africa	Nama	17.85	-22.57	19	22997136
Africa	North Morocco	-6.84165	34.020882	18	22253600
Africa	Oromo	37	8	7	20531471
Africa	Oromo	37	8	21	22726845
Africa	Sahrawi	-13	27	18	22253600
Africa	San	20	-21	2	18292342
Africa	SE Bantu	27.93333333	-29.46666667	18	22997136
Africa	Somali	42	9	22	22726845
Africa	South Morocco	-9.6	30.43333333	16	22253600
Africa	Sudanese	31.6	4.85	20	22726845
Africa	SW Bantu	17.85	-22.57	11	22997136
Africa	Tigray	38	9	5	20531471
Africa	Tunisia	10.18333333	36.8	18	22253600
Africa	Tygray	38	9	20	22726845
Africa	Wolayta	37	6	6	22726845
Africa	Yoruba	5	8	21	18292342
Africa	YRI	3.916666667	7.396388889	88	23128226
America	ASW	-97.41666667	35.21666667	57	23128226
America	CEU	-111.6608333	40.24444444	85	23128226
America	CLM	-75.57513611	6.235925	60	23128226
America	Colombian	-68	3	2	18292342
America	Karitiana	-63	-10	2	18292342
America	Maya	-91	19	18	18292342
America	MXL	-99.36666667	19.05	60	23128226
America	Pima	-108	29	2	18292342
America	PUR	-67.13972222	18.20111111	55	23128226
America	Surui	-62	-11	1	18292342
Asia	Arunthathiyar	80.27	13.09	4	20531471
Asia	Balochi	66.5	30.5	24	18292342
Asia	Brahui	66.5	30.5	24	18292342
Asia	Burusho	74	36.5	23	18292342
Asia	Cambodian	105	12	8	18292342
Asia	CHB	116.3916667	39.91388889	97	23128226
Asia	CHS	114.1	22.55	92	23128226
Asia	Dai	100	21	10	18292342
Asia	Daur	124	48.5	9	18292342
Asia	Han North China	114	39	10	18292342

Asia	Han South China	114	36	34	18292342
Asia	Hazara	70	33.5	22	18292342
Asia	Hezhen	133.5	47.5	8	18292342
Asia	Japanese	138	38	28	18292342
Asia	JPT	139.6917	35.68950556	89	23128226
Asia	Kalash	71.5	36	23	18292342
Asia	Kochi Jew	76.28	9.97	4	20531471
Asia	Lahu	100	22	7	18292342
Asia	Makrani	64	26	25	18292342
Asia	Malaya	100	7	1	20531471
Asia	Miaozu	109	28	10	18292342
Asia	Mongolia	111	45	10	18292342
Asia	Mumbai Jew	72.82583333	18.975	4	20531471
Asia	Naxi	100	26	7	18292342
Asia	North Kannadi	76.972	8.5074	8	20531471
Asia	Oroqen	126.5	50.5	9	18292342
Asia	Paniya	76.972	8.5074	3	20531471
Asia	Pathan	70.5	33.5	22	18292342
Asia	She	119	27	9	18292342
Asia	Sindhi	69	25.5	24	18292342
Asia	Singapore Chinese	103.8	1.3	96	19700652
Asia	Singapore Indian	103.8	1.3	83	19700652
Asia	Singapore Malay	103.8	1.3	89	19700652
Asia	Tu	101	36	10	18292342
Asia	Tujia	109	29	10	18292342
Asia	Uygur	81	44	10	18292342
Asia	Uzbekistan	69.21666667	41.26666667	15	20531471
Asia	Uzbekistan Jew	69.21666667	41.26666667	2	20531471
Asia	Xibo	81.5	43.5	9	18292342
Asia	Yakut	129.5	63	25	18292342
Asia	Yizu	103	28	10	18292342
Caucasus	Adygei	39	44	15	18292342
Caucasus	Armenia	44.51666667	40.18333333	19	20531471
Caucasus	Azerbaijan Jew	47.7	40.3	8	20531471
Caucasus	Georgia	44.78333333	41.71666667	20	20531471
Caucasus	Georgia Jew	44.78333333	41.71666667	4	20531471
Caucasus	Lezgian	46.88333333	43.1	18	20531471
Europe	Ashkenazi East	NA	NA	9	20531471
Europe	Ashkenazi West	NA	NA	9	20531471
Europe	Basque	0	43	24	18292342
Europe	Basque	-1.916944444	42.88194444	20	22253600
Europe	Belorussia	27.55	53.91666667	9	20531471
Europe	Bulgaria Jew	25.5	42.75	9	20531471

Europe	Chuvash	47.1	55.55	17	20531471
Europe	FIN	24.9375	60.17083	93	23128226
Europe	French	2	46	28	18292342
Europe	GBR	0.73	51.19	88	23128226
Europe	Hungary	19.25	47.43333333	19	20531471
Europe	IBS	-5.667607	40.961612	14	23128226
Europe	Lithuania	25.31666667	54.68333333	10	20531471
Europe	North Italian	10	46	11	18292342
Europe	Orcadian	-3	59	13	18292342
Europe	Romania	26.1	44.41666667	16	20531471
Europe	Romania Jew	26.1	44.41666667	3	20531471
Europe	Russian	40	61	24	18292342
Europe	Sardinian	9	40	27	18292342
Europe	Spain	-3.7	40.43333333	12	20531471
Europe	TSI	11.25	43.78333333	98	23128226
Europe	Tuscan	11	43	6	18292342
Middle East	Bedouin	35	31	45	18292342
Middle East	Cyprus	33	35	12	20531471
Middle East	Druze	35	32	41	18292342
Middle East	Iran	53	32	19	20531471
Middle East	Iran Jew	53	32	4	20531471
Middle East	Iraq Jew	44	33	10	20531471
Middle East	Jordan	35.93333333	31.95	20	20531471
Middle East	Lebanon	35.53333333	33.9	75	23468648
Middle East	Lebanon	35.53333333	33.9	7	20531471
Middle East	Palestinian	35	32	44	18292342
Middle East	Qatari	51.53333333	25.28666667	16	20579625
Middle East	Samaritan	34.78333333	32.06666667	2	20531471
Middle East	Saudi Arabia	45	24	20	20531471
Middle East	Syria	36.3	33.5	16	20531471
Middle East	Turkey	30	40	19	20531471
Middle East	Turkey Jew	30	40	10	20531471
Middle East	Yemen	44.20638889	15.34833333	8	20531471
Middle East	Yemen Jew	44.20638889	15.34833333	15	20531471
Oceania	Melanesian	155	-6	5	18292342
Oceania	Papuan	143	-4	2	18292342

[1] Positive values indicate east and negative values indicate west.

[2] Positive values indicate north and negative values indicate south.

Supplementary Table 2. f_3 statistics.

Parent Ancestry *A*	Parent Ancestry *B*	Test Ancestry *X*	*Z*-score	Unadjusted *P*-value	Adjusted *P*-value
Southern European	Niger-Congo	Berber	-129.54	0	0
Southern European	Niger-Congo	Cushitic	-124.39	0	0
Southern European	Khoisan	Omotic	-102.52	0	0
Southern European	Khoisan	Cushitic	-102.48	0	0
Southern European	Nilo-Saharan	Berber	-98.02	0	0
Niger-Congo	Arabian	Cushitic	-97.76	0	0
Southern European	Nilo-Saharan	Cushitic	-97.58	0	0
Levantine-Caucasian	Niger-Congo	Cushitic	-95.69	0	0
Khoisan	Northern European	Omotic	-94.26	0	0
Levantine-Caucasian	Khoisan	Omotic	-94.15	0	0
Southern European	Khoisan	Berber	-90.69	0	0
Khoisan	Chinese	Omotic	-85.40	0	0
Niger-Congo	Northern European	Berber	-85.03	0	0
Khoisan	Arabian	Omotic	-84.74	0	0
Levantine-Caucasian	Khoisan	Cushitic	-83.61	0	0
Southern European	Pygmy	Cushitic	-80.98	0	0
Levantine-Caucasian	Niger-Congo	Berber	-79.47	0	0
Khoisan	Arabian	Cushitic	-77.93	0	0
Niger-Congo	Northern European	Cushitic	-77.75	0	0
Nilo-Saharan	Arabian	Cushitic	-74.33	0	0
Southern European	Pygmy	Berber	-73.43	0	0
Khoisan	Northern European	Cushitic	-71.98	0	0
Khoisan	Native American	Omotic	-71.53	0	0
Southern European	Pygmy	Omotic	-70.16	0	0
Levantine-Caucasian	Nilo-Saharan	Cushitic	-69.53	0	0
Niger-Congo	Arabian	Berber	-68.42	0	0
Khoisan	Japanese	Omotic	-68.27	0	0
Southeast Asian	Khoisan	Omotic	-66.99	0	0
Khoisan	Indian	Omotic	-65.11	0	0
Khoisan	Northern European	Berber	-62.90	0	0
Levantine-Caucasian	Pygmy	Omotic	-62.41	0	0
Pygmy	Arabian	Cushitic	-61.39	0	0
Berber	Khoisan	Omotic	-60.79	0	0
Levantine-Caucasian	Pygmy	Cushitic	-60.49	0	0
Pygmy	Northern European	Omotic	-59.60	0	0
Pygmy	Arabian	Omotic	-58.20	0	0
Khoisan	Siberian	Omotic	-57.99	0	0
Nilo-Saharan	Northern European	Cushitic	-57.05	0	0
Nilo-Saharan	Northern European	Berber	-57.00	0	0
Khoisan	Kalash	Omotic	-56.82	0	0
Levantine-Caucasian	Khoisan	Berber	-55.39	0	0
Levantine-Caucasian	Chinese	Indian	-50.91	0	0
Niger-Congo	Native American	Indian	-50.43	0	0
Southern European	Chinese	Indian	-49.47	0	0
Pygmy	Northern European	Cushitic	-49.18	0	0
Levantine-Caucasian	Nilo-Saharan	Berber	-47.65	0	0
Pygmy	Chinese	Omotic	-47.34	0	0
Southern European	Niger-Congo	Omotic	-46.95	0	0
Melanesian	Khoisan	Omotic	-45.32	0	0

Southern European	Native American	Northern European	-44.52	0	0
Melanesian	Southern European	Indian	-44.45	0	0
Khoisan	Chinese	Nilo-Saharan	-44.34	0	0
Melanesian	Niger-Congo	Indian	-43.75	0	0
Melanesian	Levantine-Caucasian	Indian	-43.70	0	0
Southern European	Omotic	Berber	-43.49	0	0
Khoisan	Arabian	Berber	-42.95	0	0
Southern European	Nilo-Saharan	Omotic	-42.15	0	0
Nilo-Saharan	Arabian	Berber	-40.94	0	0
Pygmy	Northern European	Berber	-40.73	0	0
Southern European	Khoisan	Nilo-Saharan	-39.91	0	0
Khoisan	Native American	Indian	-39.32	0	0
Pygmy	Native American	Omotic	-38.34	0	0
Khoisan	Northern European	Nilo-Saharan	-38.33	0	0
Southeast Asian	Levantine-Caucasian	Indian	-38.30	0	0
Japanese	Pygmy	Omotic	-38.00	0	0
Southeast Asian	Pygmy	Omotic	-37.86	0	0
Levantine-Caucasian	Niger-Congo	Omotic	-37.33	2.99E-305	8.69E-302
Levantine-Caucasian	Khoisan	Nilo-Saharan	-36.62	5.95E-294	1.73E-290
Pygmy	Indian	Omotic	-36.57	4.71E-293	1.37E-289
Levantine-Caucasian	Pygmy	Berber	-36.27	2.14E-288	6.21E-285
Khoisan	Cushitic	Omotic	-36.20	3.53E-287	1.02E-283
Niger-Congo	Arabian	Omotic	-35.89	2.02E-282	5.86E-279
Niger-Congo	Chinese	Japanese	-35.62	3.36E-278	9.76E-275
Niger-Congo	Native American	Omotic	-35.47	6.64E-276	1.93E-272
Southern European	Chinese	Japanese	-35.45	1.43E-275	4.15E-272
Niger-Congo	Native American	Berber	-35.44	1.83E-275	5.32E-272
Niger-Congo	Northern European	Omotic	-34.86	1.34E-266	3.89E-263
Southern European	Southeast Asian	Indian	-34.82	6.63E-266	1.93E-262
Berber	Pygmy	Omotic	-34.24	3.12E-257	9.07E-254
Chinese	Northern European	Japanese	-34.19	2.00E-256	5.81E-253
Pygmy	Kalash	Omotic	-34.07	9.84E-255	2.86E-251
Niger-Congo	Native American	Cushitic	-33.52	1.04E-246	3.03E-243
Melanesian	Niger-Congo	Omotic	-33.44	1.69E-245	4.92E-242
Niger-Congo	Kalash	Cushitic	-33.11	1.23E-240	3.58E-237
Levantine-Caucasian	Nilo-Saharan	Omotic	-32.54	1.27E-232	3.68E-229
Levantine-Caucasian	Chinese	Japanese	-32.11	1.85E-226	5.39E-223
Pygmy	Siberian	Omotic	-32.08	3.64E-226	1.06E-222
Melanesian	Northern European	Indian	-31.71	5.45E-221	1.58E-217
Levantine-Caucasian	Japanese	Indian	-31.57	4.56E-219	1.32E-215
Nilo-Saharan	Northern European	Omotic	-31.50	4.10E-218	1.19E-214
Khoisan	Chinese	Japanese	-31.29	3.55E-215	1.03E-211
Niger-Congo	Chinese	Indian	-30.57	1.60E-205	4.64E-202
Nilo-Saharan	Arabian	Omotic	-30.55	3.11E-205	9.04E-202
Khoisan	Chinese	Pygmy	-30.51	9.93E-205	2.89E-201
Pygmy	Arabian	Berber	-30.22	7.05E-201	2.05E-197
Khoisan	Chinese	Indian	-30.20	1.26E-200	3.67E-197
Niger-Congo	Chinese	Omotic	-29.83	7.43E-196	2.16E-192
Khoisan	Native American	Cushitic	-28.86	1.89E-183	5.49E-180
Nilo-Saharan	Native American	Indian	-28.57	8.33E-180	2.42E-176
Nilo-Saharan	Chinese	Japanese	-28.02	4.74E-173	1.38E-169

Khoisan	Chinese	Cushitic	-28.02	5.18E-173	1.50E-169
Southern European	Japanese	Indian	-27.96	2.62E-172	7.63E-169
Niger-Congo	Kalash	Berber	-26.34	3.46E-153	1.01E-149
Chinese	Arabian	Japanese	-26.10	1.94E-150	5.64E-147
Nilo-Saharan	Native American	Omotic	-25.30	1.54E-141	4.49E-138
Southern European	Pygmy	Nilo-Saharan	-25.28	2.99E-141	8.69E-138
Chinese	Arabian	Indian	-25.17	4.34E-140	1.26E-136
Chinese	Native American	Japanese	-25.15	7.24E-140	2.11E-136
Khoisan	Northern European	Pygmy	-25.10	2.59E-139	7.54E-136
Berber	Khoisan	Cushitic	-25.09	3.45E-139	1.00E-135
Melanesian	Pygmy	Omotic	-24.95	9.62E-138	2.80E-134
Melanesian	Niger-Congo	Cushitic	-24.60	6.96E-134	2.02E-130
Khoisan	Native American	Nilo-Saharan	-24.17	2.20E-129	6.40E-126
Southern European	Khoisan	Pygmy	-24.04	5.69E-128	1.66E-124
Khoisan	Japanese	Nilo-Saharan	-24.01	1.10E-127	3.21E-124
Nilo-Saharan	Chinese	Omotic	-23.85	5.47E-126	1.59E-122
Cushitic	Chinese	Japanese	-23.46	4.71E-122	1.37E-118
Khoisan	Native American	Pygmy	-23.01	1.71E-117	4.99E-114
Southeast Asian	Niger-Congo	Omotic	-22.93	1.08E-116	3.14E-113
Berber	Chinese	Japanese	-22.90	2.11E-116	6.13E-113
Khoisan	Kalash	Cushitic	-22.81	1.80E-115	5.24E-112
Melanesian	Nilo-Saharan	Omotic	-22.52	1.29E-112	3.75E-109
Melanesian	Niger-Congo	Berber	-22.32	1.18E-110	3.44E-107
Southeast Asian	Khoisan	Nilo-Saharan	-22.22	9.87E-110	2.87E-106
Levantine-Caucasian	Khoisan	Pygmy	-22.18	2.71E-109	7.87E-106
Niger-Congo	Japanese	Omotic	-21.70	9.63E-105	2.80E-101
Melanesian	Nilo-Saharan	Indian	-21.61	7.12E-104	2.07E-100
Niger-Congo	Chinese	Nilo-Saharan	-21.57	1.70E-103	4.94E-100
Levantine-Caucasian	Pygmy	Nilo-Saharan	-21.54	3.46E-103	1.01E-99
Melanesian	Arabian	Indian	-21.42	4.81E-102	1.40E-98
Southern European	Khoisan	Arabian	-21.30	5.22E-101	1.52E-97
Niger-Congo	Chinese	Cushitic	-21.20	4.72E-100	1.37E-96
Chinese	Northern European	Indian	-21.18	6.92E-100	2.01E-96
Khoisan	Native American	Berber	-20.77	3.77E-96	1.10E-92
Pygmy	Chinese	Japanese	-20.29	7.17E-92	2.08E-88
Khoisan	Arabian	Nilo-Saharan	-20.29	8.60E-92	2.50E-88
Southern European	Khoisan	Niger-Congo	-20.25	1.93E-91	5.60E-88
Niger-Congo	Kalash	Omotic	-20.20	4.52E-91	1.31E-87
Southeast Asian	Khoisan	Pygmy	-19.95	7.36E-89	2.14E-85
Pygmy	Northern European	Nilo-Saharan	-19.88	3.22E-88	9.37E-85
Niger-Congo	Siberian	Omotic	-19.85	5.03E-88	1.46E-84
Khoisan	Japanese	Pygmy	-19.70	1.14E-86	3.33E-83
Khoisan	Northern European	Niger-Congo	-19.26	5.61E-83	1.63E-79
Chinese	Indian	Japanese	-19.24	8.28E-83	2.41E-79
Southern European	Omotic	Cushitic	-19.20	1.89E-82	5.48E-79
Pygmy	Chinese	Nilo-Saharan	-18.91	5.03E-80	1.46E-76
Southeast Asian	Siberian	Japanese	-18.89	7.24E-80	2.11E-76
Khoisan	Chinese	Niger-Congo	-18.88	7.74E-80	2.25E-76
Melanesian	Niger-Congo	Nilo-Saharan	-18.87	1.02E-79	2.97E-76
Southern European	Pygmy	Niger-Congo	-18.23	1.37E-74	3.99E-71
Khoisan	Native American	Siberian	-18.16	5.17E-74	1.50E-70

Niger-Congo	Native American	Japanese	-17.73	1.25E-70	3.63E-67
Chinese	Northern European	Siberian	-17.40	3.92E-68	1.14E-64
Nilo-Saharan	Kalash	Cushitic	-17.24	6.38E-67	1.85E-63
Levantine-Caucasian	Khoisan	Niger-Congo	-17.22	9.43E-67	2.74E-63
Southeast Asian	Arabian	Indian	-17.19	1.51E-66	4.39E-63
Southern European	Niger-Congo	Arabian	-17.14	3.43E-66	9.97E-63
Niger-Congo	Native American	Siberian	-16.78	1.71E-63	4.96E-60
Omotic	Northern European	Berber	-16.62	2.68E-62	7.79E-59
Chinese	Kalash	Japanese	-16.32	3.56E-60	1.04E-56
Japanese	Nilo-Saharan	Omotic	-16.07	2.07E-58	6.01E-55
Southeast Asian	Nilo-Saharan	Omotic	-15.85	7.03E-57	2.04E-53
Khoisan	Chinese	Southeast Asian	-15.83	9.85E-57	2.86E-53
Chinese	Omotic	Japanese	-15.81	1.29E-56	3.75E-53
Pygmy	Native American	Indian	-15.74	3.86E-56	1.12E-52
Southeast Asian	Native American	Japanese	-15.65	1.68E-55	4.87E-52
Niger-Congo	Native American	Nilo-Saharan	-15.59	3.96E-55	1.15E-51
Nilo-Saharan	Kalash	Omotic	-15.53	1.03E-54	2.99E-51
Southeast Asian	Niger-Congo	Indian	-15.23	1.07E-52	3.11E-49
Melanesian	Khoisan	Pygmy	-15.14	4.46E-52	1.30E-48
Khoisan	Arabian	Pygmy	-15.08	1.03E-51	3.01E-48
Nilo-Saharan	Native American	Cushitic	-15.08	1.11E-51	3.23E-48
Southeast Asian	Northern European	Indian	-14.94	9.47E-51	2.75E-47
Southern European	Chinese	Northern European	-14.77	1.20E-49	3.48E-46
Khoisan	Indian	Pygmy	-14.39	2.83E-47	8.23E-44
Khoisan	Native American	Japanese	-14.38	3.66E-47	1.06E-43
Berber	Khoisan	Nilo-Saharan	-14.32	7.87E-47	2.29E-43
Melanesian	Cushitic	Indian	-14.26	2.07E-46	6.01E-43
Melanesian	Southern European	Kalash	-14.09	2.27E-45	6.59E-42
Southern European	Chinese	Siberian	-14.05	3.98E-45	1.16E-41
Khoisan	Indian	Nilo-Saharan	-13.71	4.45E-43	1.29E-39
Nilo-Saharan	Siberian	Omotic	-13.54	4.40E-42	1.28E-38
Khoisan	Siberian	Pygmy	-13.52	5.99E-42	1.74E-38
Pygmy	Arabian	Nilo-Saharan	-13.31	9.82E-41	2.86E-37
Niger-Congo	Siberian	Cushitic	-13.19	4.86E-40	1.41E-36
Southern European	Native American	Indian	-13.16	7.25E-40	2.11E-36
Melanesian	Levantine-Caucasian	Kalash	-12.92	1.69E-38	4.91E-35
Niger-Congo	Chinese	Southeast Asian	-12.86	3.84E-38	1.12E-34
Levantine-Caucasian	Pygmy	Niger-Congo	-12.72	2.22E-37	6.44E-34
Southern European	Niger-Congo	Nilo-Saharan	-12.68	3.63E-37	1.06E-33
Niger-Congo	Japanese	Indian	-12.57	1.45E-36	4.22E-33
Cushitic	Northern European	Berber	-11.99	2.01E-33	5.83E-30
Melanesian	Chinese	Japanese	-11.70	6.67E-32	1.94E-28
Pygmy	Cushitic	Omotic	-11.61	1.93E-31	5.62E-28
Pygmy	Northern European	Niger-Congo	-11.57	3.08E-31	8.97E-28
Niger-Congo	Siberian	Indian	-11.36	3.37E-30	9.81E-27
Cushitic	Native American	Indian	-11.32	5.41E-30	1.57E-26
Chinese	Siberian	Japanese	-11.30	6.44E-30	1.87E-26
Khoisan	Siberian	Nilo-Saharan	-11.28	8.44E-30	2.45E-26
Japanese	Arabian	Indian	-10.83	1.23E-27	3.57E-24
Khoisan	Japanese	Indian	-10.76	2.69E-27	7.83E-24
Southeast Asian	Khoisan	Indian	-10.47	5.82E-26	1.69E-22

Levantine-Caucasian	Niger-Congo	Nilo-Saharan	-10.36	1.91E-25	5.56E-22
Melanesian	Khoisan	Indian	-10.31	3.09E-25	8.99E-22
Niger-Congo	Indian	Omotic	-10.03	5.72E-24	1.66E-20
Nilo-Saharan	Native American	Berber	-9.98	9.35E-24	2.72E-20
Nilo-Saharan	Indian	Omotic	-9.85	3.54E-23	1.03E-19
Levantine-Caucasian	Native American	Indian	-9.77	7.85E-23	2.28E-19
Pygmy	Kalash	Cushitic	-9.72	1.27E-22	3.70E-19
Berber	Khoisan	Pygmy	-9.67	1.94E-22	5.63E-19
Khoisan	Japanese	Cushitic	-9.67	2.02E-22	5.86E-19
Berber	Pygmy	Cushitic	-9.46	1.53E-21	4.44E-18
Khoisan	Native American	Kalash	-9.15	2.78E-20	8.08E-17
Cushitic	Chinese	Indian	-9.05	7.44E-20	2.16E-16
Khoisan	Kalash	Pygmy	-9.00	1.13E-19	3.30E-16
Southern European	Cushitic	Berber	-8.92	2.24E-19	6.51E-16
Berber	Niger-Congo	Cushitic	-8.91	2.57E-19	7.47E-16
Niger-Congo	Japanese	Nilo-Saharan	-8.72	1.37E-18	3.99E-15
Southeast Asian	Khoisan	Cushitic	-8.63	3.05E-18	8.86E-15
Melanesian	Southern European	Berber	-8.62	3.28E-18	9.53E-15
Japanese	Northern European	Siberian	-8.57	5.08E-18	1.48E-14
Southeast Asian	Pygmy	Nilo-Saharan	-8.53	7.48E-18	2.17E-14
Southeast Asian	Niger-Congo	Nilo-Saharan	-8.49	1.03E-17	2.99E-14
Niger-Congo	Northern European	Nilo-Saharan	-8.37	2.80E-17	8.13E-14
Niger-Congo	Japanese	Cushitic	-8.28	5.92E-17	1.72E-13
Omotic	Arabian	Cushitic	-8.17	1.52E-16	4.43E-13
Niger-Congo	Kalash	Indian	-8.16	1.61E-16	4.68E-13
Nilo-Saharan	Chinese	Indian	-8.09	3.06E-16	8.90E-13
Khoisan	Chinese	Siberian	-8.04	4.53E-16	1.32E-12
Pygmy	Native American	Nilo-Saharan	-7.87	1.71E-15	4.97E-12
Khoisan	Indian	Cushitic	-7.85	2.08E-15	6.05E-12
Cushitic	Native American	Berber	-7.77	3.88E-15	1.13E-11
Khoisan	Northern European	Indian	-7.77	3.99E-15	1.16E-11
Berber	Khoisan	Niger-Congo	-7.73	5.41E-15	1.57E-11
Khoisan	Siberian	Cushitic	-7.72	5.97E-15	1.74E-11
Khoisan	Siberian	Indian	-7.69	7.11E-15	2.07E-11
Japanese	Pygmy	Nilo-Saharan	-7.66	9.05E-15	2.63E-11
Melanesian	Siberian	Japanese	-7.46	4.28E-14	1.24E-10
Nilo-Saharan	Kalash	Berber	-7.29	1.52E-13	4.42E-10
Niger-Congo	Siberian	Japanese	-7.20	2.93E-13	8.51E-10
Melanesian	Southern European	Northern European	-7.18	3.47E-13	1.01E-09
Berber	Pygmy	Niger-Congo	-7.11	5.89E-13	1.71E-09
Khoisan	Kalash	Berber	-7.10	6.25E-13	1.82E-09
Levantine-Caucasian	Native American	Northern European	-6.99	1.42E-12	4.14E-09
Nilo-Saharan	Native American	Japanese	-6.94	1.90E-12	5.52E-09
Southeast Asian	Siberian	Chinese	-6.66	1.33E-11	3.86E-08
Berber	Nilo-Saharan	Cushitic	-6.65	1.47E-11	4.27E-08
Southeast Asian	Niger-Congo	Cushitic	-6.62	1.82E-11	5.29E-08
Levantine-Caucasian	Chinese	Siberian	-6.32	1.28E-10	3.73E-07
Pygmy	Native American	Cushitic	-6.21	2.73E-10	7.93E-07
Pygmy	Chinese	Indian	-6.02	8.82E-10	2.56E-06
Levantine-Caucasian	Omotic	Berber	-5.56	1.32E-08	3.84E-05
Melanesian	Native American	Japanese	-5.39	3.57E-08	1.04E-04

Nilo-Saharan	Native American	Siberian	-5.35	4.42E-08	1.29E-04
Melanesian	Niger-Congo	Japanese	-5.24	8.09E-08	2.35E-04
Pygmy	Arabian	Niger-Congo	-5.21	9.61E-08	2.79E-04
Niger-Congo	Chinese	Berber	-5.17	1.16E-07	3.36E-04
Pygmy	Chinese	Niger-Congo	-5.14	1.40E-07	4.07E-04
Southern European	Native American	Kalash	-5.05	2.22E-07	6.46E-04
Southern European	Japanese	Siberian	-4.98	3.15E-07	9.15E-04
Pygmy	Indian	Nilo-Saharan	-4.96	3.56E-07	1.04E-03
Khoisan	Siberian	Japanese	-4.84	6.36E-07	1.85E-03
Berber	Pygmy	Nilo-Saharan	-4.83	6.89E-07	2.00E-03
Khoisan	Chinese	Berber	-4.80	7.85E-07	2.28E-03
Melanesian	Berber	Indian	-4.55	2.67E-06	7.76E-03
Southern European	Khoisan	Indian	-4.48	3.69E-06	0.0107
Melanesian	Nilo-Saharan	Cushitic	-4.44	4.46E-06	0.0130
Berber	Chinese	Indian	-4.39	5.72E-06	0.0166
Khoisan	Kalash	Indian	-4.38	5.86E-06	0.0170
Niger-Congo	Arabian	Nilo-Saharan	-4.33	7.39E-06	0.0215
Nilo-Saharan	Chinese	Southeast Asian	-4.23	1.17E-05	0.0340
Khoisan	Niger-Congo	Pygmy	-4.23	1.19E-05	0.0345
Niger-Congo	Siberian	Nilo-Saharan	-4.21	1.26E-05	0.0367
Southern European	Nilo-Saharan	Arabian	-3.98	3.42E-05	0.0995
Arabian	Native American	Indian	-3.96	3.69E-05	0.107
Japanese	Northern European	Indian	-3.90	4.84E-05	0.141
Southern European	Chinese	Berber	-3.86	5.77E-05	0.168
Niger-Congo	Siberian	Berber	-3.62	1.48E-04	0.429
Levantine-Caucasian	Siberian	Indian	-3.59	1.63E-04	0.473
Khoisan	Kalash	Nilo-Saharan	-3.56	1.86E-04	0.541
Omotic	Native American	Indian	-3.56	1.87E-04	0.542
Melanesian	Khoisan	Nilo-Saharan	-3.51	2.24E-04	0.652
Levantine-Caucasian	Omotic	Cushitic	-3.05	1.14E-03	1
Pygmy	Chinese	Cushitic	-2.79	2.64E-03	1
Levantine-Caucasian	Native American	Kalash	-2.60	4.72E-03	1
Southern European	Siberian	Northern European	-2.15	0.0158	1
Southeast Asian	Khoisan	Japanese	-2.10	0.0177	1
Chinese	Arabian	Siberian	-2.04	0.0207	1
Khoisan	Arabian	Niger-Congo	-2.03	0.0210	1
Khoisan	Cushitic	Nilo-Saharan	-1.97	0.0245	1
Khoisan	Cushitic	Pygmy	-1.88	0.0300	1
Pygmy	Siberian	Nilo-Saharan	-1.79	0.0369	1
Omotic	Arabian	Berber	-1.68	0.0469	1
Southern European	Pygmy	Arabian	-1.60	0.0546	1
Nilo-Saharan	Siberian	Japanese	-1.60	0.0546	1
Berber	Nilo-Saharan	Omotic	-1.52	0.0644	1
Melanesian	Cushitic	Berber	-1.31	0.0946	1
Niger-Congo	Native American	Kalash	-1.21	0.112	1
Nilo-Saharan	Chinese	Cushitic	-1.07	0.143	1
Pygmy	Native American	Siberian	-0.84	0.199	1
Niger-Congo	Indian	Cushitic	-0.81	0.209	1
Melanesian	Khoisan	Cushitic	-0.66	0.253	1
Khoisan	Japanese	Niger-Congo	-0.10	0.460	1
Southern European	Japanese	Northern European	0.19	0.577	1

Khoisan	Native American	Niger-Congo	0.28	0.612	1
Melanesian	Nilo-Saharan	Berber	0.41	0.658	1
Levantine-Caucasian	Japanese	Siberian	0.44	0.672	1
Southern European	Siberian	Indian	0.50	0.691	1
Khoisan	Japanese	Siberian	0.71	0.760	1
Pygmy	Indian	Niger-Congo	0.79	0.784	1
Southern European	Chinese	Southeast Asian	0.88	0.809	1
Khoisan	Indian	Niger-Congo	0.88	0.810	1
Melanesian	Northern European	Kalash	0.97	0.833	1
Pygmy	Kalash	Nilo-Saharan	0.98	0.837	1
Nilo-Saharan	Kalash	Indian	0.98	0.837	1
Niger-Congo	Chinese	Siberian	1.02	0.846	1
Southeast Asian	Niger-Congo	Japanese	1.03	0.850	1
Southeast Asian	Cushitic	Indian	1.17	0.878	1
Pygmy	Chinese	Southeast Asian	1.23	0.890	1
Khoisan	Nilo-Saharan	Pygmy	1.66	0.952	1
Melanesian	Niger-Congo	Southeast Asian	1.99	0.976	1
Pygmy	Native American	Berber	2.02	0.978	1
Southeast Asian	Native American	Chinese	2.02	0.978	1
Southern European	Chinese	Kalash	2.03	0.979	1
Southeast Asian	Berber	Indian	2.31	0.990	1
Southeast Asian	Khoisan	Niger-Congo	2.32	0.990	1
Nilo-Saharan	Siberian	Indian	2.49	0.994	1
Melanesian	Native American	Siberian	2.49	0.994	1
Southeast Asian	Nilo-Saharan	Indian	2.62	0.996	1
Chinese	Kalash	Indian	2.64	0.996	1
Melanesian	Japanese	Chinese	2.75	0.997	1
Southern European	Khoisan	Levantine-Caucasian	2.80	0.997	1
Melanesian	Pygmy	Nilo-Saharan	2.84	0.998	1
Pygmy	Native American	Japanese	2.85	0.998	1
Berber	Native American	Northern European	3.15	0.999	1
Japanese	Cushitic	Indian	3.18	0.999	1
Pygmy	Cushitic	Nilo-Saharan	3.18	0.999	1
Cushitic	Chinese	Berber	3.18	0.999	1
Japanese	Arabian	Siberian	3.37	1	1
Levantine-Caucasian	Chinese	Kalash	3.54	1	1
Arabian	Native American	Northern European	3.57	1	1
Omotic	Northern European	Cushitic	3.69	1	1
Southern European	Niger-Congo	Levantine-Caucasian	3.72	1	1
Melanesian	Pygmy	Indian	3.74	1	1
Nilo-Saharan	Siberian	Cushitic	3.74	1	1
Southeast Asian	Japanese	Chinese	3.86	1	1
Levantine-Caucasian	Khoisan	Indian	3.87	1	1
Pygmy	Kalash	Berber	3.91	1	1
Japanese	Nilo-Saharan	Indian	4.03	1	1
Khoisan	Japanese	Southeast Asian	4.16	1	1
Niger-Congo	Japanese	Chinese	4.34	1	1
Melanesian	Omotic	Indian	4.51	1	1
Khoisan	Japanese	Chinese	4.58	1	1
Southern European	Southeast Asian	Northern European	4.59	1	1
Japanese	Pygmy	Niger-Congo	4.59	1	1

Melanesian	Southern European	Arabian	4.68	1	1
Pygmy	Siberian	Japanese	4.76	1	1
Niger-Congo	Kalash	Nilo-Saharan	4.83	1	1
Japanese	Pygmy	Indian	4.91	1	1
Southeast Asian	Pygmy	Niger-Congo	4.96	1	1
Southeast Asian	Kalash	Indian	5.18	1	1
Melanesian	Arabian	Kalash	5.22	1	1
Niger-Congo	Indian	Nilo-Saharan	5.47	1	1
Southeast Asian	Pygmy	Indian	5.52	1	1
Pygmy	Native American	Niger-Congo	5.79	1	1
Southern European	Southeast Asian	Japanese	5.81	1	1
Niger-Congo	Northern European	Indian	5.82	1	1
Pygmy	Kalash	Indian	5.95	1	1
Pygmy	Indian	Cushitic	6.01	1	1
Melanesian	Kalash	Indian	6.10	1	1
Cushitic	Chinese	Southeast Asian	6.13	1	1
Levantine-Caucasian	Chinese	Southeast Asian	6.25	1	1
Berber	Chinese	Siberian	6.30	1	1
Arabian	Native American	Kalash	6.32	1	1
Khoisan	Northern European	Arabian	6.43	1	1
Melanesian	Khoisan	Berber	6.58	1	1
Pygmy	Siberian	Indian	7.07	1	1
Southern European	Niger-Congo	Indian	7.09	1	1
Japanese	Nilo-Saharan	Chinese	7.29	1	1
Southeast Asian	Northern European	Japanese	7.37	1	1
Pygmy	Chinese	Siberian	7.47	1	1
Niger-Congo	Japanese	Siberian	7.52	1	1
Berber	Native American	Indian	7.53	1	1
Southern European	Southeast Asian	Berber	7.68	1	1
Southern European	Japanese	Berber	7.69	1	1
Pygmy	Cushitic	Niger-Congo	7.77	1	1
Southeast Asian	Nilo-Saharan	Japanese	7.78	1	1
Niger-Congo	Japanese	Berber	7.83	1	1
Niger-Congo	Japanese	Southeast Asian	7.92	1	1
Berber	Japanese	Indian	7.94	1	1
Melanesian	Niger-Congo	Siberian	7.97	1	1
Khoisan	Native American	Southeast Asian	8.12	1	1
Pygmy	Siberian	Cushitic	8.13	1	1
Cushitic	Chinese	Siberian	8.14	1	1
Japanese	Pygmy	Cushitic	8.34	1	1
Khoisan	Siberian	Berber	8.38	1	1
Japanese	Pygmy	Chinese	8.41	1	1
Chinese	Kalash	Siberian	8.41	1	1
Japanese	Nilo-Saharan	Cushitic	8.59	1	1
Melanesian	Southern European	Siberian	8.71	1	1
Southeast Asian	Levantine-Caucasian	Japanese	8.77	1	1
Arabian	Siberian	Indian	8.90	1	1
Berber	Niger-Congo	Omotic	8.92	1	1
Southeast Asian	Pygmy	Japanese	9.04	1	1
Nilo-Saharan	Indian	Cushitic	9.11	1	1
Melanesian	Northern European	Siberian	9.21	1	1

Melanesian	Niger-Congo	Kalash	9.29	1	1
Southern European	Southeast Asian	Kalash	9.58	1	1
Southeast Asian	Levantine-Caucasian	Kalash	9.72	1	1
Nilo-Saharan	Native American	Kalash	9.78	1	1
Nilo-Saharan	Chinese	Siberian	9.80	1	1
Melanesian	Pygmy	Cushitic	9.86	1	1
Pygmy	Kalash	Niger-Congo	9.90	1	1
Levantine-Caucasian	Japanese	Kalash	9.91	1	1
Levantine-Caucasian	Khoisan	Arabian	9.94	1	1
Chinese	Arabian	Southeast Asian	10.04	1	1
Southeast Asian	Pygmy	Cushitic	10.06	1	1
Cushitic	Native American	Kalash	10.13	1	1
Pygmy	Siberian	Niger-Congo	10.16	1	1
Melanesian	Nilo-Saharan	Japanese	10.21	1	1
Chinese	Omotic	Southeast Asian	10.24	1	1
Japanese	Kalash	Indian	10.29	1	1
Melanesian	Southeast Asian	Japanese	10.38	1	1
Southern European	Japanese	Kalash	10.42	1	1
Southern European	Native American	Berber	10.43	1	1
Melanesian	Southern European	Japanese	10.60	1	1
Omotic	Siberian	Japanese	10.65	1	1
Southern European	Japanese	Chinese	10.74	1	1
Cushitic	Native American	Siberian	10.86	1	1
Khoisan	Cushitic	Niger-Congo	10.92	1	1
Omotic	Native American	Siberian	10.95	1	1
Southeast Asian	Nilo-Saharan	Cushitic	11.00	1	1
Khoisan	Siberian	Niger-Congo	11.06	1	1
Southeast Asian	Arabian	Japanese	11.06	1	1
Japanese	Pygmy	Siberian	11.06	1	1
Cushitic	Siberian	Indian	11.15	1	1
Khoisan	Japanese	Berber	11.39	1	1
Berber	Japanese	Siberian	11.49	1	1
Chinese	Native American	Siberian	11.57	1	1
Pygmy	Northern European	Indian	11.92	1	1
Southeast Asian	Niger-Congo	Berber	11.94	1	1
Cushitic	Siberian	Berber	12.05	1	1
Japanese	Kalash	Siberian	12.09	1	1
Japanese	Arabian	Chinese	12.19	1	1
Melanesian	Siberian	Chinese	12.20	1	1
Chinese	Northern European	Southeast Asian	12.23	1	1
Japanese	Nilo-Saharan	Siberian	12.30	1	1
Japanese	Cushitic	Siberian	12.59	1	1
Japanese	Cushitic	Berber	12.63	1	1
Niger-Congo	Native American	Southeast Asian	12.71	1	1
Japanese	Omotic	Chinese	12.80	1	1
Pygmy	Native American	Kalash	12.83	1	1
Southern European	Pygmy	Indian	12.97	1	1
Levantine-Caucasian	Japanese	Chinese	13.02	1	1
Melanesian	Southern European	Levantine-Caucasian	13.08	1	1
Cushitic	Native American	Arabian	13.34	1	1
Japanese	Nilo-Saharan	Southeast Asian	13.46	1	1

Chinese	Omotic	Indian	13.50	1	1
Melanesian	Niger-Congo	Chinese	13.56	1	1
Cushitic	Native American	Northern European	13.65	1	1
Khoisan	Kalash	Niger-Congo	13.99	1	1
Melanesian	Khoisan	Japanese	14.00	1	1
Melanesian	Northern European	Japanese	14.15	1	1
Southeast Asian	Khoisan	Berber	14.15	1	1
Melanesian	Arabian	Berber	14.37	1	1
Japanese	Northern European	Chinese	14.45	1	1
Southeast Asian	Cushitic	Berber	14.49	1	1
Cushitic	Kalash	Berber	14.54	1	1
Levantine-Caucasian	Siberian	Kalash	14.66	1	1
Southern European	Native American	Siberian	14.71	1	1
Melanesian	Chinese	Southeast Asian	14.77	1	1
Japanese	Pygmy	Southeast Asian	14.89	1	1
Japanese	Cushitic	Chinese	15.10	1	1
Niger-Congo	Native American	Chinese	15.37	1	1
Omotic	Native American	Japanese	15.83	1	1
Melanesian	Cushitic	Kalash	15.89	1	1
Melanesian	Levantine-Caucasian	Berber	16.19	1	1
Berber	Chinese	Southeast Asian	16.21	1	1
Khoisan	Northern European	Kalash	16.34	1	1
Melanesian	Nilo-Saharan	Southeast Asian	16.36	1	1
Southern European	Chinese	Arabian	16.44	1	1
Khoisan	Northern European	Levantine-Caucasian	16.59	1	1
Southern European	Nilo-Saharan	Levantine-Caucasian	16.74	1	1
Southeast Asian	Native American	Siberian	16.85	1	1
Southern European	Khoisan	Northern European	16.86	1	1
Southern European	Siberian	Kalash	17.06	1	1
Southern European	Pygmy	Levantine-Caucasian	17.07	1	1
Melanesian	Khoisan	Kalash	17.29	1	1
Berber	Japanese	Chinese	17.33	1	1
Southeast Asian	Berber	Japanese	17.35	1	1
Cushitic	Siberian	Japanese	17.47	1	1
Nilo-Saharan	Northern European	Indian	17.62	1	1
Nilo-Saharan	Siberian	Berber	17.65	1	1
Omotic	Native American	Berber	17.66	1	1
Omotic	Siberian	Indian	17.79	1	1
Berber	Native American	Kalash	17.84	1	1
Melanesian	Berber	Kalash	18.03	1	1
Chinese	Arabian	Kalash	18.05	1	1
Melanesian	Khoisan	Siberian	18.14	1	1
Melanesian	Levantine-Caucasian	Northern European	18.19	1	1
Southeast Asian	Khoisan	Chinese	18.50	1	1
Japanese	Kalash	Chinese	18.50	1	1
Southern European	Omotic	Arabian	18.52	1	1
Khoisan	Native American	Chinese	18.63	1	1
Melanesian	Levantine-Caucasian	Siberian	18.66	1	1
Southern European	Khoisan	Kalash	18.77	1	1
Chinese	Omotic	Siberian	18.88	1	1
Southeast Asian	Khoisan	Siberian	18.90	1	1

Southeast Asian	Cushitic	Japanese	18.98	1	1
Southeast Asian	Northern European	Siberian	19.02	1	1
Melanesian	Levantine-Caucasian	Japanese	19.03	1	1
Melanesian	Southern European	Southeast Asian	19.08	1	1
Southeast Asian	Omotic	Indian	19.09	1	1
Levantine-Caucasian	Cushitic	Berber	19.10	1	1
Melanesian	Cushitic	Arabian	19.13	1	1
Southeast Asian	Omotic	Japanese	19.20	1	1
Melanesian	Native American	Chinese	19.35	1	1
Melanesian	Khoisan	Southeast Asian	19.36	1	1
Melanesian	Pygmy	Niger-Congo	19.42	1	1
Japanese	Omotic	Siberian	19.59	1	1
Nilo-Saharan	Chinese	Berber	19.62	1	1
Kalash	Omotic	Berber	19.82	1	1
Melanesian	Nilo-Saharan	Siberian	19.83	1	1
Southern European	Nilo-Saharan	Indian	19.85	1	1
Arabian	Native American	Siberian	19.87	1	1
Melanesian	Pygmy	Berber	19.91	1	1
Japanese	Native American	Siberian	19.94	1	1
Southeast Asian	Kalash	Japanese	20.04	1	1
Khoisan	Indian	Berber	20.07	1	1
Pygmy	Chinese	Berber	20.11	1	1
Southern European	Niger-Congo	Northern European	20.12	1	1
Southern European	Southeast Asian	Siberian	20.13	1	1
Japanese	Omotic	Indian	20.33	1	1
Southern European	Chinese	Levantine-Caucasian	20.65	1	1
Cushitic	Native American	Japanese	20.76	1	1
Chinese	Indian	Southeast Asian	20.79	1	1
Niger-Congo	Indian	Berber	20.87	1	1
Levantine-Caucasian	Niger-Congo	Indian	21.15	1	1
Niger-Congo	Native American	Northern European	21.29	1	1
Melanesian	Nilo-Saharan	Kalash	21.29	1	1
Melanesian	Southeast Asian	Chinese	21.39	1	1
Chinese	Arabian	Berber	21.53	1	1
Japanese	Indian	Chinese	21.57	1	1
Melanesian	Native American	Southeast Asian	21.60	1	1
Omotic	Native American	Kalash	21.73	1	1
Niger-Congo	Siberian	Chinese	21.79	1	1
Khoisan	Siberian	Southeast Asian	22.01	1	1
Berber	Siberian	Indian	22.13	1	1
Southeast Asian	Niger-Congo	Chinese	22.40	1	1
Southern European	Siberian	Japanese	22.50	1	1
Melanesian	Niger-Congo	Pygmy	22.67	1	1
Arabian	Native American	Berber	22.68	1	1
Omotic	Native American	Cushitic	22.70	1	1
Kalash	Omotic	Indian	22.74	1	1
Southern European	Siberian	Berber	22.78	1	1
Southern European	Japanese	Southeast Asian	22.79	1	1
Southeast Asian	Arabian	Kalash	22.84	1	1
Kalash	Omotic	Cushitic	22.88	1	1
Levantine-Caucasian	Pygmy	Indian	22.91	1	1

Japanese	Omotic	Southeast Asian	23.22	1	1
Levantine-Caucasian	Khoisan	Kalash	23.24	1	1
Japanese	Native American	Chinese	23.36	1	1
Melanesian	Khoisan	Niger-Congo	23.41	1	1
Melanesian	Japanese	Southeast Asian	23.59	1	1
Berber	Native American	Siberian	23.63	1	1
Khoisan	Omotic	Pygmy	23.67	1	1
Southern European	Native American	Levantine-Caucasian	23.69	1	1
Levantine-Caucasian	Siberian	Japanese	23.71	1	1
Indian	Siberian	Japanese	23.72	1	1
Melanesian	Arabian	Japanese	23.75	1	1
Nilo-Saharan	Native American	Southeast Asian	23.76	1	1
Khoisan	Chinese	Kalash	23.79	1	1
Chinese	Kalash	Southeast Asian	23.80	1	1
Japanese	Arabian	Kalash	23.82	1	1
Japanese	Cushitic	Southeast Asian	23.86	1	1
Melanesian	Arabian	Siberian	24.00	1	1
Cushitic	Northern European	Arabian	24.02	1	1
Khoisan	Siberian	Chinese	24.12	1	1
Pygmy	Siberian	Berber	24.22	1	1
Berber	Siberian	Japanese	24.38	1	1
Melanesian	Cushitic	Japanese	24.53	1	1
Melanesian	Pygmy	Japanese	24.67	1	1
Khoisan	Siberian	Kalash	24.67	1	1
Levantine-Caucasian	Chinese	Northern European	24.77	1	1
Niger-Congo	Northern European	Levantine-Caucasian	24.84	1	1
Levantine-Caucasian	Niger-Congo	Arabian	25.11	1	1
Pygmy	Native American	Southeast Asian	25.24	1	1
Melanesian	Levantine-Caucasian	Southeast Asian	25.45	1	1
Melanesian	Omotic	Berber	25.46	1	1
Southeast Asian	Pygmy	Chinese	25.51	1	1
Cushitic	Kalash	Levantine-Caucasian	25.53	1	1
Arabian	Siberian	Japanese	25.62	1	1
Levantine-Caucasian	Siberian	Northern European	25.77	1	1
Melanesian	Northern European	Southeast Asian	25.83	1	1
Nilo-Saharan	Cushitic	Omotic	25.96	1	1
Levantine-Caucasian	Native American	Siberian	26.06	1	1
Cushitic	Native American	Levantine-Caucasian	26.14	1	1
Berber	Omotic	Cushitic	26.16	1	1
Melanesian	Cushitic	Siberian	26.23	1	1
Niger-Congo	Northern European	Arabian	26.53	1	1
Southeast Asian	Levantine-Caucasian	Siberian	26.70	1	1
Nilo-Saharan	Siberian	Chinese	26.75	1	1
Kalash	Native American	Indian	26.90	1	1
Melanesian	Levantine-Caucasian	Arabian	26.97	1	1
Southeast Asian	Arabian	Berber	27.09	1	1
Southern European	Pygmy	Northern European	27.14	1	1
Southern European	Native American	Arabian	27.16	1	1
Levantine-Caucasian	Japanese	Southeast Asian	27.21	1	1
Southern European	Southeast Asian	Arabian	27.22	1	1
Berber	Niger-Congo	Nilo-Saharan	27.29	1	1

Kalash	Siberian	Indian	27.33	1	1
Melanesian	Omotic	Cushitic	27.43	1	1
Arabian	Siberian	Kalash	27.44	1	1
Pygmy	Northern European	Arabian	27.55	1	1
Khoisan	Native American	Arabian	27.61	1	1
Cushitic	Kalash	Arabian	27.83	1	1
Melanesian	Siberian	Southeast Asian	27.84	1	1
Niger-Congo	Siberian	Southeast Asian	27.97	1	1
Levantine-Caucasian	Pygmy	Arabian	28.02	1	1
Nilo-Saharan	Native American	Chinese	28.09	1	1
Melanesian	Cushitic	Southeast Asian	28.12	1	1
Southeast Asian	Northern European	Chinese	28.15	1	1
Niger-Congo	Cushitic	Nilo-Saharan	28.18	1	1
Southern European	Southeast Asian	Chinese	28.26	1	1
Pygmy	Northern European	Kalash	28.26	1	1
Japanese	Arabian	Southeast Asian	28.26	1	1
Japanese	Nilo-Saharan	Berber	28.36	1	1
Melanesian	Berber	Arabian	28.64	1	1
Southeast Asian	Nilo-Saharan	Chinese	28.65	1	1
Melanesian	Pygmy	Southeast Asian	28.69	1	1
Japanese	Arabian	Berber	28.80	1	1
Chinese	Northern European	Kalash	28.90	1	1
Southeast Asian	Arabian	Siberian	29.05	1	1
Melanesian	Pygmy	Siberian	29.06	1	1
Southeast Asian	Niger-Congo	Siberian	29.09	1	1
Southern European	Japanese	Arabian	29.12	1	1
Khoisan	Native American	Northern European	29.13	1	1
Southeast Asian	Pygmy	Siberian	29.17	1	1
Melanesian	Nilo-Saharan	Chinese	29.39	1	1
Pygmy	Siberian	Chinese	29.49	1	1
Southern European	Pygmy	Kalash	29.54	1	1
Chinese	Indian	Siberian	29.56	1	1
Japanese	Pygmy	Berber	29.64	1	1
Melanesian	Omotic	Japanese	29.90	1	1
Niger-Congo	Omotic	Nilo-Saharan	30.05	1	1
Cushitic	Indian	Berber	30.35	1	1
Melanesian	Pygmy	Kalash	30.65	1	1
Native American	Siberian	Japanese	30.71	1	1
Southeast Asian	Chinese	Japanese	30.77	1	1
Southeast Asian	Pygmy	Berber	30.79	1	1
Niger-Congo	Kalash	Levantine-Caucasian	30.79	1	1
Southeast Asian	Northern European	Kalash	30.83	1	1
Southeast Asian	Indian	Japanese	30.92	1	1
Pygmy	Northern European	Levantine-Caucasian	30.97	1	1
Melanesian	Northern European	Berber	31.11	1	1
Southeast Asian	Nilo-Saharan	Berber	31.25	1	1
Southeast Asian	Levantine-Caucasian	Chinese	31.47	1	1
Pygmy	Native American	Chinese	31.63	1	1
Melanesian	Berber	Siberian	31.67	1	1
Japanese	Indian	Siberian	31.67	1	1
Nilo-Saharan	Northern European	Levantine-Caucasian	31.80	1	1

Berber	Kalash	Levantine-Caucasian	31.80	1	1
Southeast Asian	Arabian	Chinese	31.83	1	1
Southeast Asian	Khoisan	Kalash	31.86	1	1
Levantine-Caucasian	Nilo-Saharan	Indian	32.04	1	1
Chinese	Omotic	Cushitic	32.09	1	1
Cushitic	Chinese	Arabian	32.15	1	1
Melanesian	Berber	Japanese	32.43	1	1
Khoisan	Japanese	Kalash	32.47	1	1
Niger-Congo	Cushitic	Omotic	32.48	1	1
Khoisan	Arabian	Indian	32.50	1	1
Melanesian	Arabian	Southeast Asian	32.51	1	1
Southern European	Southeast Asian	Levantine-Caucasian	32.65	1	1
Southern European	Nilo-Saharan	Northern European	32.95	1	1
Southern European	Cushitic	Arabian	33.02	1	1
Melanesian	Niger-Congo	Arabian	33.07	1	1
Berber	Native American	Levantine-Caucasian	33.09	1	1
Melanesian	Japanese	Siberian	33.15	1	1
Kalash	Siberian	Japanese	33.18	1	1
Berber	Japanese	Southeast Asian	33.25	1	1
Arabian	Native American	Levantine-Caucasian	33.34	1	1
Melanesian	Arabian	Levantine-Caucasian	33.35	1	1
Nilo-Saharan	Siberian	Southeast Asian	33.39	1	1
Northern European	Siberian	Indian	33.39	1	1
Pygmy	Siberian	Southeast Asian	33.44	1	1
Nilo-Saharan	Northern European	Arabian	33.48	1	1
Melanesian	Omotic	Southeast Asian	33.65	1	1
Japanese	Northern European	Southeast Asian	33.71	1	1
Southern European	Japanese	Levantine-Caucasian	33.97	1	1
Niger-Congo	Native American	Arabian	34.09	1	1
Cushitic	Siberian	Arabian	34.16	1	1
Melanesian	Omotic	Kalash	34.17	1	1
Omotic	Siberian	Cushitic	34.19	1	1
Indian	Native American	Siberian	34.42	1	1
Melanesian	Southern European	Chinese	34.45	1	1
Japanese	Northern European	Kalash	34.57	1	1
Southeast Asian	Omotic	Chinese	34.58	1	1
Levantine-Caucasian	Japanese	Northern European	34.60	1	1
Melanesian	Chinese	Siberian	34.84	1	1
Levantine-Caucasian	Chinese	Berber	34.89	1	1
Cushitic	Northern European	Levantine-Caucasian	34.89	1	1
Melanesian	Arabian	Northern European	34.95	1	1
Southern European	Native American	Japanese	35.04	1	1
Khoisan	Kalash	Arabian	35.10	1	1
Berber	Chinese	Kalash	35.20	1	1
Pygmy	Indian	Berber	35.29	1	1
Levantine-Caucasian	Nilo-Saharan	Arabian	35.41	1	1
Berber	Native American	Arabian	35.41	1	1
Melanesian	Omotic	Siberian	35.44	1	1
Southeast Asian	Nilo-Saharan	Siberian	35.50	1	1
Omotic	Siberian	Berber	35.66	1	1
Arabian	Siberian	Berber	35.78	1	1

Northern European	Siberian	Japanese	35.85	1	1
Southeast Asian	Berber	Siberian	35.96	1	1
Nilo-Saharan	Indian	Berber	35.97	1	1
Omotic	Siberian	Chinese	36.31	1	1
Cushitic	Siberian	Kalash	36.32	1	1
Southeast Asian	Berber	Chinese	36.39	1	1
Khoisan	Nilo-Saharan	Niger-Congo	36.57	1	1
Melanesian	Northern European	Chinese	36.69	1	1
Southeast Asian	Kalash	Siberian	36.70	1	1
Niger-Congo	Siberian	Kalash	36.83	1	1
Levantine-Caucasian	Pygmy	Kalash	37.27	1	1
Melanesian	Berber	Northern European	37.29	1	1
Berber	Siberian	Kalash	37.32	1	1
Melanesian	Cushitic	Levantine-Caucasian	37.39	1	1
Cushitic	Chinese	Kalash	37.39	1	1
Japanese	Omotic	Cushitic	37.51	1	1
Arabian	Native American	Japanese	37.54	1	1
Khoisan	Kalash	Levantine-Caucasian	37.68	1	1
Southeast Asian	Kalash	Chinese	37.76	1	1
Berber	Siberian	Northern European	37.79	1	1
Pygmy	Siberian	Kalash	37.80	1	1
Southern European	Siberian	Arabian	38.07	1	1
Melanesian	Kalash	Siberian	38.18	1	1
Melanesian	Khoisan	Chinese	38.57	1	1
Southeast Asian	Cushitic	Chinese	38.58	1	1
Melanesian	Berber	Levantine-Caucasian	38.71	1	1
Niger-Congo	Kalash	Arabian	38.87	1	1
Melanesian	Berber	Southeast Asian	38.87	1	1
Southeast Asian	Omotic	Cushitic	38.90	1	1
Southeast Asian	Berber	Kalash	38.91	1	1
Southeast Asian	Levantine-Caucasian	Northern European	39.01	1	1
Omotic	Northern European	Levantine-Caucasian	39.03	1	1
Japanese	Kalash	Southeast Asian	39.12	1	1
Berber	Native American	Japanese	39.16	1	1
Nilo-Saharan	Kalash	Levantine-Caucasian	39.35	1	1
Berber	Japanese	Kalash	39.51	1	1
Southern European	Niger-Congo	Kalash	39.62	1	1
Southeast Asian	Cushitic	Siberian	39.80	1	1
Chinese	Omotic	Berber	40.01	1	1
Melanesian	Levantine-Caucasian	Chinese	40.13	1	1
Kalash	Native American	Siberian	40.31	1	1
Japanese	Indian	Southeast Asian	40.32	1	1
Omotic	Native American	Southeast Asian	40.62	1	1
Niger-Congo	Northern European	Kalash	40.63	1	1
Indian	Omotic	Cushitic	40.69	1	1
Melanesian	Nilo-Saharan	Niger-Congo	40.76	1	1
Nilo-Saharan	Native American	Northern European	40.80	1	1
Japanese	Cushitic	Kalash	40.97	1	1
Japanese	Cushitic	Arabian	41.01	1	1
Melanesian	Indian	Japanese	41.20	1	1
Melanesian	Kalash	Japanese	41.59	1	1

Southern European	Omotic	Levantine-Caucasian	41.62	1	1
Native American	Northern European	Kalash	41.87	1	1
Khoisan	Nilo-Saharan	Omotic	41.91	1	1
Omotic	Northern European	Arabian	41.99	1	1
Nilo-Saharan	Siberian	Kalash	42.14	1	1
Levantine-Caucasian	Native American	Berber	42.37	1	1
Pygmy	Chinese	Kalash	42.41	1	1
Southeast Asian	Cushitic	Kalash	42.48	1	1
Southern European	Siberian	Levantine-Caucasian	42.50	1	1
Berber	Chinese	Arabian	42.51	1	1
Pygmy	Kalash	Levantine-Caucasian	42.55	1	1
Southeast Asian	Levantine-Caucasian	Berber	42.57	1	1
Omotic	Siberian	Southeast Asian	42.68	1	1
Southeast Asian	Omotic	Siberian	42.70	1	1
Cushitic	Arabian	Berber	42.80	1	1
Native American	Northern European	Siberian	42.95	1	1
Arabian	Siberian	Northern European	42.95	1	1
Indian	Native American	Japanese	43.13	1	1
Nilo-Saharan	Northern European	Kalash	43.15	1	1
Levantine-Caucasian	Japanese	Berber	43.17	1	1
Native American	Northern European	Indian	43.17	1	1
Melanesian	Nilo-Saharan	Pygmy	43.37	1	1
Pygmy	Arabian	Indian	43.43	1	1
Melanesian	Pygmy	Khoisan	43.48	1	1
Southeast Asian	Cushitic	Arabian	44.17	1	1
Japanese	Omotic	Berber	44.20	1	1
Khoisan	Native American	Levantine-Caucasian	44.28	1	1
Melanesian	Northern European	Levantine-Caucasian	44.29	1	1
Levantine-Caucasian	Native American	Japanese	44.50	1	1
Niger-Congo	Native American	Levantine-Caucasian	44.71	1	1
Southern European	Nilo-Saharan	Kalash	44.84	1	1
Kalash	Omotic	Levantine-Caucasian	44.85	1	1
Melanesian	Cushitic	Omotic	44.93	1	1
Nilo-Saharan	Chinese	Niger-Congo	44.96	1	1
Cushitic	Kalash	Indian	45.03	1	1
Nilo-Saharan	Native American	Niger-Congo	45.10	1	1
Pygmy	Kalash	Arabian	45.15	1	1
Melanesian	Niger-Congo	Northern European	45.30	1	1
Omotic	Native American	Chinese	45.31	1	1
Khoisan	Arabian	Kalash	45.40	1	1
Chinese	Arabian	Northern European	45.59	1	1
Khoisan	Indian	Kalash	45.79	1	1
Niger-Congo	Native American	Pygmy	45.87	1	1
Japanese	Siberian	Chinese	45.89	1	1
Berber	Chinese	Northern European	45.93	1	1
Melanesian	Pygmy	Chinese	46.02	1	1
Pygmy	Nilo-Saharan	Niger-Congo	46.16	1	1
Pygmy	Native American	Northern European	46.18	1	1
Southeast Asian	Omotic	Berber	46.23	1	1
Northern European	Siberian	Kalash	46.36	1	1
Southern European	Nilo-Saharan	Niger-Congo	46.38	1	1

Melanesian	Cushitic	Chinese	46.44	1	1
Southeast Asian	Berber	Arabian	46.50	1	1
Omotic	Native American	Northern European	46.52	1	1
Levantine-Caucasian	Omotic	Arabian	46.59	1	1
Cushitic	Northern European	Southern European	46.63	1	1
Japanese	Pygmy	Kalash	46.63	1	1
Nilo-Saharan	Kalash	Arabian	46.66	1	1
Melanesian	Cushitic	Northern European	46.81	1	1
Melanesian	Indian	Southeast Asian	46.87	1	1
Melanesian	Northern European	Arabian	47.11	1	1
Berber	Siberian	Arabian	47.23	1	1
Southeast Asian	Pygmy	Kalash	47.30	1	1
Chinese	Arabian	Levantine-Caucasian	47.56	1	1
Melanesian	Omotic	Chinese	47.62	1	1
Nilo-Saharan	Native American	Arabian	47.62	1	1
Cushitic	Siberian	Chinese	47.67	1	1
Niger-Congo	Arabian	Indian	47.70	1	1
Melanesian	Southeast Asian	Siberian	47.84	1	1
Khoisan	Northern European	Southern European	47.85	1	1
Omotic	Northern European	Indian	47.86	1	1
Omotic	Siberian	Kalash	47.99	1	1
Melanesian	Indian	Siberian	48.46	1	1
Levantine-Caucasian	Chinese	Arabian	48.56	1	1
Melanesian	Nilo-Saharan	Arabian	48.57	1	1
Berber	Japanese	Arabian	48.63	1	1
Melanesian	Kalash	Southeast Asian	48.82	1	1
Berber	Native American	Southern European	48.89	1	1
Melanesian	Khoisan	Arabian	48.97	1	1
Niger-Congo	Chinese	Kalash	49.10	1	1
Cushitic	Native American	Southeast Asian	49.21	1	1
Levantine-Caucasian	Khoisan	Northern European	49.38	1	1
Levantine-Caucasian	Niger-Congo	Kalash	49.50	1	1
Southern European	Kalash	Levantine-Caucasian	49.55	1	1
Levantine-Caucasian	Cushitic	Arabian	49.65	1	1
Pygmy	Omotic	Nilo-Saharan	49.71	1	1
Indian	Omotic	Berber	49.74	1	1
Melanesian	Niger-Congo	Levantine-Caucasian	49.82	1	1
Berber	Chinese	Levantine-Caucasian	49.92	1	1
Southern European	Kalash	Northern European	50.04	1	1
Melanesian	Arabian	Chinese	50.10	1	1
Khoisan	Arabian	Levantine-Caucasian	50.10	1	1
Kalash	Omotic	Arabian	50.34	1	1
Melanesian	Berber	Southern European	50.34	1	1
Pygmy	Native American	Arabian	50.60	1	1
Niger-Congo	Kalash	Northern European	50.62	1	1
Berber	Kalash	Arabian	50.64	1	1
Kalash	Arabian	Levantine-Caucasian	50.82	1	1
Southern European	Indian	Kalash	51.05	1	1
Khoisan	Kalash	Northern European	51.08	1	1
Nilo-Saharan	Northern European	Niger-Congo	51.45	1	1
Levantine-Caucasian	Siberian	Berber	51.50	1	1

Berber	Siberian	Levantine-Caucasian	51.54	1	1
Berber	Japanese	Northern European	51.67	1	1
Japanese	Nilo-Saharan	Niger-Congo	51.85	1	1
Southeast Asian	Niger-Congo	Kalash	52.01	1	1
Niger-Congo	Japanese	Kalash	52.02	1	1
Cushitic	Indian	Arabian	52.06	1	1
Southeast Asian	Indian	Chinese	52.17	1	1
Southeast Asian	Arabian	Levantine-Caucasian	52.26	1	1
Melanesian	Arabian	Southern European	52.31	1	1
Japanese	Arabian	Northern European	52.34	1	1
Levantine-Caucasian	Nilo-Saharan	Kalash	52.54	1	1
Melanesian	Native American	Indian	52.61	1	1
Southeast Asian	Berber	Northern European	52.75	1	1
Southern European	Indian	Northern European	52.78	1	1
Southeast Asian	Nilo-Saharan	Niger-Congo	52.82	1	1
Japanese	Arabian	Levantine-Caucasian	52.88	1	1
Nilo-Saharan	Siberian	Niger-Congo	52.97	1	1
Arabian	Siberian	Levantine-Caucasian	53.14	1	1
Cushitic	Siberian	Southeast Asian	53.38	1	1
Pygmy	Arabian	Kalash	53.42	1	1
Melanesian	Indian	Kalash	53.77	1	1
Omotic	Native American	Arabian	53.80	1	1
Southeast Asian	Berber	Levantine-Caucasian	53.89	1	1
Berber	Indian	Levantine-Caucasian	54.03	1	1
Southeast Asian	Arabian	Northern European	54.31	1	1
Levantine-Caucasian	Nilo-Saharan	Niger-Congo	54.84	1	1
Cushitic	Siberian	Levantine-Caucasian	55.02	1	1
Pygmy	Native American	Khoisan	55.04	1	1
Berber	Japanese	Levantine-Caucasian	55.05	1	1
Cushitic	Native American	Southern European	55.14	1	1
Levantine-Caucasian	Native American	Arabian	55.21	1	1
Chinese	Native American	Southeast Asian	55.23	1	1
Berber	Kalash	Northern European	55.32	1	1
Pygmy	Arabian	Levantine-Caucasian	55.41	1	1
Melanesian	Berber	Chinese	55.57	1	1
Omotic	Northern European	Kalash	55.57	1	1
Melanesian	Levantine-Caucasian	Southern European	55.62	1	1
Melanesian	Southern European	Cushitic	55.74	1	1
Melanesian	Berber	Cushitic	55.83	1	1
Melanesian	Omotic	Nilo-Saharan	55.84	1	1
Berber	Kalash	Southern European	56.05	1	1
Indian	Native American	Kalash	56.18	1	1
Southeast Asian	Levantine-Caucasian	Arabian	56.24	1	1
Niger-Congo	Arabian	Levantine-Caucasian	56.33	1	1
Southern European	Indian	Berber	56.59	1	1
Nilo-Saharan	Chinese	Kalash	56.84	1	1
Pygmy	Indian	Kalash	57.22	1	1
Cushitic	Chinese	Levantine-Caucasian	57.24	1	1
Japanese	Nilo-Saharan	Kalash	57.32	1	1
Southern European	Indian	Levantine-Caucasian	57.41	1	1
Nilo-Saharan	Arabian	Indian	57.54	1	1

Nilo-Saharan	Kalash	Niger-Congo	57.62	1	1
Cushitic	Native American	Chinese	57.63	1	1
Levantine-Caucasian	Japanese	Arabian	57.76	1	1
Melanesian	Omotic	Arabian	57.78	1	1
Khoisan	Niger-Congo	Nilo-Saharan	57.84	1	1
Berber	Indian	Arabian	57.87	1	1
Pygmy	Kalash	Northern European	57.87	1	1
Berber	Siberian	Chinese	58.42	1	1
Southeast Asian	Nilo-Saharan	Kalash	58.47	1	1
Southern European	Pygmy	Khoisan	58.58	1	1
Cushitic	Siberian	Northern European	58.66	1	1
Nilo-Saharan	Kalash	Northern European	58.98	1	1
Arabian	Siberian	Chinese	58.99	1	1
Kalash	Native American	Japanese	58.99	1	1
Arabian	Native American	Southern European	59.01	1	1
Khoisan	Siberian	Arabian	59.07	1	1
Southern European	Siberian	Chinese	59.16	1	1
Southern European	Omotic	Northern European	59.17	1	1
Melanesian	Cushitic	Southern European	59.23	1	1
Melanesian	Arabian	Cushitic	59.35	1	1
Pygmy	Chinese	Khoisan	59.35	1	1
Nilo-Saharan	Native American	Levantine-Caucasian	59.38	1	1
Indian	Siberian	Chinese	59.41	1	1
Levantine-Caucasian	Siberian	Chinese	59.44	1	1
Melanesian	Pygmy	Arabian	59.46	1	1
Chinese	Omotic	Kalash	59.72	1	1
Southern European	Omotic	Indian	59.74	1	1
Levantine-Caucasian	Indian	Kalash	59.84	1	1
Berber	Nilo-Saharan	Niger-Congo	59.97	1	1
Nilo-Saharan	Arabian	Niger-Congo	59.99	1	1
Southeast Asian	Omotic	Kalash	60.35	1	1
Japanese	Omotic	Kalash	60.68	1	1
Southeast Asian	Pygmy	Khoisan	61.13	1	1
Niger-Congo	Siberian	Pygmy	61.15	1	1
Nilo-Saharan	Indian	Niger-Congo	61.41	1	1
Khoisan	Omotic	Nilo-Saharan	61.65	1	1
Melanesian	Native American	Kalash	61.84	1	1
Khoisan	Chinese	Arabian	61.85	1	1
Levantine-Caucasian	Pygmy	Northern European	61.87	1	1
Pygmy	Native American	Levantine-Caucasian	62.04	1	1
Indian	Arabian	Kalash	62.08	1	1
Japanese	Pygmy	Khoisan	62.11	1	1
Omotic	Native American	Levantine-Caucasian	62.20	1	1
Levantine-Caucasian	Siberian	Arabian	62.36	1	1
Nilo-Saharan	Native American	Pygmy	62.48	1	1
Niger-Congo	Chinese	Pygmy	62.72	1	1
Pygmy	Northern European	Southern European	62.90	1	1
Berber	Indian	Kalash	63.04	1	1
Berber	Siberian	Southern European	63.22	1	1
Pygmy	Siberian	Khoisan	63.59	1	1
Southeast Asian	Niger-Congo	Pygmy	63.79	1	1

Japanese	Cushitic	Levantine-Caucasian	63.86	1	1
Cushitic	Native American	Omotic	63.97	1	1
Niger-Congo	Northern European	Southern European	64.00	1	1
Kalash	Omotic	Northern European	64.01	1	1
Southern European	Siberian	Southeast Asian	64.19	1	1
Indian	Northern European	Kalash	64.40	1	1
Omotic	Native American	Nilo-Saharan	64.53	1	1
Southeast Asian	Indian	Siberian	64.58	1	1
Arabian	Northern European	Berber	64.69	1	1
Levantine-Caucasian	Pygmy	Khoisan	64.72	1	1
Niger-Congo	Japanese	Pygmy	64.74	1	1
Pygmy	Arabian	Khoisan	64.78	1	1
Nilo-Saharan	Arabian	Levantine-Caucasian	64.88	1	1
Melanesian	Kalash	Chinese	64.90	1	1
Cushitic	Kalash	Northern European	65.03	1	1
Melanesian	Nilo-Saharan	Northern European	65.05	1	1
Melanesian	Nilo-Saharan	Levantine-Caucasian	65.20	1	1
Levantine-Caucasian	Omotic	Indian	65.41	1	1
Pygmy	Northern European	Khoisan	65.55	1	1
Chinese	Northern European	Berber	65.83	1	1
Levantine-Caucasian	Niger-Congo	Northern European	65.90	1	1
Pygmy	Omotic	Niger-Congo	66.07	1	1
Southern European	Omotic	Kalash	66.34	1	1
Pygmy	Kalash	Khoisan	66.44	1	1
Berber	Siberian	Southeast Asian	66.56	1	1
Melanesian	Northern European	Southern European	66.89	1	1
Southeast Asian	Cushitic	Levantine-Caucasian	66.96	1	1
Cushitic	Indian	Levantine-Caucasian	66.97	1	1
Kalash	Siberian	Chinese	66.98	1	1
Niger-Congo	Arabian	Kalash	66.98	1	1
Cushitic	Indian	Kalash	66.99	1	1
Berber	Chinese	Southern European	67.10	1	1
Arabian	Siberian	Southeast Asian	67.11	1	1
Southeast Asian	Northern European	Berber	67.15	1	1
Cushitic	Kalash	Southern European	67.20	1	1
Southern European	Indian	Arabian	67.34	1	1
Melanesian	Indian	Chinese	67.55	1	1
Levantine-Caucasian	Siberian	Southeast Asian	67.79	1	1
Levantine-Caucasian	Omotic	Kalash	67.84	1	1
Southeast Asian	Berber	Southern European	68.23	1	1
Chinese	Omotic	Nilo-Saharan	68.86	1	1
Berber	Northern European	Southern European	68.98	1	1
Indian	Arabian	Levantine-Caucasian	69.24	1	1
Omotic	Northern European	Southern European	69.30	1	1
Niger-Congo	Kalash	Pygmy	69.36	1	1
Levantine-Caucasian	Khoisan	Southern European	69.37	1	1
Cushitic	Chinese	Omotic	69.40	1	1
Indian	Arabian	Berber	69.41	1	1
Khoisan	Niger-Congo	Omotic	69.42	1	1
Niger-Congo	Indian	Kalash	69.57	1	1
Melanesian	Levantine-Caucasian	Cushitic	69.61	1	1

Native American	Siberian	Chinese	69.65	1	1
Southern European	Native American	Southeast Asian	70.03	1	1
Berber	Japanese	Southern European	70.07	1	1
Melanesian	Khoisan	Levantine-Caucasian	70.23	1	1
Southern European	Kalash	Berber	70.42	1	1
Berber	Native American	Southeast Asian	70.51	1	1
Berber	Kalash	Indian	71.00	1	1
Japanese	Native American	Southeast Asian	71.00	1	1
Nilo-Saharan	Arabian	Kalash	71.11	1	1
Indian	Siberian	Southeast Asian	71.14	1	1
Melanesian	Omotic	Levantine-Caucasian	71.18	1	1
Melanesian	Khoisan	Northern European	71.52	1	1
Indian	Omotic	Kalash	71.62	1	1
Nilo-Saharan	Northern European	Southern European	71.65	1	1
Pygmy	Indian	Khoisan	71.70	1	1
Japanese	Omotic	Nilo-Saharan	71.88	1	1
Kalash	Arabian	Berber	72.03	1	1
Southeast Asian	Berber	Cushitic	72.11	1	1
Arabian	Northern European	Southern European	72.18	1	1
Omotic	Siberian	Nilo-Saharan	72.30	1	1
Niger-Congo	Siberian	Arabian	72.31	1	1
Khoisan	Japanese	Arabian	72.34	1	1
Southeast Asian	Omotic	Nilo-Saharan	72.35	1	1
Nilo-Saharan	Indian	Kalash	72.48	1	1
Japanese	Northern European	Berber	72.52	1	1
Arabian	Native American	Southeast Asian	72.53	1	1
Melanesian	Omotic	Pygmy	72.85	1	1
Native American	Northern European	Japanese	73.29	1	1
Pygmy	Nilo-Saharan	Omotic	73.34	1	1
Pygmy	Siberian	Arabian	73.38	1	1
Chinese	Arabian	Southern European	73.43	1	1
Kalash	Native American	Northern European	73.50	1	1
Berber	Khoisan	Arabian	73.60	1	1
Cushitic	Chinese	Northern European	73.65	1	1
Melanesian	Omotic	Northern European	73.72	1	1
Berber	Chinese	Cushitic	73.76	1	1
Khoisan	Arabian	Southern European	74.00	1	1
Southern European	Arabian	Berber	74.32	1	1
Southeast Asian	Khoisan	Arabian	74.43	1	1
Southeast Asian	Cushitic	Omotic	74.48	1	1
Omotic	Siberian	Arabian	74.49	1	1
Japanese	Cushitic	Omotic	74.61	1	1
Berber	Indian	Northern European	74.74	1	1
Kalash	Siberian	Southeast Asian	75.16	1	1
Southern European	Kalash	Arabian	75.26	1	1
Berber	Native American	Cushitic	75.32	1	1
Khoisan	Siberian	Northern European	75.39	1	1
Southeast Asian	Arabian	Southern European	75.59	1	1
Arabian	Siberian	Southern European	75.74	1	1
Melanesian	Pygmy	Levantine-Caucasian	75.81	1	1
Northern European	Siberian	Chinese	75.90	1	1

Khoisan	Indian	Arabian	76.10	1	1
Khoisan	Kalash	Southern European	76.37	1	1
Melanesian	Pygmy	Northern European	76.94	1	1
Arabian	Northern European	Levantine-Caucasian	76.98	1	1
Melanesian	Kalash	Berber	77.07	1	1
Levantine-Caucasian	Nilo-Saharan	Northern European	77.17	1	1
Nilo-Saharan	Siberian	Pygmy	77.33	1	1
Japanese	Arabian	Southern European	77.35	1	1
Japanese	Cushitic	Northern European	77.51	1	1
Berber	Indian	Southern European	77.55	1	1
Indian	Native American	Southeast Asian	77.57	1	1
Omotic	Arabian	Levantine-Caucasian	77.61	1	1
Berber	Japanese	Cushitic	77.83	1	1
Southern European	Levantine-Caucasian	Berber	78.03	1	1
Khoisan	Arabian	Northern European	78.19	1	1
Berber	Native American	Chinese	78.44	1	1
Kalash	Arabian	Indian	78.46	1	1
Niger-Congo	Siberian	Northern European	78.53	1	1
Cushitic	Siberian	Omotic	79.05	1	1
Nilo-Saharan	Siberian	Arabian	79.09	1	1
Nilo-Saharan	Chinese	Pygmy	79.32	1	1
Melanesian	Kalash	Northern European	79.39	1	1
Southeast Asian	Nilo-Saharan	Pygmy	79.46	1	1
Southern European	Niger-Congo	Pygmy	79.48	1	1
Melanesian	Siberian	Indian	79.52	1	1
Khoisan	Siberian	Levantine-Caucasian	79.63	1	1
Niger-Congo	Kalash	Southern European	79.82	1	1
Nilo-Saharan	Cushitic	Niger-Congo	79.87	1	1
Levantine-Caucasian	Native American	Southeast Asian	80.10	1	1
Berber	Pygmy	Khoisan	80.12	1	1
Melanesian	Berber	Omotic	80.62	1	1
Japanese	Nilo-Saharan	Pygmy	80.68	1	1
Melanesian	Niger-Congo	Southern European	80.71	1	1
Khoisan	Native American	Southern European	80.72	1	1
Pygmy	Arabian	Southern European	80.74	1	1
Niger-Congo	Arabian	Pygmy	81.06	1	1
Southeast Asian	Arabian	Cushitic	81.29	1	1
Niger-Congo	Northern European	Pygmy	81.47	1	1
Kalash	Arabian	Northern European	81.64	1	1
Southeast Asian	Northern European	Levantine-Caucasian	81.67	1	1
Pygmy	Arabian	Northern European	81.73	1	1
Arabian	Native American	Chinese	81.76	1	1
Levantine-Caucasian	Pygmy	Southern European	81.97	1	1
Indian	Siberian	Kalash	82.03	1	1
Southern European	Native American	Chinese	82.03	1	1
Pygmy	Kalash	Southern European	82.31	1	1
Cushitic	Siberian	Southern European	82.32	1	1
Pygmy	Chinese	Arabian	82.35	1	1
Berber	Siberian	Cushitic	82.67	1	1
Pygmy	Siberian	Northern European	82.82	1	1
Chinese	Arabian	Cushitic	82.83	1	1

Southeast Asian	Cushitic	Northern European	82.96	1	1
Chinese	Northern European	Levantine-Caucasian	83.18	1	1
Melanesian	Kalash	Levantine-Caucasian	83.25	1	1
Khoisan	Kalash	Siberian	83.55	1	1
Levantine-Caucasian	Arabian	Berber	83.63	1	1
Omotic	Siberian	Northern European	84.14	1	1
Southern European	Cushitic	Levantine-Caucasian	84.25	1	1
Northern European	Siberian	Southeast Asian	84.25	1	1
Nilo-Saharan	Kalash	Pygmy	84.31	1	1
Levantine-Caucasian	Niger-Congo	Pygmy	84.38	1	1
Niger-Congo	Pygmy	Nilo-Saharan	84.63	1	1
Southern European	Kalash	Indian	84.65	1	1
Niger-Congo	Indian	Pygmy	85.07	1	1
Kalash	Omotic	Southern European	85.08	1	1
Berber	Northern European	Levantine-Caucasian	85.57	1	1
Berber	Khoisan	Indian	85.67	1	1
Levantine-Caucasian	Native American	Southern European	86.06	1	1
Omotic	Arabian	Kalash	86.13	1	1
Omotic	Arabian	Indian	86.27	1	1
Cushitic	Northern European	Kalash	86.35	1	1
Indian	Native American	Chinese	86.46	1	1
Niger-Congo	Arabian	Southern European	86.50	1	1
Melanesian	Kalash	Arabian	86.69	1	1
Melanesian	Southern European	Omotic	86.81	1	1
Kalash	Northern European	Levantine-Caucasian	86.83	1	1
Khoisan	Cushitic	Berber	86.86	1	1
Kalash	Omotic	Nilo-Saharan	86.87	1	1
Khoisan	Omotic	Niger-Congo	86.91	1	1
Japanese	Pygmy	Arabian	86.98	1	1
Niger-Congo	Native American	Southern European	87.51	1	1
Chinese	Omotic	Arabian	87.55	1	1
Japanese	Arabian	Cushitic	87.88	1	1
Indian	Omotic	Nilo-Saharan	87.96	1	1
Omotic	Siberian	Levantine-Caucasian	87.96	1	1
Kalash	Native American	Southeast Asian	88.11	1	1
Niger-Congo	Siberian	Levantine-Caucasian	88.11	1	1
Arabian	Native American	Cushitic	88.21	1	1
Nilo-Saharan	Kalash	Southern European	88.32	1	1
Berber	Pygmy	Arabian	88.33	1	1
Pygmy	Cushitic	Khoisan	88.38	1	1
Pygmy	Siberian	Levantine-Caucasian	88.56	1	1
Kalash	Arabian	Southern European	88.56	1	1
Japanese	Northern European	Levantine-Caucasian	88.84	1	1
Southern European	Nilo-Saharan	Pygmy	88.87	1	1
Berber	Khoisan	Kalash	89.09	1	1
Pygmy	Indian	Arabian	89.10	1	1
Omotic	Northern European	Nilo-Saharan	89.15	1	1
Khoisan	Chinese	Levantine-Caucasian	89.29	1	1
Southeast Asian	Pygmy	Arabian	89.66	1	1
Melanesian	Khoisan	Native American	89.69	1	1
Nilo-Saharan	Siberian	Northern European	89.79	1	1

Niger-Congo	Omotic	Pygmy	89.85	1	1
Melanesian	Northern European	Native American	89.87	1	1
Japanese	Omotic	Arabian	89.88	1	1
Niger-Congo	Arabian	Northern European	90.15	1	1
Berber	Levantine-Caucasian	Arabian	90.26	1	1
Northern European	Siberian	Berber	90.61	1	1
Melanesian	Northern European	Cushitic	90.73	1	1
Levantine-Caucasian	Niger-Congo	Southern European	90.75	1	1
Pygmy	Kalash	Siberian	90.78	1	1
Levantine-Caucasian	Omotic	Northern European	90.81	1	1
Melanesian	Niger-Congo	Khoisan	90.92	1	1
Melanesian	Arabian	Omotic	90.98	1	1
Southeast Asian	Omotic	Arabian	91.50	1	1
Levantine-Caucasian	Native American	Chinese	91.51	1	1
Nilo-Saharan	Northern European	Pygmy	91.62	1	1
Berber	Northern European	Arabian	91.96	1	1
Khoisan	Indian	Levantine-Caucasian	91.99	1	1
Niger-Congo	Chinese	Arabian	92.31	1	1
Indian	Omotic	Arabian	92.33	1	1
Cushitic	Omotic	Nilo-Saharan	92.49	1	1
Southern European	Southeast Asian	Cushitic	92.56	1	1
Omotic	Native American	Southern European	92.59	1	1
Melanesian	Pygmy	Native American	92.59	1	1
Southern European	Chinese	Cushitic	92.74	1	1
Cushitic	Chinese	Southern European	93.10	1	1
Khoisan	Siberian	Native American	93.14	1	1
Melanesian	Niger-Congo	Native American	93.20	1	1
Omotic	Native American	Pygmy	93.22	1	1
Southern European	Omotic	Nilo-Saharan	93.23	1	1
Melanesian	Siberian	Kalash	93.37	1	1
Kalash	Siberian	Northern European	93.45	1	1
Melanesian	Levantine-Caucasian	Omotic	94.00	1	1
Southeast Asian	Northern European	Arabian	94.14	1	1
Chinese	Northern European	Arabian	94.33	1	1
Levantine-Caucasian	Omotic	Nilo-Saharan	94.45	1	1
Levantine-Caucasian	Siberian	Southern European	94.71	1	1
Chinese	Indian	Kalash	94.79	1	1
Nilo-Saharan	Arabian	Southern European	94.86	1	1
Melanesian	Nilo-Saharan	Southern European	94.89	1	1
Melanesian	Omotic	Niger-Congo	94.99	1	1
Melanesian	Southern European	Native American	95.00	1	1
Nilo-Saharan	Arabian	Pygmy	95.05	1	1
Pygmy	Native American	Southern European	95.27	1	1
Nilo-Saharan	Siberian	Levantine-Caucasian	95.39	1	1
Japanese	Indian	Kalash	95.47	1	1
Melanesian	Omotic	Southern European	95.54	1	1
Niger-Congo	Japanese	Arabian	95.60	1	1
Levantine-Caucasian	Chinese	Southern European	95.61	1	1
Japanese	Cushitic	Southern European	95.63	1	1
Khoisan	Japanese	Levantine-Caucasian	95.66	1	1
Southeast Asian	Levantine-Caucasian	Southern European	95.69	1	1

Kalash	Northern European	Indian	95.89	1	1
Native American	Northern European	Berber	96.11	1	1
Levantine-Caucasian	Nilo-Saharan	Pygmy	96.44	1	1
Melanesian	Khoisan	Southern European	96.53	1	1
Khoisan	Chinese	Northern European	96.68	1	1
Arabian	Siberian	Cushitic	96.79	1	1
Southeast Asian	Khoisan	Levantine-Caucasian	97.04	1	1
Southeast Asian	Indian	Kalash	97.06	1	1
Pygmy	Cushitic	Berber	97.19	1	1
Pygmy	Siberian	Native American	97.23	1	1
Berber	Omotic	Arabian	97.26	1	1
Indian	Arabian	Northern European	97.51	1	1
Kalash	Native American	Chinese	97.66	1	1
Melanesian	Southeast Asian	Indian	97.68	1	1
Indian	Northern European	Levantine-Caucasian	97.71	1	1
Pygmy	Omotic	Khoisan	97.75	1	1
Melanesian	Chinese	Indian	97.91	1	1
Nilo-Saharan	Chinese	Arabian	97.93	1	1
Southern European	Japanese	Cushitic	97.96	1	1
Southeast Asian	Cushitic	Southern European	98.00	1	1
Berber	Pygmy	Indian	98.09	1	1
Nilo-Saharan	Indian	Pygmy	98.09	1	1
Berber	Omotic	Nilo-Saharan	98.18	1	1
Melanesian	Nilo-Saharan	Native American	98.52	1	1
Southeast Asian	Niger-Congo	Arabian	98.56	1	1
Native American	Siberian	Southeast Asian	98.92	1	1
Levantine-Caucasian	Japanese	Southern European	98.98	1	1
Levantine-Caucasian	Omotic	Southern European	99.02	1	1
Khoisan	Japanese	Northern European	99.11	1	1
Berber	Pygmy	Kalash	99.20	1	1
Melanesian	Japanese	Indian	99.32	1	1
Khoisan	Northern European	Siberian	99.46	1	1
Khoisan	Kalash	Japanese	99.58	1	1
Nilo-Saharan	Arabian	Northern European	99.73	1	1
Pygmy	Indian	Levantine-Caucasian	99.97	1	1
Nilo-Saharan	Native American	Southern European	99.99	1	1
Levantine-Caucasian	Nilo-Saharan	Southern European	100.02	1	1
Southern European	Native American	Cushitic	100.02	1	1
Japanese	Nilo-Saharan	Arabian	100.09	1	1
Omotic	Arabian	Nilo-Saharan	100.18	1	1
Melanesian	Pygmy	Southern European	100.55	1	1
Melanesian	Levantine-Caucasian	Native American	101.05	1	1
Indian	Omotic	Levantine-Caucasian	101.07	1	1
Chinese	Kalash	Northern European	101.15	1	1
Japanese	Northern European	Arabian	101.22	1	1
Omotic	Arabian	Southern European	101.38	1	1
Southeast Asian	Kalash	Northern European	101.76	1	1
Southeast Asian	Nilo-Saharan	Arabian	102.19	1	1
Cushitic	Northern European	Indian	102.22	1	1
Southeast Asian	Khoisan	Northern European	102.34	1	1
Levantine-Caucasian	Kalash	Indian	102.35	1	1

Southeast Asian	Japanese	Siberian	102.65	1	1
Melanesian	Omotic	Native American	102.76	1	1
Berber	Khoisan	Levantine-Caucasian	103.06	1	1
Berber	Cushitic	Arabian	103.39	1	1
Melanesian	Cushitic	Native American	103.47	1	1
Kalash	Native American	Levantine-Caucasian	103.49	1	1
Pygmy	Chinese	Levantine-Caucasian	103.64	1	1
Melanesian	Kalash	Native American	103.69	1	1
Cushitic	Omotic	Berber	103.81	1	1
Japanese	Kalash	Northern European	103.88	1	1
Melanesian	Siberian	Native American	104.01	1	1
Melanesian	Arabian	Native American	104.01	1	1
Northern European	Siberian	Levantine-Caucasian	104.25	1	1
Nilo-Saharan	Indian	Arabian	104.29	1	1
Omotic	Siberian	Pygmy	104.30	1	1
Niger-Congo	Indian	Arabian	104.33	1	1
Japanese	Pygmy	Levantine-Caucasian	104.43	1	1
Pygmy	Kalash	Japanese	104.52	1	1
Niger-Congo	Kalash	Siberian	104.75	1	1
Khoisan	Indian	Siberian	104.81	1	1
Southern European	Levantine-Caucasian	Arabian	104.98	1	1
Niger-Congo	Pygmy	Omotic	105.04	1	1
Levantine-Caucasian	Kalash	Northern European	105.09	1	1
Khoisan	Indian	Northern European	105.25	1	1
Japanese	Omotic	Levantine-Caucasian	105.64	1	1
Khoisan	Kalash	Southeast Asian	105.67	1	1
Southeast Asian	Levantine-Caucasian	Cushitic	105.98	1	1
Kalash	Native American	Berber	106.00	1	1
Levantine-Caucasian	Indian	Northern European	106.22	1	1
Southeast Asian	Omotic	Pygmy	106.30	1	1
Southeast Asian	Pygmy	Levantine-Caucasian	106.34	1	1
Chinese	Omotic	Levantine-Caucasian	106.48	1	1
Levantine-Caucasian	Indian	Berber	106.64	1	1
Southeast Asian	Kalash	Berber	106.65	1	1
Nilo-Saharan	Omotic	Niger-Congo	106.74	1	1
Melanesian	Northern European	Omotic	106.89	1	1
Berber	Levantine-Caucasian	Southern European	106.97	1	1
Chinese	Omotic	Pygmy	106.99	1	1
Cushitic	Indian	Northern European	107.06	1	1
Japanese	Pygmy	Northern European	107.08	1	1
Indian	Arabian	Southern European	107.11	1	1
Japanese	Omotic	Pygmy	107.27	1	1
Nilo-Saharan	Kalash	Siberian	107.31	1	1
Melanesian	Berber	Native American	107.32	1	1
Khoisan	Japanese	Native American	107.35	1	1
Levantine-Caucasian	Chinese	Cushitic	107.38	1	1
Southeast Asian	Omotic	Levantine-Caucasian	107.44	1	1
Pygmy	Northern European	Siberian	107.45	1	1
Chinese	Kalash	Berber	107.52	1	1
Berber	Indian	Cushitic	107.60	1	1
Berber	Niger-Congo	Pygmy	107.69	1	1

Southern European	Berber	Arabian	108.11	1	1
Chinese	Siberian	Southeast Asian	108.19	1	1
Japanese	Omotic	Northern European	108.68	1	1
Omotic	Native American	Niger-Congo	108.70	1	1
Southern European	Khoisan	Siberian	108.93	1	1
Southern European	Northern European	Berber	109.27	1	1
Berber	Nilo-Saharan	Arabian	109.28	1	1
Melanesian	Nilo-Saharan	Khoisan	109.30	1	1
Southern European	Siberian	Cushitic	109.32	1	1
Pygmy	Kalash	Southeast Asian	109.34	1	1
Native American	Northern European	Southeast Asian	109.46	1	1
Niger-Congo	Cushitic	Berber	109.60	1	1
Southeast Asian	Pygmy	Northern European	109.60	1	1
Pygmy	Chinese	Northern European	109.62	1	1
Native American	Northern European	Levantine-Caucasian	109.99	1	1
Cushitic	Indian	Omotic	110.01	1	1
Berber	Nilo-Saharan	Pygmy	110.17	1	1
Southeast Asian	Kalash	Levantine-Caucasian	110.30	1	1
Japanese	Kalash	Berber	110.33	1	1
Cushitic	Indian	Southern European	110.64	1	1
Kalash	Siberian	Levantine-Caucasian	110.70	1	1
Japanese	Pygmy	Native American	110.72	1	1
Cushitic	Kalash	Omotic	110.75	1	1
Melanesian	Indian	Berber	110.97	1	1
Levantine-Caucasian	Japanese	Cushitic	111.23	1	1
Chinese	Omotic	Northern European	111.37	1	1
Omotic	Arabian	Northern European	111.49	1	1
Melanesian	Southeast Asian	Kalash	111.60	1	1
Chinese	Kalash	Levantine-Caucasian	111.67	1	1
Kalash	Siberian	Berber	111.95	1	1
Berber	Native American	Omotic	111.98	1	1
Khoisan	Siberian	Southern European	112.02	1	1
Berber	Pygmy	Levantine-Caucasian	112.39	1	1
Arabian	Northern European	Kalash	112.52	1	1
Niger-Congo	Cushitic	Pygmy	112.66	1	1
Indian	Northern European	Berber	112.92	1	1
Japanese	Kalash	Levantine-Caucasian	112.99	1	1
Melanesian	Japanese	Kalash	112.99	1	1
Pygmy	Indian	Siberian	113.15	1	1
Melanesian	Cushitic	Nilo-Saharan	113.19	1	1
Berber	Arabian	Southern European	113.23	1	1
Berber	Kalash	Cushitic	113.28	1	1
Southeast Asian	Omotic	Northern European	113.37	1	1
Niger-Congo	Indian	Levantine-Caucasian	113.65	1	1
Levantine-Caucasian	Indian	Arabian	113.66	1	1
Omotic	Siberian	Native American	113.71	1	1
Melanesian	Chinese	Kalash	113.71	1	1
Nilo-Saharan	Siberian	Native American	113.90	1	1
Northern European	Siberian	Arabian	113.91	1	1
Levantine-Caucasian	Cushitic	Southern European	113.91	1	1
Niger-Congo	Siberian	Native American	113.94	1	1

Melanesian	Kalash	Southern European	114.00	1	1
Kalash	Native American	Arabian	114.44	1	1
Southeast Asian	Berber	Omotic	114.49	1	1
Pygmy	Indian	Northern European	114.56	1	1
Southeast Asian	Niger-Congo	Levantine-Caucasian	114.71	1	1
Niger-Congo	Japanese	Levantine-Caucasian	114.92	1	1
Niger-Congo	Japanese	Northern European	114.95	1	1
Niger-Congo	Chinese	Levantine-Caucasian	114.98	1	1
Nilo-Saharan	Indian	Levantine-Caucasian	115.02	1	1
Southern European	Pygmy	Siberian	115.19	1	1
Southeast Asian	Northern European	Southern European	115.21	1	1
Khoisan	Native American	Melanesian	115.37	1	1
Kalash	Omotic	Pygmy	115.51	1	1
Levantine-Caucasian	Cushitic	Kalash	115.63	1	1
Khoisan	Chinese	Native American	115.68	1	1
Levantine-Caucasian	Native American	Cushitic	115.96	1	1
Nilo-Saharan	Omotic	Pygmy	116.14	1	1
Southern European	Levantine-Caucasian	Northern European	116.31	1	1
Indian	Native American	Northern European	116.74	1	1
Kalash	Omotic	Siberian	116.99	1	1
Southeast Asian	Native American	Indian	117.61	1	1
Berber	Chinese	Omotic	117.62	1	1
Omotic	Siberian	Southern European	117.69	1	1
Levantine-Caucasian	Northern European	Berber	118.21	1	1
Melanesian	Kalash	Cushitic	118.27	1	1
Khoisan	Indian	Japanese	118.36	1	1
Levantine-Caucasian	Kalash	Berber	118.36	1	1
Southeast Asian	Niger-Congo	Northern European	118.66	1	1
Pygmy	Native American	Melanesian	118.76	1	1
Southeast Asian	Kalash	Arabian	118.79	1	1
Berber	Niger-Congo	Arabian	118.79	1	1
Chinese	Northern European	Southern European	118.83	1	1
Pygmy	Siberian	Southern European	118.84	1	1
Levantine-Caucasian	Kalash	Arabian	118.87	1	1
Kalash	Northern European	Berber	118.94	1	1
Omotic	Siberian	Niger-Congo	119.00	1	1
Niger-Congo	Chinese	Northern European	119.09	1	1
Pygmy	Chinese	Native American	119.22	1	1
Melanesian	Native American	Berber	119.32	1	1
Chinese	Kalash	Arabian	119.40	1	1
Japanese	Nilo-Saharan	Levantine-Caucasian	119.43	1	1
Kalash	Siberian	Arabian	119.70	1	1
Native American	Northern European	Arabian	119.71	1	1
Berber	Khoisan	Southern European	119.77	1	1
Southeast Asian	Khoisan	Native American	119.93	1	1
Nilo-Saharan	Cushitic	Berber	119.94	1	1
Niger-Congo	Kalash	Japanese	119.97	1	1
Chinese	Omotic	Niger-Congo	120.10	1	1
Southeast Asian	Omotic	Niger-Congo	120.22	1	1
Berber	Japanese	Omotic	120.32	1	1
Levantine-Caucasian	Siberian	Cushitic	120.34	1	1

Southeast Asian	Nilo-Saharan	Levantine-Caucasian	120.50	1	1
Indian	Omotic	Northern European	121.03	1	1
Southern European	Cushitic	Northern European	121.62	1	1
Melanesian	Kalash	Omotic	121.98	1	1
Japanese	Northern European	Southern European	122.00	1	1
Japanese	Kalash	Arabian	122.13	1	1
Japanese	Omotic	Niger-Congo	122.14	1	1
Levantine-Caucasian	Khoisan	Siberian	122.17	1	1
Japanese	Siberian	Southeast Asian	122.21	1	1
Nilo-Saharan	Chinese	Levantine-Caucasian	122.29	1	1
Nilo-Saharan	Cushitic	Pygmy	122.44	1	1
Southeast Asian	Pygmy	Native American	122.46	1	1
Kalash	Siberian	Native American	122.54	1	1
Japanese	Northern European	Native American	122.65	1	1
Berber	Omotic	Levantine-Caucasian	122.71	1	1
Japanese	Nilo-Saharan	Northern European	122.80	1	1
Nilo-Saharan	Kalash	Japanese	123.15	1	1
Cushitic	Siberian	Native American	123.69	1	1
Berber	Khoisan	Northern European	123.94	1	1
Khoisan	Indian	Southeast Asian	124.09	1	1
Melanesian	Omotic	Khoisan	124.41	1	1
Berber	Omotic	Kalash	124.44	1	1
Berber	Siberian	Omotic	124.54	1	1
Northern European	Siberian	Native American	124.81	1	1
Indian	Omotic	Pygmy	124.94	1	1
Southern European	Cushitic	Kalash	125.03	1	1
Native American	Northern European	Chinese	125.32	1	1
Khoisan	Northern European	Japanese	125.48	1	1
Berber	Siberian	Native American	125.74	1	1
Melanesian	Indian	Native American	125.76	1	1
Melanesian	Cushitic	Pygmy	125.83	1	1
Japanese	Kalash	Native American	126.10	1	1
Khoisan	Pygmy	Omotic	126.21	1	1
Pygmy	Indian	Japanese	126.38	1	1
Southeast Asian	Nilo-Saharan	Northern European	126.42	1	1
Niger-Congo	Siberian	Southern European	126.65	1	1
Southern European	Siberian	Native American	126.67	1	1
Berber	Northern European	Kalash	126.87	1	1
Southeast Asian	Native American	Kalash	126.90	1	1
Melanesian	Indian	Arabian	127.10	1	1
Khoisan	Arabian	Siberian	127.30	1	1
Omotic	Northern European	Pygmy	127.67	1	1
Nilo-Saharan	Chinese	Northern European	127.71	1	1
Niger-Congo	Kalash	Southeast Asian	127.84	1	1
Berber	Arabian	Levantine-Caucasian	127.86	1	1
Arabian	Siberian	Native American	128.08	1	1
Kalash	Northern European	Arabian	128.26	1	1
Nilo-Saharan	Kalash	Southeast Asian	128.36	1	1
Berber	Nilo-Saharan	Indian	128.74	1	1
Cushitic	Arabian	Levantine-Caucasian	128.75	1	1
Levantine-Caucasian	Arabian	Southern European	128.81	1	1

Levantine-Caucasian	Pygmy	Siberian	128.89	1	1
Berber	Omotic	Indian	129.09	1	1
Levantine-Caucasian	Siberian	Native American	129.17	1	1
Berber	Nilo-Saharan	Kalash	129.36	1	1
Levantine-Caucasian	Northern European	Kalash	129.67	1	1
Melanesian	Japanese	Native American	129.70	1	1
Khoisan	Siberian	Melanesian	129.86	1	1
Pygmy	Northern European	Japanese	129.93	1	1
Japanese	Omotic	Native American	130.08	1	1
Pygmy	Nilo-Saharan	Khoisan	130.15	1	1
Pygmy	Siberian	Melanesian	130.15	1	1
Northern European	Siberian	Southern European	130.29	1	1
Indian	Northern European	Arabian	130.37	1	1
Southeast Asian	Chinese	Siberian	130.37	1	1
Pygmy	Indian	Southeast Asian	130.49	1	1
Pygmy	Arabian	Siberian	130.60	1	1
Berber	Pygmy	Southern European	130.61	1	1
Arabian	Native American	Omotic	130.82	1	1
Kalash	Northern European	Southern European	130.95	1	1
Kalash	Omotic	Japanese	131.04	1	1
Southern European	Japanese	Native American	131.19	1	1
Berber	Pygmy	Northern European	131.50	1	1
Melanesian	Indian	Northern European	131.73	1	1
Southern European	Omotic	Pygmy	131.78	1	1
Kalash	Omotic	Niger-Congo	132.12	1	1
Chinese	Kalash	Native American	132.42	1	1
Nilo-Saharan	Siberian	Southern European	133.72	1	1
Chinese	Northern European	Native American	133.74	1	1
Japanese	Arabian	Native American	134.08	1	1
Southeast Asian	Arabian	Omotic	134.12	1	1
Levantine-Caucasian	Omotic	Pygmy	134.19	1	1
Southern European	Khoisan	Japanese	134.44	1	1
Berber	Japanese	Native American	134.54	1	1
Levantine-Caucasian	Arabian	Kalash	134.58	1	1
Indian	Native American	Berber	134.76	1	1
Niger-Congo	Indian	Northern European	134.99	1	1
Japanese	Nilo-Saharan	Native American	135.02	1	1
Omotic	Arabian	Pygmy	135.18	1	1
Southern European	Pygmy	Japanese	135.21	1	1
Berber	Levantine-Caucasian	Northern European	135.31	1	1
Niger-Congo	Nilo-Saharan	Omotic	135.35	1	1
Khoisan	Northern European	Southeast Asian	135.40	1	1
Southeast Asian	Pygmy	Melanesian	135.41	1	1
Nilo-Saharan	Indian	Northern European	135.67	1	1
Southeast Asian	Kalash	Native American	135.69	1	1
Niger-Congo	Japanese	Native American	135.71	1	1
Southern European	Arabian	Levantine-Caucasian	135.92	1	1
Berber	Niger-Congo	Kalash	135.98	1	1
Berber	Niger-Congo	Indian	136.04	1	1
Japanese	Cushitic	Native American	136.07	1	1
Melanesian	Southeast Asian	Native American	136.19	1	1

Southeast Asian	Khoisan	Melanesian	136.21	1	1
Cushitic	Northern European	Omotic	136.52	1	1
Khoisan	Japanese	Southern European	136.72	1	1
Melanesian	Chinese	Native American	136.96	1	1
Indian	Arabian	Cushitic	136.97	1	1
Cushitic	Omotic	Pygmy	136.98	1	1
Pygmy	Northern European	Southeast Asian	137.13	1	1
Japanese	Pygmy	Melanesian	137.21	1	1
Southeast Asian	Northern European	Native American	137.25	1	1
Kalash	Omotic	Southeast Asian	137.29	1	1
Indian	Kalash	Levantine-Caucasian	137.47	1	1
Berber	Omotic	Pygmy	137.55	1	1
Levantine-Caucasian	Japanese	Native American	137.57	1	1
Southeast Asian	Khoisan	Southern European	137.58	1	1
Berber	Levantine-Caucasian	Kalash	137.61	1	1
Indian	Omotic	Niger-Congo	137.70	1	1
Melanesian	Native American	Northern European	137.75	1	1
Levantine-Caucasian	Cushitic	Northern European	137.89	1	1
Chinese	Arabian	Omotic	137.99	1	1
Japanese	Chinese	Siberian	138.09	1	1
Khoisan	Chinese	Southern European	138.22	1	1
Berber	Arabian	Cushitic	138.35	1	1
Melanesian	Indian	Levantine-Caucasian	138.63	1	1
Berber	Nilo-Saharan	Levantine-Caucasian	138.76	1	1
Khoisan	Japanese	Melanesian	138.86	1	1
Khoisan	Indian	Southern European	138.88	1	1
Berber	Omotic	Southern European	138.88	1	1
Japanese	Arabian	Omotic	139.58	1	1
Nilo-Saharan	Northern European	Siberian	139.69	1	1
Indian	Omotic	Siberian	139.95	1	1
Chinese	Omotic	Native American	140.09	1	1
Pygmy	Kalash	Chinese	140.10	1	1
Berber	Omotic	Niger-Congo	140.28	1	1
Japanese	Omotic	Southern European	140.49	1	1
Southern European	Levantine-Caucasian	Kalash	140.85	1	1
Southeast Asian	Omotic	Native American	140.96	1	1
Omotic	Native American	Melanesian	141.03	1	1
Southern European	Pygmy	Southeast Asian	141.20	1	1
Southeast Asian	Omotic	Southern European	141.31	1	1
Nilo-Saharan	Indian	Siberian	141.44	1	1
Levantine-Caucasian	Cushitic	Indian	141.72	1	1
Southern European	Khoisan	Southeast Asian	141.72	1	1
Omotic	Northern European	Niger-Congo	141.77	1	1
Melanesian	Native American	Omotic	141.91	1	1
Cushitic	Omotic	Niger-Congo	141.95	1	1
Southern European	Southeast Asian	Omotic	141.98	1	1
Cushitic	Arabian	Southern European	142.05	1	1
Melanesian	Native American	Arabian	142.12	1	1
Southern European	Native American	Omotic	142.13	1	1
Japanese	Pygmy	Southern European	142.14	1	1
Khoisan	Kalash	Chinese	142.59	1	1

Indian	Northern European	Southern European	142.67	1	1
Niger-Congo	Indian	Siberian	142.70	1	1
Southeast Asian	Pygmy	Southern European	142.88	1	1
Southeast Asian	Northern European	Cushitic	142.92	1	1
Southern European	Chinese	Native American	143.28	1	1
Kalash	Native American	Southern European	143.37	1	1
Niger-Congo	Native American	Khoisan	143.74	1	1
Levantine-Caucasian	Northern European	Southern European	143.81	1	1
Pygmy	Indian	Southern European	143.83	1	1
Niger-Congo	Northern European	Siberian	143.90	1	1
Niger-Congo	Pygmy	Khoisan	144.00	1	1
Kalash	Arabian	Cushitic	144.09	1	1
Native American	Northern European	Southern European	144.22	1	1
Berber	Chinese	Native American	144.40	1	1
Southern European	Berber	Levantine-Caucasian	144.85	1	1
Chinese	Arabian	Native American	144.94	1	1
Melanesian	Indian	Omotic	145.21	1	1
Indian	Omotic	Southern European	145.39	1	1
Arabian	Siberian	Omotic	145.47	1	1
Levantine-Caucasian	Khoisan	Japanese	146.06	1	1
Southern European	Northern European	Levantine-Caucasian	146.08	1	1
Chinese	Omotic	Southern European	146.15	1	1
Levantine-Caucasian	Northern European	Arabian	146.22	1	1
Cushitic	Arabian	Kalash	146.52	1	1
Indian	Kalash	Northern European	146.75	1	1
Southern European	Southeast Asian	Native American	146.76	1	1
Southeast Asian	Berber	Native American	146.78	1	1
Southern European	Omotic	Niger-Congo	146.93	1	1
Southeast Asian	Levantine-Caucasian	Omotic	147.51	1	1
Southeast Asian	Nilo-Saharan	Native American	147.51	1	1
Niger-Congo	Nilo-Saharan	Pygmy	147.55	1	1
Cushitic	Native American	Nilo-Saharan	147.60	1	1
Native American	Siberian	Kalash	147.60	1	1
Pygmy	Chinese	Southern European	147.69	1	1
Southeast Asian	Arabian	Native American	147.89	1	1
Indian	Siberian	Native American	147.90	1	1
Nilo-Saharan	Chinese	Native American	147.98	1	1
Southern European	Cushitic	Indian	148.05	1	1
Melanesian	Indian	Cushitic	148.18	1	1
Cushitic	Chinese	Native American	148.49	1	1
Omotic	Northern European	Siberian	148.90	1	1
Levantine-Caucasian	Native American	Omotic	148.93	1	1
Melanesian	Siberian	Berber	148.98	1	1
Berber	Niger-Congo	Levantine-Caucasian	149.05	1	1
Levantine-Caucasian	Kalash	Southern European	149.21	1	1
Southern European	Japanese	Omotic	149.27	1	1
Kalash	Siberian	Southern European	149.47	1	1
Japanese	Chinese	Southeast Asian	149.60	1	1
Berber	Omotic	Northern European	149.63	1	1
Southern European	Northern European	Arabian	149.72	1	1
Nilo-Saharan	Native American	Khoisan	149.72	1	1

Levantine-Caucasian	Chinese	Native American	149.94	1	1
Nilo-Saharan	Native American	Melanesian	150.09	1	1
Southern European	Chinese	Omotic	150.10	1	1
Levantine-Caucasian	Omotic	Niger-Congo	150.28	1	1
Levantine-Caucasian	Pygmy	Japanese	150.44	1	1
Berber	Cushitic	Levantine-Caucasian	150.46	1	1
Southeast Asian	Niger-Congo	Native American	150.61	1	1
Levantine-Caucasian	Arabian	Northern European	150.62	1	1
Southeast Asian	Levantine-Caucasian	Native American	150.69	1	1
Khoisan	Arabian	Japanese	150.71	1	1
Pygmy	Kalash	Native American	150.86	1	1
Pygmy	Chinese	Melanesian	151.10	1	1
Khoisan	Kalash	Native American	151.15	1	1
Berber	Levantine-Caucasian	Cushitic	151.32	1	1
Southeast Asian	Cushitic	Native American	151.56	1	1
Southeast Asian	Kalash	Southern European	151.99	1	1
Niger-Congo	Chinese	Native American	152.12	1	1
Pygmy	Arabian	Japanese	152.16	1	1
Levantine-Caucasian	Khoisan	Southeast Asian	152.17	1	1
Omotic	Arabian	Niger-Congo	152.54	1	1
Southern European	Nilo-Saharan	Siberian	152.56	1	1
Omotic	Siberian	Melanesian	152.57	1	1
Japanese	Northern European	Cushitic	152.64	1	1
Indian	Omotic	Japanese	152.65	1	1
Berber	Cushitic	Southern European	152.99	1	1
Chinese	Native American	Indian	153.01	1	1
Levantine-Caucasian	Pygmy	Southeast Asian	153.17	1	1
Chinese	Northern European	Cushitic	153.20	1	1
Cushitic	Kalash	Siberian	153.58	1	1
Levantine-Caucasian	Japanese	Omotic	153.59	1	1
Chinese	Native American	Kalash	153.60	1	1
Indian	Native American	Arabian	153.69	1	1
Southern European	Siberian	Omotic	153.70	1	1
Southeast Asian	Kalash	Cushitic	153.79	1	1
Nilo-Saharan	Indian	Japanese	154.12	1	1
Indian	Native American	Levantine-Caucasian	154.21	1	1
Levantine-Caucasian	Cushitic	Omotic	154.65	1	1
Pygmy	Arabian	Southeast Asian	154.76	1	1
Japanese	Native American	Kalash	154.89	1	1
Indian	Kalash	Berber	154.96	1	1
Native American	Siberian	Indian	154.97	1	1
Indian	Siberian	Berber	154.98	1	1
Levantine-Caucasian	Indian	Southern European	155.05	1	1
Japanese	Native American	Indian	155.10	1	1
Levantine-Caucasian	Chinese	Omotic	155.68	1	1
Japanese	Kalash	Southern European	155.84	1	1
Berber	Cushitic	Omotic	155.88	1	1
Arabian	Northern European	Indian	156.05	1	1
Khoisan	Chinese	Melanesian	156.63	1	1
Southern European	Indian	Cushitic	156.86	1	1
Melanesian	Cushitic	Niger-Congo	157.13	1	1

Niger-Congo	Indian	Japanese	157.16	1	1
Berber	Arabian	Kalash	157.17	1	1
Khoisan	Arabian	Southeast Asian	157.50	1	1
Chinese	Kalash	Southern European	157.61	1	1
Melanesian	Native American	Cushitic	157.80	1	1
Levantine-Caucasian	Siberian	Omotic	158.08	1	1
Indian	Omotic	Southeast Asian	158.11	1	1
Southern European	Arabian	Kalash	158.14	1	1
Southern European	Northern European	Kalash	158.23	1	1
Southern European	Cushitic	Omotic	158.46	1	1
Kalash	Native American	Cushitic	158.47	1	1
Southern European	Berber	Northern European	158.51	1	1
Nilo-Saharan	Indian	Southeast Asian	158.72	1	1
Southern European	Arabian	Northern European	158.74	1	1
Pygmy	Kalash	Melanesian	158.92	1	1
Omotic	Native American	Khoisan	159.06	1	1
Southern European	Niger-Congo	Siberian	159.22	1	1
Southeast Asian	Cushitic	Nilo-Saharan	159.39	1	1
Berber	Khoisan	Siberian	159.40	1	1
Khoisan	Cushitic	Kalash	159.41	1	1
Niger-Congo	Siberian	Khoisan	159.68	1	1
Pygmy	Omotic	Cushitic	159.77	1	1
Berber	Northern European	Cushitic	159.79	1	1
Southeast Asian	Omotic	Melanesian	159.84	1	1
Cushitic	Chinese	Nilo-Saharan	159.92	1	1
Indian	Kalash	Arabian	160.27	1	1
Khoisan	Pygmy	Nilo-Saharan	160.63	1	1
Japanese	Cushitic	Nilo-Saharan	160.63	1	1
Berber	Indian	Omotic	160.87	1	1
Chinese	Kalash	Cushitic	161.17	1	1
Khoisan	Omotic	Cushitic	161.36	1	1
Melanesian	Native American	Levantine-Caucasian	161.36	1	1
Berber	Nilo-Saharan	Southern European	161.41	1	1
Japanese	Kalash	Cushitic	161.52	1	1
Khoisan	Cushitic	Arabian	161.62	1	1
Niger-Congo	Native American	Melanesian	161.64	1	1
Indian	Siberian	Northern European	161.66	1	1
Nilo-Saharan	Siberian	Melanesian	162.05	1	1
Khoisan	Kalash	Melanesian	162.19	1	1
Cushitic	Siberian	Nilo-Saharan	162.63	1	1
Japanese	Omotic	Melanesian	162.65	1	1
Niger-Congo	Omotic	Cushitic	163.17	1	1
Kalash	Siberian	Cushitic	163.66	1	1
Melanesian	Berber	Nilo-Saharan	164.08	1	1
Melanesian	Siberian	Omotic	164.19	1	1
Niger-Congo	Indian	Southeast Asian	164.22	1	1
Pygmy	Cushitic	Kalash	164.98	1	1
Cushitic	Arabian	Omotic	165.05	1	1
Southern European	Omotic	Siberian	165.32	1	1
Niger-Congo	Japanese	Southern European	165.35	1	1
Cushitic	Native American	Melanesian	165.54	1	1

Melanesian	Berber	Pygmy	165.58	1	1
Southeast Asian	Kalash	Omotic	165.62	1	1
Berber	Nilo-Saharan	Northern European	165.63	1	1
Khoisan	Cushitic	Indian	165.71	1	1
Nilo-Saharan	Arabian	Siberian	165.90	1	1
Niger-Congo	Kalash	Chinese	166.20	1	1
Southeast Asian	Nilo-Saharan	Southern European	166.40	1	1
Berber	Pygmy	Siberian	166.41	1	1
Nilo-Saharan	Kalash	Chinese	166.48	1	1
Japanese	Nilo-Saharan	Southern European	166.53	1	1
Berber	Northern European	Indian	166.73	1	1
Nilo-Saharan	Siberian	Khoisan	166.75	1	1
Southeast Asian	Niger-Congo	Southern European	166.96	1	1
Berber	Kalash	Omotic	167.01	1	1
Levantine-Caucasian	Nilo-Saharan	Siberian	167.28	1	1
Southeast Asian	Northern European	Omotic	167.31	1	1
Nilo-Saharan	Northern European	Japanese	167.44	1	1
Southeast Asian	Niger-Congo	Khoisan	167.63	1	1
Southern European	Kalash	Cushitic	167.78	1	1
Niger-Congo	Kalash	Khoisan	167.78	1	1
Kalash	Omotic	Chinese	167.80	1	1
Northern European	Siberian	Cushitic	167.85	1	1
Kalash	Native American	Omotic	167.97	1	1
Cushitic	Native American	Pygmy	167.97	1	1
Berber	Arabian	Northern European	168.27	1	1
Berber	Cushitic	Kalash	168.66	1	1
Melanesian	Southeast Asian	Berber	168.87	1	1
Niger-Congo	Arabian	Siberian	168.88	1	1
Cushitic	Omotic	Arabian	169.05	1	1
Japanese	Indian	Native American	169.75	1	1
Cushitic	Indian	Siberian	170.50	1	1
Melanesian	Indian	Southern European	170.58	1	1
Pygmy	Cushitic	Arabian	170.79	1	1
Pygmy	Indian	Chinese	170.93	1	1
Melanesian	Japanese	Berber	171.05	1	1
Khoisan	Pygmy	Niger-Congo	171.24	1	1
Khoisan	Indian	Chinese	171.29	1	1
Nilo-Saharan	Indian	Southern European	171.35	1	1
Niger-Congo	Siberian	Melanesian	171.49	1	1
Southeast Asian	Indian	Berber	171.63	1	1
Niger-Congo	Indian	Southern European	171.64	1	1
Southern European	Berber	Kalash	171.71	1	1
Japanese	Indian	Berber	171.98	1	1
Berber	Niger-Congo	Southern European	172.14	1	1
Pygmy	Cushitic	Indian	172.27	1	1
Native American	Northern European	Cushitic	172.30	1	1
Japanese	Kalash	Omotic	172.41	1	1
Levantine-Caucasian	Niger-Congo	Siberian	172.52	1	1
Omotic	Siberian	Khoisan	172.55	1	1
Niger-Congo	Japanese	Khoisan	172.74	1	1
Nilo-Saharan	Omotic	Cushitic	173.24	1	1

Melanesian	Siberian	Arabian	173.52	1	1
Levantine-Caucasian	Omotic	Siberian	173.74	1	1
Berber	Kalash	Siberian	173.84	1	1
Chinese	Indian	Berber	173.86	1	1
Niger-Congo	Northern European	Japanese	174.02	1	1
Melanesian	Chinese	Berber	174.05	1	1
Nilo-Saharan	Kalash	Khoisan	174.14	1	1
Kalash	Siberian	Omotic	174.25	1	1
Cushitic	Arabian	Indian	174.67	1	1
Chinese	Kalash	Omotic	174.68	1	1
Southeast Asian	Nilo-Saharan	Melanesian	174.78	1	1
Indian	Siberian	Arabian	175.02	1	1
Southeast Asian	Nilo-Saharan	Khoisan	175.05	1	1
Berber	Niger-Congo	Northern European	175.45	1	1
Southeast Asian	Kalash	Melanesian	175.52	1	1
Omotic	Northern European	Japanese	175.74	1	1
Berber	Native American	Melanesian	175.85	1	1
Niger-Congo	Chinese	Khoisan	176.00	1	1
Omotic	Arabian	Siberian	176.14	1	1
Arabian	Northern European	Cushitic	176.16	1	1
Kalash	Native American	Melanesian	176.48	1	1
Niger-Congo	Chinese	Southern European	176.79	1	1
Nilo-Saharan	Northern European	Southeast Asian	176.92	1	1
Southern European	Berber	Cushitic	177.03	1	1
Cushitic	Siberian	Melanesian	177.16	1	1
Levantine-Caucasian	Arabian	Cushitic	177.50	1	1
Melanesian	Southeast Asian	Omotic	177.58	1	1
Chinese	Omotic	Melanesian	177.71	1	1
Southeast Asian	Omotic	Khoisan	177.89	1	1
Kalash	Omotic	Native American	177.90	1	1
Nilo-Saharan	Chinese	Southern European	177.98	1	1
Japanese	Nilo-Saharan	Khoisan	178.05	1	1
Japanese	Nilo-Saharan	Melanesian	178.05	1	1
Berber	Indian	Siberian	178.51	1	1
Japanese	Northern European	Omotic	178.78	1	1
Southern European	Nilo-Saharan	Japanese	179.01	1	1
Cushitic	Omotic	Kalash	179.13	1	1
Southeast Asian	Siberian	Indian	179.64	1	1
Cushitic	Kalash	Japanese	179.88	1	1
Nilo-Saharan	Kalash	Native American	179.90	1	1
Kalash	Siberian	Melanesian	179.99	1	1
Southeast Asian	Siberian	Native American	180.57	1	1
Melanesian	Japanese	Omotic	180.70	1	1
Melanesian	Siberian	Cushitic	180.84	1	1
Melanesian	Siberian	Northern European	180.89	1	1
Japanese	Omotic	Khoisan	180.98	1	1
Japanese	Kalash	Melanesian	181.14	1	1
Niger-Congo	Kalash	Native American	181.44	1	1
Indian	Kalash	Siberian	181.85	1	1
Nilo-Saharan	Chinese	Khoisan	181.98	1	1
Omotic	Northern European	Southeast Asian	182.02	1	1

Chinese	Northern European	Omotic	182.52	1	1
Indian	Siberian	Levantine-Caucasian	182.59	1	1
Pygmy	Northern European	Chinese	182.70	1	1
Berber	Levantine-Caucasian	Indian	182.71	1	1
Chinese	Indian	Native American	182.79	1	1
Levantine-Caucasian	Indian	Cushitic	182.99	1	1
Melanesian	Arabian	Pygmy	183.17	1	1
Levantine-Caucasian	Arabian	Indian	183.69	1	1
Southeast Asian	Cushitic	Melanesian	183.79	1	1
Cushitic	Siberian	Pygmy	183.88	1	1
Berber	Siberian	Melanesian	184.13	1	1
Pygmy	Northern European	Native American	184.24	1	1
Southern European	Nilo-Saharan	Southeast Asian	184.29	1	1
Chinese	Omotic	Khoisan	184.31	1	1
Melanesian	Chinese	Omotic	184.58	1	1
Kalash	Omotic	Khoisan	185.16	1	1
Southeast Asian	Berber	Melanesian	185.16	1	1
Berber	Khoisan	Japanese	185.32	1	1
Niger-Congo	Northern European	Southeast Asian	185.42	1	1
Arabian	Native American	Melanesian	185.44	1	1
Levantine-Caucasian	Northern European	Indian	185.49	1	1
Southeast Asian	Indian	Native American	185.69	1	1
Southeast Asian	Cushitic	Pygmy	185.96	1	1
Cushitic	Kalash	Southeast Asian	186.11	1	1
Japanese	Cushitic	Melanesian	186.13	1	1
Southeast Asian	Siberian	Kalash	186.29	1	1
Melanesian	Southern European	Pygmy	186.37	1	1
Indian	Native American	Omotic	186.48	1	1
Cushitic	Arabian	Northern European	186.67	1	1
Southeast Asian	Niger-Congo	Melanesian	186.73	1	1
Native American	Northern European	Omotic	186.77	1	1
Southern European	Niger-Congo	Japanese	186.90	1	1
Japanese	Cushitic	Pygmy	187.19	1	1
Indian	Native American	Cushitic	187.26	1	1
Berber	Pygmy	Japanese	187.33	1	1
Kalash	Omotic	Melanesian	187.85	1	1
Northern European	Siberian	Omotic	188.04	1	1
Khoisan	Northern European	Native American	188.19	1	1
Southern European	Arabian	Cushitic	188.44	1	1
Pygmy	Indian	Native American	188.57	1	1
Khoisan	Indian	Native American	189.76	1	1
Melanesian	Arabian	Nilo-Saharan	189.82	1	1
Melanesian	Southern European	Nilo-Saharan	189.94	1	1
Kalash	Arabian	Siberian	190.13	1	1
Southern European	Pygmy	Chinese	190.24	1	1
Khoisan	Northern European	Chinese	190.53	1	1
Berber	Japanese	Melanesian	190.67	1	1
Southeast Asian	Native American	Melanesian	190.73	1	1
Niger-Congo	Omotic	Khoisan	190.79	1	1
Cushitic	Omotic	Indian	190.91	1	1
Berber	Khoisan	Southeast Asian	191.06	1	1

Melanesian	Levantine-Caucasian	Pygmy	191.06	1	1
Cushitic	Chinese	Pygmy	191.22	1	1
Melanesian	Native American	Southern European	191.33	1	1
Southern European	Omotic	Japanese	191.41	1	1
Niger-Congo	Japanese	Melanesian	191.45	1	1
Pygmy	Indian	Melanesian	191.82	1	1
Cushitic	Indian	Japanese	192.02	1	1
Arabian	Siberian	Melanesian	192.50	1	1
Indian	Arabian	Siberian	192.56	1	1
Berber	Pygmy	Southeast Asian	192.64	1	1
Melanesian	Cushitic	Khoisan	192.66	1	1
Levantine-Caucasian	Nilo-Saharan	Japanese	193.14	1	1
Nilo-Saharan	Arabian	Japanese	193.25	1	1
Chinese	Kalash	Melanesian	193.39	1	1
Southeast Asian	Arabian	Melanesian	193.49	1	1
Levantine-Caucasian	Kalash	Cushitic	194.42	1	1
Southern European	Omotic	Southeast Asian	194.48	1	1
Niger-Congo	Arabian	Khoisan	194.60	1	1
Southern European	Niger-Congo	Southeast Asian	194.72	1	1
Melanesian	Siberian	Levantine-Caucasian	194.76	1	1
Khoisan	Nilo-Saharan	Cushitic	195.16	1	1
Indian	Arabian	Omotic	195.53	1	1
Indian	Northern European	Siberian	195.60	1	1
Melanesian	Levantine-Caucasian	Nilo-Saharan	195.61	1	1
Southern European	Niger-Congo	Khoisan	195.84	1	1
Southern European	Nilo-Saharan	Khoisan	195.93	1	1
Berber	Cushitic	Northern European	196.39	1	1
Indian	Kalash	Southern European	196.45	1	1
Cushitic	Indian	Southeast Asian	196.46	1	1
Berber	Cushitic	Indian	196.68	1	1
Melanesian	Kalash	Pygmy	196.92	1	1
Niger-Congo	Arabian	Japanese	196.95	1	1
Nilo-Saharan	Arabian	Khoisan	197.86	1	1
Southern European	Indian	Siberian	198.02	1	1
Southern European	Pygmy	Native American	198.43	1	1
Kalash	Arabian	Omotic	198.43	1	1
Japanese	Indian	Northern European	198.50	1	1
Indian	Native American	Southern European	198.55	1	1
Melanesian	Northern European	Pygmy	198.58	1	1
Nilo-Saharan	Arabian	Southeast Asian	198.60	1	1
Nilo-Saharan	Kalash	Melanesian	198.66	1	1
Japanese	Indian	Arabian	198.76	1	1
Berber	Arabian	Indian	198.76	1	1
Levantine-Caucasian	Omotic	Japanese	198.77	1	1
Japanese	Arabian	Melanesian	198.91	1	1
Levantine-Caucasian	Nilo-Saharan	Southeast Asian	199.02	1	1
Indian	Native American	Melanesian	199.03	1	1
Khoisan	Indian	Melanesian	199.10	1	1
Southern European	Levantine-Caucasian	Indian	199.53	1	1
Indian	Omotic	Chinese	199.59	1	1
Southeast Asian	Indian	Arabian	199.80	1	1

Southern European	Native American	Melanesian	199.91	1	1
Melanesian	Southeast Asian	Arabian	200.08	1	1
Melanesian	Southeast Asian	Cushitic	200.11	1	1
Southern European	Kalash	Siberian	201.07	1	1
Indian	Siberian	Omotic	201.28	1	1
Levantine-Caucasian	Niger-Congo	Japanese	201.32	1	1
Indian	Siberian	Cushitic	201.48	1	1
Kalash	Northern European	Siberian	201.59	1	1
Chinese	Indian	Arabian	201.68	1	1
Berber	Omotic	Siberian	201.70	1	1
Indian	Northern European	Cushitic	201.96	1	1
Omotic	Arabian	Japanese	201.97	1	1
Nilo-Saharan	Indian	Khoisan	202.00	1	1
Southeast Asian	Native American	Berber	202.13	1	1
Southern European	Siberian	Melanesian	202.32	1	1
Nilo-Saharan	Northern European	Khoisan	202.54	1	1
Niger-Congo	Indian	Khoisan	202.75	1	1
Southeast Asian	Indian	Northern European	202.83	1	1
Niger-Congo	Arabian	Southeast Asian	203.06	1	1
Melanesian	Japanese	Arabian	203.06	1	1
Southern European	Khoisan	Chinese	203.23	1	1
Pygmy	Arabian	Chinese	203.53	1	1
Levantine-Caucasian	Omotic	Southeast Asian	203.54	1	1
Cushitic	Native American	Niger-Congo	203.69	1	1
Levantine-Caucasian	Niger-Congo	Khoisan	203.95	1	1
Chinese	Indian	Northern European	204.01	1	1
Levantine-Caucasian	Pygmy	Chinese	204.03	1	1
Nilo-Saharan	Chinese	Melanesian	204.05	1	1
Cushitic	Kalash	Nilo-Saharan	204.06	1	1
Melanesian	Japanese	Cushitic	204.15	1	1
Niger-Congo	Northern European	Khoisan	204.30	1	1
Levantine-Caucasian	Native American	Melanesian	204.31	1	1
Omotic	Arabian	Southeast Asian	204.33	1	1
Kalash	Northern European	Cushitic	204.43	1	1
Indian	Omotic	Khoisan	204.44	1	1
Berber	Indian	Japanese	204.88	1	1
Levantine-Caucasian	Nilo-Saharan	Khoisan	205.24	1	1
Levantine-Caucasian	Pygmy	Native American	205.29	1	1
Cushitic	Indian	Nilo-Saharan	205.92	1	1
Berber	Kalash	Japanese	205.98	1	1
Pygmy	Arabian	Native American	206.02	1	1
Levantine-Caucasian	Siberian	Melanesian	206.32	1	1
Southern European	Southeast Asian	Melanesian	206.62	1	1
Nilo-Saharan	Cushitic	Kalash	207.71	1	1
Cushitic	Chinese	Melanesian	207.82	1	1
Native American	Siberian	Melanesian	208.00	1	1
Nilo-Saharan	Cushitic	Arabian	208.02	1	1
Indian	Siberian	Melanesian	208.07	1	1
Southern European	Khoisan	Native American	208.39	1	1
Levantine-Caucasian	Niger-Congo	Southeast Asian	208.39	1	1
Melanesian	Chinese	Arabian	208.40	1	1

Southeast Asian	Levantine-Caucasian	Melanesian	208.98	1	1
Southeast Asian	Indian	Omotic	209.20	1	1
Khoisan	Cushitic	Levantine-Caucasian	209.24	1	1
Cushitic	Omotic	Levantine-Caucasian	209.35	1	1
Nilo-Saharan	Omotic	Khoisan	209.58	1	1
Southern European	Indian	Omotic	209.67	1	1
Pygmy	Arabian	Melanesian	209.73	1	1
Omotic	Northern European	Khoisan	209.75	1	1
Indian	Kalash	Cushitic	209.80	1	1
Berber	Chinese	Melanesian	209.83	1	1
Japanese	Indian	Levantine-Caucasian	209.85	1	1
Niger-Congo	Kalash	Melanesian	209.96	1	1
Pygmy	Cushitic	Levantine-Caucasian	209.99	1	1
Melanesian	Chinese	Cushitic	210.25	1	1
Melanesian	Northern European	Nilo-Saharan	210.51	1	1
Pygmy	Omotic	Berber	210.85	1	1
Berber	Nilo-Saharan	Siberian	210.87	1	1
Berber	Indian	Southeast Asian	211.03	1	1
Nilo-Saharan	Indian	Chinese	211.37	1	1
Southern European	Arabian	Indian	211.43	1	1
Southern European	Omotic	Khoisan	211.95	1	1
Southern European	Japanese	Melanesian	211.96	1	1
Japanese	Indian	Omotic	211.99	1	1
Pygmy	Nilo-Saharan	Cushitic	212.09	1	1
Southeast Asian	Indian	Levantine-Caucasian	212.19	1	1
Levantine-Caucasian	Khoisan	Native American	212.37	1	1
Levantine-Caucasian	Indian	Siberian	212.46	1	1
Niger-Congo	Cushitic	Kalash	213.12	1	1
Berber	Kalash	Southeast Asian	213.13	1	1
Cushitic	Northern European	Siberian	213.14	1	1
Omotic	Arabian	Khoisan	213.29	1	1
Indian	Kalash	Japanese	213.35	1	1
Southern European	Levantine-Caucasian	Cushitic	213.56	1	1
Melanesian	Native American	Pygmy	213.77	1	1
Melanesian	Berber	Niger-Congo	214.16	1	1
Niger-Congo	Cushitic	Arabian	214.19	1	1
Pygmy	Northern European	Melanesian	214.34	1	1
Southeast Asian	Indian	Cushitic	214.36	1	1
Melanesian	Kalash	Nilo-Saharan	214.42	1	1
Khoisan	Omotic	Berber	214.63	1	1
Southern European	Pygmy	Melanesian	214.86	1	1
Levantine-Caucasian	Japanese	Melanesian	214.91	1	1
Khoisan	Arabian	Native American	215.30	1	1
Levantine-Caucasian	Khoisan	Chinese	215.64	1	1
Japanese	Native American	Melanesian	215.71	1	1
Japanese	Siberian	Native American	216.00	1	1
Cushitic	Kalash	Pygmy	216.06	1	1
Khoisan	Arabian	Chinese	216.10	1	1
Chinese	Indian	Levantine-Caucasian	216.26	1	1
Southeast Asian	Northern European	Melanesian	216.28	1	1
Southern European	Northern European	Indian	216.34	1	1

Berber	Native American	Nilo-Saharan	216.63	1	1
Indian	Omotic	Native American	216.65	1	1
Southern European	Kalash	Omotic	217.02	1	1
Levantine-Caucasian	Kalash	Siberian	217.23	1	1
Berber	Omotic	Khoisan	217.35	1	1
Chinese	Indian	Omotic	217.38	1	1
Japanese	Indian	Cushitic	217.49	1	1
Nilo-Saharan	Cushitic	Indian	217.88	1	1
Indian	Kalash	Southeast Asian	217.89	1	1
Levantine-Caucasian	Omotic	Khoisan	218.02	1	1
Cushitic	Omotic	Khoisan	218.05	1	1
Southeast Asian	Indian	Melanesian	218.14	1	1
Levantine-Caucasian	Pygmy	Melanesian	218.96	1	1
Niger-Congo	Indian	Chinese	219.21	1	1
Cushitic	Siberian	Niger-Congo	219.34	1	1
Berber	Native American	Pygmy	219.39	1	1
Southeast Asian	Cushitic	Niger-Congo	219.45	1	1
Berber	Nilo-Saharan	Khoisan	219.52	1	1
Cushitic	Kalash	Native American	220.04	1	1
Northern European	Siberian	Melanesian	220.24	1	1
Southern European	Berber	Indian	220.52	1	1
Melanesian	Southeast Asian	Northern European	220.78	1	1
Niger-Congo	Omotic	Berber	220.93	1	1
Southeast Asian	Berber	Nilo-Saharan	221.00	1	1
Indian	Omotic	Melanesian	221.13	1	1
Chinese	Siberian	Native American	221.16	1	1
Melanesian	Japanese	Northern European	221.46	1	1
Japanese	Cushitic	Niger-Congo	221.58	1	1
Japanese	Indian	Melanesian	221.86	1	1
Melanesian	Indian	Pygmy	222.28	1	1
Berber	Niger-Congo	Siberian	222.56	1	1
Niger-Congo	Cushitic	Indian	222.65	1	1
Indian	Siberian	Southern European	222.70	1	1
Indian	Kalash	Omotic	222.74	1	1
Khoisan	Arabian	Melanesian	223.11	1	1
Omotic	Northern European	Native American	223.31	1	1
Berber	Arabian	Omotic	223.55	1	1
Khoisan	Cushitic	Siberian	223.55	1	1
Chinese	Indian	Cushitic	223.62	1	1
Southeast Asian	Native American	Omotic	223.95	1	1
Chinese	Arabian	Melanesian	224.30	1	1
Melanesian	Siberian	Southern European	224.32	1	1
Native American	Siberian	Berber	224.41	1	1
Southeast Asian	Siberian	Melanesian	224.59	1	1
Levantine-Caucasian	Indian	Omotic	224.63	1	1
Pygmy	Cushitic	Siberian	224.69	1	1
Berber	Northern European	Omotic	224.69	1	1
Cushitic	Chinese	Niger-Congo	225.04	1	1
Indian	Arabian	Japanese	225.11	1	1
Japanese	Northern European	Melanesian	225.53	1	1
Berber	Pygmy	Melanesian	226.68	1	1

Berber	Omotic	Japanese	226.72	1	1
Berber	Niger-Congo	Khoisan	226.98	1	1
Native American	Northern European	Melanesian	227.08	1	1
Berber	Levantine-Caucasian	Omotic	227.19	1	1
Berber	Siberian	Nilo-Saharan	227.48	1	1
Cushitic	Indian	Pygmy	227.54	1	1
Kalash	Arabian	Japanese	227.82	1	1
Melanesian	Southeast Asian	Levantine-Caucasian	227.83	1	1
Berber	Japanese	Nilo-Saharan	228.01	1	1
Niger-Congo	Chinese	Melanesian	228.06	1	1
Chinese	Native American	Melanesian	228.92	1	1
Khoisan	Niger-Congo	Cushitic	229.79	1	1
Indian	Arabian	Southeast Asian	230.03	1	1
Cushitic	Kalash	Chinese	230.06	1	1
Southeast Asian	Native American	Northern European	230.16	1	1
Nilo-Saharan	Omotic	Berber	230.41	1	1
Berber	Omotic	Southeast Asian	230.45	1	1
Berber	Siberian	Pygmy	230.47	1	1
Melanesian	Japanese	Levantine-Caucasian	230.48	1	1
Southeast Asian	Berber	Pygmy	230.64	1	1
Japanese	Siberian	Kalash	230.76	1	1
Omotic	Northern European	Chinese	231.62	1	1
Levantine-Caucasian	Kalash	Omotic	231.80	1	1
Cushitic	Kalash	Melanesian	231.99	1	1
Nilo-Saharan	Indian	Native American	232.59	1	1
Chinese	Siberian	Kalash	232.66	1	1
Khoisan	Northern European	Melanesian	233.00	1	1
Nilo-Saharan	Cushitic	Khoisan	233.20	1	1
Indian	Northern European	Omotic	233.24	1	1
Berber	Pygmy	Native American	233.44	1	1
Kalash	Northern European	Omotic	233.53	1	1
Melanesian	Chinese	Northern European	233.91	1	1
Nilo-Saharan	Northern European	Native American	234.35	1	1
Southern European	Indian	Japanese	234.64	1	1
Melanesian	Siberian	Pygmy	234.70	1	1
Japanese	Native American	Berber	234.72	1	1
Berber	Chinese	Nilo-Saharan	234.94	1	1
Niger-Congo	Cushitic	Khoisan	234.95	1	1
Kalash	Arabian	Southeast Asian	235.37	1	1
Nilo-Saharan	Northern European	Chinese	235.85	1	1
Chinese	Native American	Berber	236.64	1	1
Berber	Kalash	Native American	236.84	1	1
Indian	Northern European	Japanese	236.88	1	1
Berber	Japanese	Pygmy	236.89	1	1
Berber	Khoisan	Melanesian	237.21	1	1
Berber	Pygmy	Chinese	237.83	1	1
Southeast Asian	Native American	Arabian	238.01	1	1
Levantine-Caucasian	Khoisan	Melanesian	238.03	1	1
Cushitic	Omotic	Southern European	238.10	1	1
Pygmy	Cushitic	Northern European	238.15	1	1
Berber	Khoisan	Native American	238.42	1	1

Melanesian	Berber	Khoisan	238.51	1	1
Southern European	Khoisan	Melanesian	238.52	1	1
Khoisan	Cushitic	Northern European	239.05	1	1
Berber	Nilo-Saharan	Japanese	239.57	1	1
Khoisan	Cushitic	Southern European	239.71	1	1
Pygmy	Cushitic	Southern European	239.85	1	1
Japanese	Siberian	Indian	239.97	1	1
Levantine-Caucasian	Northern European	Cushitic	240.70	1	1
Southern European	Indian	Southeast Asian	240.95	1	1
Pygmy	Omotic	Kalash	241.73	1	1
Melanesian	Chinese	Levantine-Caucasian	242.03	1	1
Niger-Congo	Indian	Native American	242.27	1	1
Native American	Siberian	Omotic	242.76	1	1
Chinese	Indian	Melanesian	243.07	1	1
Melanesian	Native American	Nilo-Saharan	243.19	1	1
Berber	Nilo-Saharan	Southeast Asian	243.46	1	1
Chinese	Siberian	Indian	243.88	1	1
Southern European	Kalash	Japanese	244.19	1	1
Native American	Siberian	Northern European	244.49	1	1
Melanesian	Indian	Nilo-Saharan	244.51	1	1
Indian	Northern European	Southeast Asian	244.76	1	1
Cushitic	Omotic	Northern European	244.98	1	1
Berber	Chinese	Pygmy	245.34	1	1
Khoisan	Omotic	Kalash	245.49	1	1
Southern European	Omotic	Native American	245.97	1	1
Southeast Asian	Native American	Cushitic	246.02	1	1
Berber	Khoisan	Chinese	246.50	1	1
Southeast Asian	Japanese	Kalash	246.66	1	1
Southern European	Chinese	Melanesian	246.85	1	1
Nilo-Saharan	Indian	Melanesian	246.88	1	1
Southeast Asian	Japanese	Indian	247.27	1	1
Kalash	Northern European	Japanese	248.19	1	1
Levantine-Caucasian	Chinese	Melanesian	248.27	1	1
Indian	Kalash	Native American	248.41	1	1
Southeast Asian	Japanese	Native American	248.43	1	1
Cushitic	Omotic	Siberian	248.45	1	1
Arabian	Native American	Pygmy	248.47	1	1
Southern European	Berber	Omotic	248.58	1	1
Niger-Congo	Pygmy	Cushitic	248.65	1	1
Berber	Kalash	Melanesian	248.67	1	1
Southern European	Nilo-Saharan	Chinese	249.34	1	1
Southern European	Omotic	Chinese	249.44	1	1
Omotic	Arabian	Melanesian	249.89	1	1
Levantine-Caucasian	Indian	Japanese	249.92	1	1
Southern European	Kalash	Southeast Asian	249.99	1	1
Levantine-Caucasian	Omotic	Native American	250.26	1	1
Japanese	Native American	Omotic	250.37	1	1
Niger-Congo	Northern European	Native American	250.41	1	1
Arabian	Northern European	Omotic	250.49	1	1
Omotic	Northern European	Melanesian	250.58	1	1
Cushitic	Northern European	Nilo-Saharan	250.80	1	1

Omotic	Arabian	Native American	251.24	1	1
Melanesian	Southeast Asian	Pygmy	251.70	1	1
Pygmy	Cushitic	Southeast Asian	251.91	1	1
Niger-Congo	Northern European	Chinese	252.51	1	1
Pygmy	Cushitic	Japanese	252.87	1	1
Cushitic	Indian	Chinese	252.98	1	1
Cushitic	Indian	Native American	254.33	1	1
Melanesian	Arabian	Niger-Congo	254.37	1	1
Chinese	Native American	Omotic	254.93	1	1
Southern European	Cushitic	Siberian	255.02	1	1
Levantine-Caucasian	Indian	Southeast Asian	255.18	1	1
Southern European	Northern European	Cushitic	255.27	1	1
Levantine-Caucasian	Cushitic	Siberian	255.51	1	1
Japanese	Indian	Southern European	255.66	1	1
Berber	Niger-Congo	Japanese	255.70	1	1
Khoisan	Cushitic	Japanese	255.81	1	1
Kalash	Arabian	Native American	256.01	1	1
Southeast Asian	Indian	Southern European	256.06	1	1
Southern European	Omotic	Melanesian	256.15	1	1
Melanesian	Japanese	Pygmy	256.23	1	1
Omotic	Arabian	Chinese	256.52	1	1
Khoisan	Cushitic	Southeast Asian	256.72	1	1
Native American	Siberian	Arabian	256.81	1	1
Southern European	Nilo-Saharan	Native American	256.83	1	1
Kalash	Northern European	Southeast Asian	257.67	1	1
Levantine-Caucasian	Omotic	Chinese	257.95	1	1
Arabian	Native American	Nilo-Saharan	258.05	1	1
Melanesian	Southern European	Niger-Congo	258.15	1	1
Berber	Cushitic	Nilo-Saharan	258.19	1	1
Berber	Indian	Native American	258.20	1	1
Cushitic	Northern European	Japanese	258.29	1	1
Levantine-Caucasian	Omotic	Melanesian	258.56	1	1
Nilo-Saharan	Cushitic	Levantine-Caucasian	258.81	1	1
Nilo-Saharan	Arabian	Native American	258.89	1	1
Berber	Northern European	Siberian	259.06	1	1
Southeast Asian	Siberian	Berber	259.44	1	1
Melanesian	Arabian	Khoisan	259.45	1	1
Cushitic	Native American	Khoisan	259.57	1	1
Kalash	Native American	Pygmy	259.64	1	1
Berber	Kalash	Chinese	259.65	1	1
Chinese	Northern European	Melanesian	259.89	1	1
Berber	Omotic	Melanesian	260.10	1	1
Melanesian	Southeast Asian	Southern European	260.15	1	1
Levantine-Caucasian	Arabian	Omotic	260.36	1	1
Nilo-Saharan	Arabian	Chinese	260.45	1	1
Levantine-Caucasian	Kalash	Japanese	260.50	1	1
Arabian	Siberian	Pygmy	260.55	1	1
Berber	Niger-Congo	Southeast Asian	260.60	1	1
Japanese	Siberian	Melanesian	260.66	1	1
Southeast Asian	Arabian	Pygmy	260.90	1	1
Pygmy	Omotic	Indian	261.34	1	1

Khoisan	Nilo-Saharan	Berber	262.22	1	1
Indian	Kalash	Melanesian	262.26	1	1
Cushitic	Northern European	Pygmy	262.58	1	1
Southeast Asian	Native American	Levantine-Caucasian	263.18	1	1
Melanesian	Japanese	Southern European	263.34	1	1
Southeast Asian	Arabian	Nilo-Saharan	263.44	1	1
Kalash	Northern European	Native American	263.53	1	1
Cushitic	Northern European	Southeast Asian	263.69	1	1
Melanesian	Levantine-Caucasian	Niger-Congo	264.27	1	1
Arabian	Northern European	Siberian	264.35	1	1
Levantine-Caucasian	Nilo-Saharan	Native American	264.99	1	1
Cushitic	Arabian	Siberian	265.12	1	1
Southeast Asian	Kalash	Pygmy	265.48	1	1
Kalash	Siberian	Pygmy	265.55	1	1
Melanesian	Chinese	Pygmy	265.59	1	1
Levantine-Caucasian	Nilo-Saharan	Chinese	265.88	1	1
Cushitic	Kalash	Niger-Congo	266.01	1	1
Cushitic	Indian	Melanesian	266.50	1	1
Native American	Siberian	Cushitic	266.61	1	1
Melanesian	Siberian	Nilo-Saharan	266.91	1	1
Southern European	Native American	Pygmy	266.99	1	1
Chinese	Indian	Southern European	267.04	1	1
Levantine-Caucasian	Kalash	Southeast Asian	267.27	1	1
Southeast Asian	Siberian	Omotic	267.47	1	1
Berber	Indian	Chinese	267.53	1	1
Southern European	Niger-Congo	Chinese	267.82	1	1
Khoisan	Omotic	Indian	267.87	1	1
Melanesian	Southern European	Khoisan	267.97	1	1
Niger-Congo	Cushitic	Levantine-Caucasian	268.18	1	1
Japanese	Arabian	Pygmy	268.68	1	1
Japanese	Native American	Northern European	268.79	1	1
Berber	Cushitic	Pygmy	268.82	1	1
Pygmy	Cushitic	Melanesian	268.87	1	1
Kalash	Arabian	Melanesian	268.87	1	1
Berber	Omotic	Native American	269.21	1	1
Arabian	Siberian	Nilo-Saharan	269.61	1	1
Southeast Asian	Japanese	Melanesian	269.90	1	1
Southern European	Kalash	Native American	270.26	1	1
Niger-Congo	Indian	Melanesian	270.46	1	1
Cushitic	Indian	Niger-Congo	271.11	1	1
Levantine-Caucasian	Native American	Pygmy	271.16	1	1
Indian	Kalash	Chinese	271.20	1	1
Japanese	Arabian	Nilo-Saharan	271.26	1	1
Melanesian	Kalash	Khoisan	271.29	1	1
Berber	Cushitic	Siberian	271.61	1	1
Niger-Congo	Arabian	Native American	271.68	1	1
Southeast Asian	Chinese	Kalash	272.00	1	1
Chinese	Siberian	Melanesian	272.30	1	1
Japanese	Kalash	Pygmy	272.41	1	1
Niger-Congo	Omotic	Kalash	272.66	1	1
Berber	Kalash	Pygmy	272.84	1	1

Nilo-Saharan	Omotic	Kalash	272.85	1	1
Niger-Congo	Arabian	Chinese	273.06	1	1
Levantine-Caucasian	Cushitic	Nilo-Saharan	274.39	1	1
Khoisan	Pygmy	Cushitic	274.55	1	1
Southern European	Arabian	Omotic	274.71	1	1
Japanese	Native American	Arabian	274.75	1	1
Berber	Indian	Melanesian	274.95	1	1
Pygmy	Nilo-Saharan	Berber	275.23	1	1
Melanesian	Levantine-Caucasian	Khoisan	275.49	1	1
Cushitic	Siberian	Khoisan	275.70	1	1
Nilo-Saharan	Arabian	Melanesian	275.82	1	1
Southern European	Siberian	Pygmy	276.25	1	1
Chinese	Native American	Northern European	276.91	1	1
Melanesian	Kalash	Niger-Congo	277.23	1	1
Cushitic	Omotic	Southeast Asian	277.32	1	1
Niger-Congo	Nilo-Saharan	Cushitic	277.60	1	1
Southern European	Niger-Congo	Native American	278.16	1	1
Indian	Arabian	Native American	278.33	1	1
Levantine-Caucasian	Siberian	Pygmy	278.40	1	1
Melanesian	Chinese	Southern European	278.59	1	1
Indian	Northern European	Native American	278.91	1	1
Native American	Siberian	Levantine-Caucasian	279.09	1	1
Southern European	Southeast Asian	Pygmy	279.26	1	1
Southeast Asian	Chinese	Indian	279.69	1	1
Japanese	Native American	Cushitic	279.83	1	1
Chinese	Native American	Arabian	279.89	1	1
Southern European	Native American	Nilo-Saharan	280.15	1	1
Berber	Omotic	Chinese	280.46	1	1
Cushitic	Omotic	Japanese	280.54	1	1
Berber	Indian	Nilo-Saharan	280.83	1	1
Nilo-Saharan	Cushitic	Siberian	280.87	1	1
Levantine-Caucasian	Cushitic	Pygmy	280.95	1	1
Southeast Asian	Levantine-Caucasian	Pygmy	280.97	1	1
Berber	Indian	Pygmy	281.25	1	1
Chinese	Arabian	Pygmy	281.53	1	1
Berber	Native American	Niger-Congo	281.62	1	1
Levantine-Caucasian	Niger-Congo	Native American	281.65	1	1
Melanesian	Northern European	Niger-Congo	281.65	1	1
Berber	Kalash	Nilo-Saharan	281.95	1	1
Chinese	Kalash	Pygmy	282.06	1	1
Levantine-Caucasian	Kalash	Native American	282.20	1	1
Berber	Arabian	Siberian	282.52	1	1
Kalash	Native American	Nilo-Saharan	282.77	1	1
Berber	Levantine-Caucasian	Siberian	282.80	1	1
Southeast Asian	Kalash	Nilo-Saharan	282.88	1	1
Levantine-Caucasian	Niger-Congo	Chinese	283.04	1	1
Southern European	Southeast Asian	Nilo-Saharan	283.27	1	1
Chinese	Arabian	Nilo-Saharan	283.42	1	1
Southeast Asian	Chinese	Native American	283.43	1	1
Southern European	Cushitic	Nilo-Saharan	283.51	1	1
Khoisan	Cushitic	Melanesian	283.79	1	1

Southeast Asian	Cushitic	Khoisan	284.24	1	1
Melanesian	Northern European	Khoisan	284.31	1	1
Pygmy	Omotic	Siberian	284.82	1	1
Chinese	Native American	Cushitic	285.32	1	1
Levantine-Caucasian	Native American	Nilo-Saharan	285.75	1	1
Pygmy	Omotic	Arabian	286.33	1	1
Cushitic	Arabian	Pygmy	286.56	1	1
Southern European	Cushitic	Pygmy	286.69	1	1
Kalash	Siberian	Nilo-Saharan	287.02	1	1
Southeast Asian	Levantine-Caucasian	Nilo-Saharan	287.13	1	1
Southern European	Japanese	Pygmy	287.24	1	1
Levantine-Caucasian	Japanese	Pygmy	287.78	1	1
Southern European	Kalash	Melanesian	287.85	1	1
Indian	Native American	Pygmy	288.08	1	1
Southeast Asian	Berber	Niger-Congo	288.10	1	1
Cushitic	Arabian	Nilo-Saharan	288.73	1	1
Japanese	Cushitic	Khoisan	288.86	1	1
Southern European	Levantine-Caucasian	Omotic	288.89	1	1
Melanesian	Southeast Asian	Nilo-Saharan	289.64	1	1
Southern European	Indian	Native American	290.26	1	1
Nilo-Saharan	Northern European	Melanesian	290.41	1	1
Levantine-Caucasian	Siberian	Nilo-Saharan	290.54	1	1
Southern European	Siberian	Nilo-Saharan	290.67	1	1
Kalash	Arabian	Chinese	290.78	1	1
Berber	Siberian	Niger-Congo	291.52	1	1
Japanese	Kalash	Nilo-Saharan	291.53	1	1
Southern European	Japanese	Nilo-Saharan	291.91	1	1
Niger-Congo	Omotic	Indian	292.36	1	1
Berber	Nilo-Saharan	Native American	292.65	1	1
Berber	Nilo-Saharan	Melanesian	292.66	1	1
Pygmy	Cushitic	Native American	292.98	1	1
Niger-Congo	Cushitic	Siberian	292.99	1	1
Levantine-Caucasian	Northern European	Omotic	293.30	1	1
Khoisan	Omotic	Arabian	294.03	1	1
Southern European	Nilo-Saharan	Melanesian	294.15	1	1
Melanesian	Japanese	Nilo-Saharan	294.29	1	1
Nilo-Saharan	Cushitic	Southern European	294.38	1	1
Kalash	Northern European	Melanesian	294.81	1	1
Khoisan	Omotic	Siberian	295.00	1	1
Levantine-Caucasian	Japanese	Nilo-Saharan	295.28	1	1
Southeast Asian	Siberian	Cushitic	295.82	1	1
Nilo-Saharan	Omotic	Indian	295.92	1	1
Cushitic	Northern European	Native American	296.03	1	1
Nilo-Saharan	Cushitic	Northern European	296.74	1	1
Indian	Arabian	Chinese	296.87	1	1
Southeast Asian	Northern European	Pygmy	297.08	1	1
Levantine-Caucasian	Nilo-Saharan	Melanesian	297.15	1	1
Khoisan	Niger-Congo	Berber	297.26	1	1
Berber	Japanese	Niger-Congo	297.32	1	1
Khoisan	Nilo-Saharan	Kalash	297.80	1	1
Levantine-Caucasian	Kalash	Melanesian	297.86	1	1

Indian	Arabian	Melanesian	298.46	1	1
Niger-Congo	Nilo-Saharan	Khoisan	298.92	1	1
Cushitic	Chinese	Khoisan	299.56	1	1
Levantine-Caucasian	Arabian	Siberian	299.98	1	1
Southeast Asian	Siberian	Arabian	300.01	1	1
Levantine-Caucasian	Cushitic	Japanese	300.47	1	1
Levantine-Caucasian	Indian	Native American	301.02	1	1
Northern European	Siberian	Pygmy	301.32	1	1
Southeast Asian	Chinese	Melanesian	301.50	1	1
Japanese	Native American	Levantine-Caucasian	301.67	1	1
Chinese	Kalash	Nilo-Saharan	301.69	1	1
Indian	Siberian	Pygmy	302.06	1	1
Levantine-Caucasian	Cushitic	Southeast Asian	302.53	1	1
Native American	Northern European	Pygmy	302.95	1	1
Cushitic	Omotic	Melanesian	303.17	1	1
Khoisan	Cushitic	Native American	303.19	1	1
Southeast Asian	Native American	Southern European	303.48	1	1
Southern European	Cushitic	Japanese	303.63	1	1
Pygmy	Nilo-Saharan	Kalash	303.85	1	1
Niger-Congo	Cushitic	Southern European	303.94	1	1
Southern European	Berber	Siberian	305.11	1	1
Niger-Congo	Arabian	Melanesian	305.44	1	1
Levantine-Caucasian	Northern European	Siberian	305.79	1	1
Southern European	Chinese	Pygmy	305.92	1	1
Melanesian	Chinese	Nilo-Saharan	305.93	1	1
Southern European	Cushitic	Southeast Asian	306.02	1	1
Levantine-Caucasian	Chinese	Pygmy	306.51	1	1
Niger-Congo	Cushitic	Northern European	306.64	1	1
Berber	Nilo-Saharan	Chinese	306.78	1	1
Japanese	Northern European	Pygmy	307.83	1	1
Japanese	Chinese	Native American	307.85	1	1
Melanesian	Indian	Khoisan	307.92	1	1
Pygmy	Omotic	Melanesian	308.19	1	1
Southern European	Northern European	Omotic	308.65	1	1
Japanese	Chinese	Kalash	308.73	1	1
Japanese	Siberian	Omotic	308.73	1	1
Berber	Chinese	Niger-Congo	308.93	1	1
Southern European	Chinese	Nilo-Saharan	309.18	1	1
Japanese	Siberian	Berber	310.14	1	1
Melanesian	Native American	Khoisan	310.18	1	1
Cushitic	Kalash	Khoisan	310.47	1	1
Levantine-Caucasian	Chinese	Nilo-Saharan	310.69	1	1
Southeast Asian	Northern European	Nilo-Saharan	310.82	1	1
Niger-Congo	Pygmy	Berber	310.89	1	1
Kalash	Arabian	Pygmy	311.08	1	1
Cushitic	Arabian	Japanese	311.23	1	1
Indian	Native American	Nilo-Saharan	312.08	1	1
Cushitic	Arabian	Southeast Asian	312.20	1	1
Berber	Cushitic	Japanese	312.70	1	1
Southern European	Arabian	Siberian	313.42	1	1
Chinese	Native American	Levantine-Caucasian	313.69	1	1

Pygmy	Cushitic	Chinese	313.73	1	1
Berber	Niger-Congo	Native American	313.80	1	1
Southern European	Kalash	Chinese	313.84	1	1
Southeast Asian	Siberian	Northern European	314.76	1	1
Southern European	Indian	Chinese	314.92	1	1
Melanesian	Indian	Niger-Congo	315.17	1	1
Berber	Northern European	Japanese	315.29	1	1
Chinese	Siberian	Omotic	315.30	1	1
Nilo-Saharan	Cushitic	Southeast Asian	315.34	1	1
Berber	Cushitic	Southeast Asian	315.65	1	1
Native American	Siberian	Southern European	316.14	1	1
Southeast Asian	Native American	Pygmy	316.18	1	1
Pygmy	Omotic	Southeast Asian	316.38	1	1
Chinese	Siberian	Berber	316.61	1	1
Cushitic	Omotic	Native American	316.66	1	1
Southeast Asian	Japanese	Omotic	317.78	1	1
Indian	Northern European	Chinese	317.81	1	1
Southeast Asian	Indian	Pygmy	318.23	1	1
Melanesian	Siberian	Khoisan	318.29	1	1
Nilo-Saharan	Cushitic	Japanese	318.46	1	1
Pygmy	Omotic	Japanese	318.70	1	1
Berber	Native American	Khoisan	318.95	1	1
Kalash	Northern European	Chinese	319.93	1	1
Nilo-Saharan	Omotic	Arabian	321.56	1	1
Southern European	Indian	Melanesian	321.65	1	1
Berber	Northern European	Southeast Asian	322.33	1	1
Berber	Niger-Congo	Melanesian	322.43	1	1
Japanese	Indian	Pygmy	322.49	1	1
Southern European	Northern European	Siberian	322.61	1	1
Niger-Congo	Omotic	Arabian	323.57	1	1
Indian	Kalash	Pygmy	323.81	1	1
Indian	Arabian	Pygmy	323.88	1	1
Japanese	Northern European	Nilo-Saharan	324.17	1	1
Berber	Cushitic	Niger-Congo	324.41	1	1
Southern European	Levantine-Caucasian	Siberian	324.80	1	1
Northern European	Siberian	Nilo-Saharan	325.26	1	1
Pygmy	Omotic	Levantine-Caucasian	325.35	1	1
Khoisan	Omotic	Melanesian	325.61	1	1
Southeast Asian	Japanese	Berber	326.06	1	1
Nilo-Saharan	Omotic	Siberian	327.51	1	1
Native American	Siberian	Pygmy	327.55	1	1
Indian	Siberian	Nilo-Saharan	328.07	1	1
Arabian	Northern European	Japanese	328.11	1	1
Indian	Northern European	Melanesian	328.25	1	1
Levantine-Caucasian	Indian	Melanesian	329.15	1	1
Khoisan	Omotic	Southeast Asian	329.57	1	1
Niger-Congo	Omotic	Siberian	329.66	1	1
Khoisan	Pygmy	Berber	329.71	1	1
Chinese	Northern European	Pygmy	329.82	1	1
Cushitic	Northern European	Niger-Congo	329.88	1	1
Levantine-Caucasian	Indian	Chinese	330.65	1	1

Cushitic	Northern European	Melanesian	330.67	1	1
Khoisan	Cushitic	Chinese	330.78	1	1
Khoisan	Nilo-Saharan	Indian	331.50	1	1
Khoisan	Omotic	Japanese	331.83	1	1
Niger-Congo	Cushitic	Southeast Asian	332.10	1	1
Berber	Niger-Congo	Chinese	332.12	1	1
Native American	Northern European	Nilo-Saharan	332.26	1	1
Southern European	Kalash	Pygmy	332.44	1	1
Berber	Arabian	Japanese	332.66	1	1
Levantine-Caucasian	Kalash	Chinese	332.67	1	1
Berber	Cushitic	Melanesian	332.80	1	1
Berber	Siberian	Khoisan	332.87	1	1
Japanese	Chinese	Indian	332.93	1	1
Japanese	Chinese	Melanesian	333.30	1	1
Niger-Congo	Cushitic	Japanese	333.77	1	1
Niger-Congo	Northern European	Melanesian	334.26	1	1
Melanesian	Native American	Niger-Congo	334.60	1	1
Kalash	Arabian	Nilo-Saharan	335.41	1	1
Southeast Asian	Siberian	Levantine-Caucasian	335.69	1	1
Pygmy	Nilo-Saharan	Indian	335.69	1	1
Berber	Northern European	Native American	336.27	1	1
Arabian	Northern European	Southeast Asian	336.66	1	1
Berber	Levantine-Caucasian	Japanese	336.69	1	1
Cushitic	Indian	Khoisan	336.90	1	1
Khoisan	Niger-Congo	Kalash	337.08	1	1
Berber	Arabian	Southeast Asian	337.50	1	1
Khoisan	Pygmy	Kalash	338.16	1	1
Niger-Congo	Pygmy	Kalash	338.31	1	1
Chinese	Indian	Pygmy	338.46	1	1
Southeast Asian	Berber	Khoisan	338.55	1	1
Levantine-Caucasian	Cushitic	Native American	338.84	1	1
Cushitic	Northern European	Chinese	338.87	1	1
Levantine-Caucasian	Niger-Congo	Melanesian	340.33	1	1
Khoisan	Omotic	Levantine-Caucasian	340.34	1	1
Cushitic	Omotic	Chinese	341.31	1	1
Cushitic	Arabian	Melanesian	341.44	1	1
Southern European	Niger-Congo	Melanesian	341.64	1	1
Indian	Arabian	Nilo-Saharan	342.37	1	1
Berber	Levantine-Caucasian	Southeast Asian	342.42	1	1
Japanese	Native American	Pygmy	342.47	1	1
Pygmy	Omotic	Native American	342.48	1	1
Arabian	Native American	Niger-Congo	342.95	1	1
Melanesian	Siberian	Niger-Congo	343.32	1	1
Kalash	Northern European	Pygmy	343.55	1	1
Chinese	Northern European	Nilo-Saharan	343.67	1	1
Southeast Asian	Chinese	Omotic	343.76	1	1
Melanesian	Southeast Asian	Khoisan	344.13	1	1
Nilo-Saharan	Cushitic	Melanesian	344.22	1	1
Japanese	Native American	Southern European	344.36	1	1
Southern European	Cushitic	Native American	344.40	1	1
Southeast Asian	Indian	Nilo-Saharan	344.79	1	1

Levantine-Caucasian	Kalash	Pygmy	344.82	1	1
Pygmy	Omotic	Northern European	344.87	1	1
Berber	Cushitic	Native American	345.30	1	1
Cushitic	Arabian	Native American	346.48	1	1
Japanese	Siberian	Cushitic	346.69	1	1
Berber	Arabian	Pygmy	347.45	1	1
Berber	Japanese	Khoisan	347.45	1	1
Niger-Congo	Nilo-Saharan	Berber	348.51	1	1
Southern European	Indian	Pygmy	348.51	1	1
Japanese	Indian	Nilo-Saharan	349.12	1	1
Arabian	Northern European	Native American	349.38	1	1
Melanesian	Japanese	Khoisan	349.47	1	1
Levantine-Caucasian	Cushitic	Melanesian	349.74	1	1
Berber	Kalash	Niger-Congo	350.71	1	1
Southeast Asian	Arabian	Niger-Congo	351.53	1	1
Berber	Northern European	Pygmy	351.63	1	1
Pygmy	Nilo-Saharan	Siberian	352.13	1	1
Berber	Arabian	Melanesian	352.55	1	1
Arabian	Siberian	Niger-Congo	353.03	1	1
Southern European	Cushitic	Melanesian	354.78	1	1
Berber	Indian	Niger-Congo	354.91	1	1
Indian	Kalash	Nilo-Saharan	355.28	1	1
Berber	Levantine-Caucasian	Pygmy	355.50	1	1
Khoisan	Nilo-Saharan	Siberian	355.70	1	1
Southeast Asian	Siberian	Pygmy	355.91	1	1
Japanese	Siberian	Arabian	355.93	1	1
Levantine-Caucasian	Indian	Pygmy	356.28	1	1
Chinese	Native American	Pygmy	356.37	1	1
Chinese	Siberian	Cushitic	357.37	1	1
Pygmy	Omotic	Southern European	357.92	1	1
Berber	Arabian	Native American	357.98	1	1
Arabian	Native American	Khoisan	358.23	1	1
Levantine-Caucasian	Cushitic	Niger-Congo	358.75	1	1
Kalash	Native American	Khoisan	359.14	1	1
Southeast Asian	Chinese	Berber	359.24	1	1
Khoisan	Omotic	Native American	360.82	1	1
Chinese	Native American	Southern European	360.94	1	1
Berber	Northern European	Melanesian	361.13	1	1
Khoisan	Omotic	Northern European	361.42	1	1
Southeast Asian	Japanese	Cushitic	361.61	1	1
Indian	Northern European	Pygmy	361.64	1	1
Levantine-Caucasian	Arabian	Japanese	362.18	1	1
Nilo-Saharan	Omotic	Melanesian	362.25	1	1
Nilo-Saharan	Omotic	Southeast Asian	362.38	1	1
Southeast Asian	Native American	Nilo-Saharan	362.53	1	1
Berber	Levantine-Caucasian	Native American	362.56	1	1
Kalash	Siberian	Niger-Congo	362.72	1	1
Kalash	Native American	Niger-Congo	362.82	1	1
Japanese	Arabian	Niger-Congo	362.97	1	1
Southern European	Berber	Japanese	364.17	1	1
Nilo-Saharan	Cushitic	Native American	364.33	1	1

Kalash	Siberian	Khoisan	364.84	1	1
Southeast Asian	Kalash	Niger-Congo	365.22	1	1
Berber	Chinese	Khoisan	365.75	1	1
Chinese	Indian	Nilo-Saharan	365.87	1	1
Southern European	Kalash	Nilo-Saharan	365.90	1	1
Melanesian	Chinese	Khoisan	366.19	1	1
Levantine-Caucasian	Arabian	Southeast Asian	366.46	1	1
Chinese	Siberian	Arabian	367.44	1	1
Niger-Congo	Omotic	Southeast Asian	368.11	1	1
Berber	Levantine-Caucasian	Melanesian	368.30	1	1
Southern European	Berber	Southeast Asian	369.48	1	1
Arabian	Siberian	Khoisan	369.74	1	1
Niger-Congo	Omotic	Japanese	369.83	1	1
Southeast Asian	Kalash	Khoisan	370.15	1	1
Berber	Arabian	Nilo-Saharan	370.72	1	1
Southern European	Cushitic	Niger-Congo	371.23	1	1
Southeast Asian	Siberian	Southern European	371.29	1	1
Southern European	Indian	Nilo-Saharan	371.77	1	1
Cushitic	Arabian	Niger-Congo	372.41	1	1
Berber	Northern European	Nilo-Saharan	374.40	1	1
Niger-Congo	Cushitic	Melanesian	374.44	1	1
Japanese	Kalash	Niger-Congo	374.59	1	1
Niger-Congo	Omotic	Levantine-Caucasian	374.59	1	1
Khoisan	Nilo-Saharan	Arabian	374.72	1	1
Berber	Kalash	Khoisan	375.44	1	1
Khoisan	Omotic	Southern European	376.31	1	1
Melanesian	Southeast Asian	Niger-Congo	376.33	1	1
Southern European	Native American	Niger-Congo	376.68	1	1
Southeast Asian	Arabian	Khoisan	377.00	1	1
Niger-Congo	Omotic	Melanesian	377.57	1	1
Pygmy	Nilo-Saharan	Arabian	377.82	1	1
Southern European	Berber	Pygmy	378.16	1	1
Native American	Siberian	Nilo-Saharan	378.20	1	1
Khoisan	Niger-Congo	Indian	378.38	1	1
Japanese	Siberian	Northern European	378.71	1	1
Berber	Levantine-Caucasian	Nilo-Saharan	378.79	1	1
Niger-Congo	Pygmy	Indian	379.69	1	1
Japanese	Kalash	Khoisan	379.81	1	1
Levantine-Caucasian	Northern European	Japanese	380.98	1	1
Nilo-Saharan	Omotic	Japanese	381.15	1	1
Levantine-Caucasian	Kalash	Nilo-Saharan	381.51	1	1
Southern European	Arabian	Japanese	381.67	1	1
Melanesian	Japanese	Niger-Congo	381.79	1	1
Pygmy	Omotic	Chinese	381.89	1	1
Berber	Cushitic	Khoisan	382.43	1	1
Southeast Asian	Japanese	Arabian	382.50	1	1
Levantine-Caucasian	Native American	Niger-Congo	382.52	1	1
Levantine-Caucasian	Siberian	Niger-Congo	383.35	1	1
Pygmy	Nilo-Saharan	Melanesian	383.59	1	1
Kalash	Northern European	Nilo-Saharan	383.61	1	1
Southern European	Southeast Asian	Niger-Congo	383.99	1	1

Levantine-Caucasian	Indian	Nilo-Saharan	384.03	1	1
Cushitic	Northern European	Khoisan	384.47	1	1
Southern European	Siberian	Niger-Congo	384.69	1	1
Niger-Congo	Cushitic	Native American	384.72	1	1
Khoisan	Pygmy	Siberian	385.73	1	1
Chinese	Arabian	Niger-Congo	386.00	1	1
Southeast Asian	Levantine-Caucasian	Niger-Congo	386.40	1	1
Southern European	Arabian	Southeast Asian	386.65	1	1
Levantine-Caucasian	Cushitic	Chinese	386.71	1	1
Southern European	Native American	Khoisan	386.94	1	1
Southern European	Berber	Melanesian	387.26	1	1
Southern European	Berber	Native American	387.32	1	1
Levantine-Caucasian	Arabian	Native American	387.48	1	1
Levantine-Caucasian	Northern European	Southeast Asian	387.69	1	1
Khoisan	Pygmy	Indian	387.71	1	1
Japanese	Arabian	Khoisan	388.52	1	1
Arabian	Northern European	Melanesian	388.98	1	1
Nilo-Saharan	Omotic	Levantine-Caucasian	389.06	1	1
Berber	Cushitic	Chinese	389.30	1	1
Japanese	Chinese	Omotic	390.71	1	1
Levantine-Caucasian	Northern European	Native American	391.06	1	1
Niger-Congo	Pygmy	Siberian	391.36	1	1
Arabian	Northern European	Pygmy	392.55	1	1
Southern European	Cushitic	Chinese	392.63	1	1
Indian	Northern European	Nilo-Saharan	393.03	1	1
Chinese	Siberian	Northern European	393.53	1	1
Levantine-Caucasian	Native American	Khoisan	393.55	1	1
Cushitic	Arabian	Chinese	393.98	1	1
Chinese	Kalash	Niger-Congo	394.28	1	1
Southern European	Siberian	Khoisan	395.47	1	1
Pygmy	Nilo-Saharan	Southeast Asian	395.67	1	1
Southern European	Japanese	Niger-Congo	395.84	1	1
Nilo-Saharan	Omotic	Northern European	395.89	1	1
Levantine-Caucasian	Japanese	Niger-Congo	396.09	1	1
Nilo-Saharan	Omotic	Native American	396.19	1	1
Japanese	Siberian	Pygmy	396.39	1	1
Southeast Asian	Chinese	Cushitic	396.87	1	1
Southern European	Levantine-Caucasian	Japanese	396.89	1	1
Niger-Congo	Nilo-Saharan	Kalash	397.77	1	1
Pygmy	Nilo-Saharan	Japanese	398.10	1	1
Chinese	Kalash	Khoisan	398.24	1	1
Japanese	Native American	Nilo-Saharan	398.48	1	1
Japanese	Siberian	Levantine-Caucasian	398.53	1	1
Niger-Congo	Omotic	Northern European	398.62	1	1
Nilo-Saharan	Cushitic	Chinese	398.81	1	1
Khoisan	Niger-Congo	Siberian	399.45	1	1
Levantine-Caucasian	Siberian	Khoisan	399.82	1	1
Berber	Indian	Khoisan	400.73	1	1
Levantine-Caucasian	Arabian	Pygmy	401.67	1	1
Levantine-Caucasian	Arabian	Melanesian	402.17	1	1
Japanese	Chinese	Berber	402.21	1	1

Southern European	Northern European	Japanese	402.31	1	1
Southern European	Levantine-Caucasian	Southeast Asian	403.38	1	1
Khoisan	Pygmy	Melanesian	403.43	1	1
Khoisan	Nilo-Saharan	Melanesian	403.44	1	1
Cushitic	Arabian	Khoisan	403.80	1	1
Levantine-Caucasian	Cushitic	Khoisan	404.10	1	1
Melanesian	Chinese	Niger-Congo	404.16	1	1
Khoisan	Nilo-Saharan	Southeast Asian	404.66	1	1
Southern European	Arabian	Native American	405.03	1	1
Indian	Native American	Niger-Congo	405.09	1	1
Indian	Native American	Khoisan	405.21	1	1
Niger-Congo	Omotic	Native American	406.13	1	1
Southern European	Southeast Asian	Khoisan	406.44	1	1
Berber	Northern European	Chinese	406.53	1	1
Khoisan	Omotic	Chinese	406.63	1	1
Southern European	Northern European	Southeast Asian	406.77	1	1
Khoisan	Nilo-Saharan	Japanese	407.20	1	1
Southern European	Cushitic	Khoisan	407.60	1	1
Southeast Asian	Siberian	Nilo-Saharan	409.21	1	1
Chinese	Siberian	Pygmy	409.91	1	1
Nilo-Saharan	Omotic	Southern European	409.96	1	1
Southern European	Berber	Nilo-Saharan	410.89	1	1
Southeast Asian	Levantine-Caucasian	Khoisan	411.46	1	1
Southern European	Northern European	Native American	411.65	1	1
Chinese	Arabian	Khoisan	414.02	1	1
Southeast Asian	Japanese	Pygmy	414.10	1	1
Niger-Congo	Omotic	Southern European	415.16	1	1
Chinese	Native American	Nilo-Saharan	415.62	1	1
Chinese	Siberian	Levantine-Caucasian	416.31	1	1
Southeast Asian	Northern European	Niger-Congo	416.62	1	1
Southern European	Arabian	Melanesian	417.15	1	1
Indian	Siberian	Niger-Congo	417.45	1	1
Southern European	Japanese	Khoisan	418.12	1	1
Indian	Siberian	Khoisan	418.24	1	1
Berber	Arabian	Chinese	419.41	1	1
Southern European	Arabian	Pygmy	419.54	1	1
Southeast Asian	Japanese	Northern European	420.78	1	1
Southern European	Levantine-Caucasian	Native American	421.15	1	1
Niger-Congo	Pygmy	Melanesian	422.22	1	1
Pygmy	Nilo-Saharan	Native American	422.42	1	1
Levantine-Caucasian	Japanese	Khoisan	422.52	1	1
Kalash	Arabian	Khoisan	423.01	1	1
Southeast Asian	Chinese	Arabian	424.52	1	1
Niger-Congo	Cushitic	Chinese	425.26	1	1
Northern European	Siberian	Niger-Congo	425.54	1	1
Southern European	Chinese	Niger-Congo	426.33	1	1
Niger-Congo	Pygmy	Arabian	426.54	1	1
Levantine-Caucasian	Chinese	Niger-Congo	426.84	1	1
Northern European	Siberian	Khoisan	427.15	1	1
Khoisan	Niger-Congo	Arabian	427.17	1	1
Khoisan	Pygmy	Arabian	427.20	1	1

Kalash	Arabian	Niger-Congo	427.28	1	1
Southeast Asian	Northern European	Khoisan	431.26	1	1
Berber	Levantine-Caucasian	Chinese	431.31	1	1
Arabian	Northern European	Chinese	431.38	1	1
Southeast Asian	Japanese	Levantine-Caucasian	432.73	1	1
Japanese	Northern European	Niger-Congo	433.01	1	1
Arabian	Northern European	Nilo-Saharan	433.08	1	1
Native American	Northern European	Khoisan	434.43	1	1
Japanese	Siberian	Southern European	436.18	1	1
Pygmy	Nilo-Saharan	Levantine-Caucasian	436.65	1	1
Khoisan	Pygmy	Southeast Asian	437.19	1	1
Native American	Northern European	Niger-Congo	439.29	1	1
Niger-Congo	Nilo-Saharan	Indian	440.80	1	1
Khoisan	Nilo-Saharan	Native American	441.02	1	1
Indian	Kalash	Khoisan	441.03	1	1
Khoisan	Pygmy	Japanese	441.30	1	1
Levantine-Caucasian	Northern European	Pygmy	441.90	1	1
Niger-Congo	Pygmy	Southeast Asian	441.96	1	1
Japanese	Chinese	Cushitic	442.52	1	1
Indian	Arabian	Niger-Congo	442.58	1	1
Southeast Asian	Indian	Niger-Congo	442.88	1	1
Southeast Asian	Indian	Khoisan	443.51	1	1
Niger-Congo	Pygmy	Japanese	444.64	1	1
Southern European	Levantine-Caucasian	Pygmy	444.79	1	1
Levantine-Caucasian	Arabian	Nilo-Saharan	445.19	1	1
Japanese	Northern European	Khoisan	445.81	1	1
Khoisan	Nilo-Saharan	Levantine-Caucasian	446.04	1	1
Indian	Kalash	Niger-Congo	446.45	1	1
Southeast Asian	Chinese	Pygmy	449.01	1	1
Levantine-Caucasian	Northern European	Melanesian	449.34	1	1
Japanese	Indian	Niger-Congo	449.74	1	1
Khoisan	Pygmy	Native American	451.64	1	1
Japanese	Indian	Khoisan	451.86	1	1
Southern European	Chinese	Khoisan	453.18	1	1
Indian	Arabian	Khoisan	454.91	1	1
Southern European	Kalash	Khoisan	455.26	1	1
Southeast Asian	Native American	Khoisan	455.59	1	1
Niger-Congo	Omotic	Chinese	455.78	1	1
Chinese	Siberian	Southern European	457.16	1	1
Levantine-Caucasian	Chinese	Khoisan	457.18	1	1
Khoisan	Niger-Congo	Southeast Asian	458.35	1	1
Khoisan	Niger-Congo	Melanesian	458.42	1	1
Pygmy	Nilo-Saharan	Northern European	458.62	1	1
Native American	Siberian	Khoisan	458.96	1	1
Khoisan	Niger-Congo	Japanese	459.03	1	1
Southern European	Levantine-Caucasian	Melanesian	459.99	1	1
Southern European	Northern European	Pygmy	460.52	1	1
Nilo-Saharan	Omotic	Chinese	462.90	1	1
Japanese	Siberian	Nilo-Saharan	465.29	1	1
Southern European	Berber	Chinese	465.99	1	1
Niger-Congo	Nilo-Saharan	Siberian	466.07	1	1

Berber	Arabian	Niger-Congo	466.10	1	1
Southern European	Kalash	Niger-Congo	466.76	1	1
Niger-Congo	Pygmy	Native American	466.95	1	1
Southeast Asian	Native American	Niger-Congo	467.66	1	1
Chinese	Northern European	Niger-Congo	468.66	1	1
Levantine-Caucasian	Arabian	Chinese	469.93	1	1
Khoisan	Nilo-Saharan	Northern European	470.31	1	1
Levantine-Caucasian	Kalash	Khoisan	470.52	1	1
Southern European	Arabian	Nilo-Saharan	470.77	1	1
Kalash	Northern European	Khoisan	470.89	1	1
Berber	Northern European	Niger-Congo	471.49	1	1
Japanese	Chinese	Arabian	473.84	1	1
Southeast Asian	Japanese	Southern European	475.82	1	1
Southeast Asian	Chinese	Northern European	476.16	1	1
Berber	Arabian	Khoisan	476.77	1	1
Southern European	Northern European	Melanesian	477.05	1	1
Chinese	Indian	Niger-Congo	479.33	1	1
Berber	Levantine-Caucasian	Niger-Congo	480.10	1	1
Native American	Siberian	Niger-Congo	480.26	1	1
Chinese	Indian	Khoisan	481.16	1	1
Pygmy	Nilo-Saharan	Southern European	481.55	1	1
Chinese	Siberian	Nilo-Saharan	483.60	1	1
Chinese	Northern European	Khoisan	484.13	1	1
Southeast Asian	Japanese	Nilo-Saharan	484.62	1	1
Levantine-Caucasian	Kalash	Niger-Congo	485.16	1	1
Japanese	Chinese	Pygmy	485.26	1	1
Kalash	Northern European	Niger-Congo	485.35	1	1
Southern European	Indian	Niger-Congo	486.29	1	1
Southeast Asian	Chinese	Levantine-Caucasian	486.65	1	1
Japanese	Native American	Khoisan	487.53	1	1
Berber	Northern European	Khoisan	490.37	1	1
Southern European	Indian	Khoisan	491.79	1	1
Pygmy	Nilo-Saharan	Chinese	493.04	1	1
Khoisan	Niger-Congo	Native American	494.57	1	1
Berber	Levantine-Caucasian	Khoisan	494.63	1	1
Niger-Congo	Pygmy	Levantine-Caucasian	495.24	1	1
Khoisan	Nilo-Saharan	Southern European	495.73	1	1
Khoisan	Pygmy	Levantine-Caucasian	496.40	1	1
Niger-Congo	Nilo-Saharan	Arabian	496.57	1	1
Southeast Asian	Siberian	Khoisan	496.69	1	1
Southern European	Arabian	Chinese	496.91	1	1
Levantine-Caucasian	Northern European	Nilo-Saharan	497.05	1	1
Levantine-Caucasian	Indian	Niger-Congo	498.34	1	1
Southern European	Levantine-Caucasian	Nilo-Saharan	499.73	1	1
Levantine-Caucasian	Indian	Khoisan	502.42	1	1
Levantine-Caucasian	Northern European	Chinese	502.59	1	1
Indian	Northern European	Niger-Congo	507.12	1	1
Japanese	Native American	Niger-Congo	510.20	1	1
Indian	Northern European	Khoisan	511.04	1	1
Khoisan	Niger-Congo	Levantine-Caucasian	512.48	1	1
Chinese	Native American	Khoisan	515.08	1	1

Khoisan	Pygmy	Northern European	515.23	1	1
Niger-Congo	Pygmy	Northern European	517.21	1	1
Southern European	Berber	Niger-Congo	517.82	1	1
Southeast Asian	Siberian	Niger-Congo	518.23	1	1
Southern European	Berber	Khoisan	518.99	1	1
Khoisan	Nilo-Saharan	Chinese	520.15	1	1
Southern European	Northern European	Nilo-Saharan	523.56	1	1
Southern European	Levantine-Caucasian	Chinese	526.28	1	1
Niger-Congo	Nilo-Saharan	Southeast Asian	526.41	1	1
Southeast Asian	Chinese	Nilo-Saharan	530.47	1	1
Niger-Congo	Nilo-Saharan	Melanesian	531.84	1	1
Niger-Congo	Nilo-Saharan	Japanese	532.05	1	1
Southern European	Northern European	Chinese	532.75	1	1
Japanese	Chinese	Northern European	533.02	1	1
Southeast Asian	Chinese	Southern European	536.64	1	1
Khoisan	Niger-Congo	Northern European	537.18	1	1
Khoisan	Pygmy	Southern European	537.65	1	1
Chinese	Native American	Niger-Congo	544.21	1	1
Japanese	Chinese	Levantine-Caucasian	544.26	1	1
Arabian	Northern European	Khoisan	544.94	1	1
Niger-Congo	Pygmy	Southern European	545.05	1	1
Khoisan	Pygmy	Chinese	546.22	1	1
Japanese	Siberian	Khoisan	546.68	1	1
Niger-Congo	Pygmy	Chinese	556.36	1	1
Levantine-Caucasian	Arabian	Khoisan	556.83	1	1
Arabian	Northern European	Niger-Congo	562.11	1	1
Niger-Congo	Nilo-Saharan	Native American	566.99	1	1
Khoisan	Niger-Congo	Southern European	567.40	1	1
Chinese	Siberian	Khoisan	572.01	1	1
Southern European	Arabian	Khoisan	575.57	1	1
Southeast Asian	Japanese	Khoisan	577.47	1	1
Levantine-Caucasian	Arabian	Niger-Congo	578.60	1	1
Japanese	Siberian	Niger-Congo	584.59	1	1
Niger-Congo	Nilo-Saharan	Levantine-Caucasian	585.48	1	1
Khoisan	Niger-Congo	Chinese	594.61	1	1
Japanese	Chinese	Southern European	596.86	1	1
Japanese	Chinese	Nilo-Saharan	599.40	1	1
Southern European	Arabian	Niger-Congo	611.10	1	1
Niger-Congo	Nilo-Saharan	Northern European	614.69	1	1
Chinese	Siberian	Niger-Congo	616.72	1	1
Southeast Asian	Japanese	Niger-Congo	617.62	1	1
Levantine-Caucasian	Northern European	Khoisan	617.78	1	1
Southern European	Levantine-Caucasian	Khoisan	618.55	1	1
Southeast Asian	Chinese	Khoisan	632.56	1	1
Southern European	Northern European	Khoisan	638.35	1	1
Niger-Congo	Nilo-Saharan	Southern European	647.37	1	1
Levantine-Caucasian	Northern European	Niger-Congo	648.81	1	1
Southern European	Levantine-Caucasian	Niger-Congo	655.65	1	1
Niger-Congo	Nilo-Saharan	Chinese	674.73	1	1
Japanese	Chinese	Khoisan	677.52	1	1
Southern European	Northern European	Niger-Congo	684.10	1	1

Southeast Asian	Chinese	Niger-Congo	685.55	1	1
Japanese	Chinese	Niger-Congo	747.80	1	1
